# Evidence that S‐phase kinase associated protein 2 (SKP2) is ubiquitinated and degraded via a Rho‐related BTB domain containing 1 (RhoBTB1) and Cullin‐3 mechanism in placenta

**DOI:** 10.14814/phy2.71039

**Published:** 2026-08-04

**Authors:** Alina L. Tvina, M. Christine Livergood, Gaurav Kumar, Valerie W. Hole, Megan A. Opichka, Luisangeli Muniz Irizarry, Ko‐Ting Lu, Kelsey K. Wackman, Justin L. Grobe, Pablo Nakagawa, Jennifer J. McIntosh, Curt D. Sigmund

**Affiliations:** ^1^ Department of Obstetrics and Gynecology, Cardiovascular Center Medical College of Wisconsin Milwaukee Wisconsin USA; ^2^ Department of Physiology, Cardiovascular Center Medical College of Wisconsin Milwaukee Wisconsin USA

**Keywords:** placenta, proteomics, RhoBTB1, SKP2, ubiquitination

## Abstract

Rho related BTB domain containing 1 (RhoBTB1) is highly expressed in placenta and functions to deliver protein targets to the Cullin‐3 (CUL3) E3 ubiquitin ligase where they are targeted for ubiquitination and degradation. The targets of RhoBTB1 in placenta have not been identified. Using RNAscope, we show that RhoBTB1 is mainly expressed in syncytiotrophoblasts (SCT) in the human and mouse placenta. We employed ascorbate peroxidase 2‐mediated targeted proteomics to identify RhoBTB1 binding proteins in immortalized human extravillous trophoblast (HTR8/SVneo) cells. We selected 9 RhoBTB1‐interacting proteins to examine functionally. Two of these, S‐Phase Kinase Associated Protein 2 (SKP2) and Rho GTPase‐Activating Protein 29 (ArhGAP29), increased in abundance when Cullin activity was blocked by the neddylation inhibitor MLN4924 and co‐immunoprecipitated with RhoBTB1. SKP2 increased in abundance in CRISPR‐Cas9 HEK293 cells that lack CUL3, and in HTR8/SVneo cells after siRNA‐mediated inhibition of RhoBTB1. SKP2 was ubiquitinated by a RhoBTB1‐ and CUL3‐dependent mechanism providing evidence that its stability is regulated by RhoBTB1/CUL3. Like RhoBTB1, SKP2 is highly expressed in the placenta. Reanalysis of single cell RNA sequencing data sets revealed that SKP2 exhibits co‐expression with RhoBTB1 in SCT precursor cells, SCTs, and cytotrophoblasts. These findings identify SKP2 as a RhoBTB1/CUL3 target in the placenta.

## Graphical abstract

Graphical representation of mechanism by which RhoBTB1 regulates SKP2.

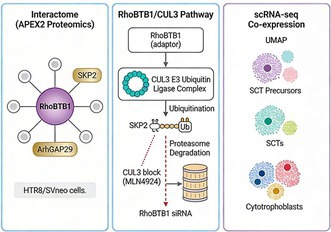

## 1 INTRODUCTION

The placenta is an essential and highly specialized organ allowing for the exchange of nutrients, gases, and wastes between mother and fetus. Normal placental development is critical to establish and maintain a healthy pregnancy, whereas impairment of placental function can result in poor pregnancy. Placental syncytiotrophoblast (SCT) cells are an important cell type at the maternal‐fetal interface. They orchestrate the complex interactions between fetus and mother and serve as an important endocrine organ that produces numerous growth factors and hormones that support and regulate placental and fetal development and growth (Carrasco‐Wong et al., 2021). Despite its importance, a full understanding of placental and SCT biology remains lacking.

Peroxisome Proliferator‐Activated Receptor gamma (PPARγ) is a transcription factor implicated in blood pressure regulation and in the pathogenesis of hypertension and is required for the appropriate development of the placenta (Barak et al., 1999; Fang et al., 2021). PPARγ‐deficient mice exhibit multiple placental defects including malformed labyrinth zone, lack of infiltration of fetal blood vessels, and rupture of maternal blood sinuses (Barak et al., 1999). In humans, several studies have linked PPARγ to normal placental development. For example, the accumulation of lipid droplets when cytotrophoblasts differentiate into SCT is impaired in PPARγ‐deficient placentas (Tarrade et al., 2001). RhoBTB1 is an important PPARγ target gene that has been genetically correlated with hypertension (Evangelou et al., 2018; Newton‐Cheh et al., 2009; Pelham et al., 2012). PPARγ interference in vascular smooth muscle cells results in elevated blood pressure, endothelial dysfunction, and arterial stiffness, concomitant with RhoBTB1 downregulation (Halabi et al., 2008; Pelham et al., 2012). Restoration of RhoBTB1 expression lowers blood pressure, improves vascular function, and causes regression of arterial stiffness (Mukohda et al., 2019). Similarly, expression of RhoBTB1 in angiotensin‐II induced hypertension causes a rapid reversal of arterial stiffness (Fang et al., 2022).

RhoBTB1 is an atypical member of the Rho family of small guanosine triphosphatases (GTPases) (Fang et al., 2021). It is a multi‐domain protein that has an N‐terminal, GTPase domain, followed by a proline‐rich region, a tandem of 2 broad‐complex, tramtrack, bric à brac (BTB) domains, and a conserved C‐terminal region (Kumar et al., 2023). BTB domains are mediators of protein–protein interaction and serve as the distinctive feature of adaptor proteins that direct protein substrates towards ubiquitination via the Cullin‐3 (CUL3)‐dependent Cullin Ring Ligase (CRL3) complex. RhoBTB1 interacts with CUL3 through its first BTB domain and with substrates through its C‐terminal (C) domain (Kumar et al., 2023). Like RhoBTB1, CUL3 has been genetically linked with hypertension (Boyden et al., 2012). In the vessel wall, CUL3 regulates key proteins involved in maintaining vascular homeostasis (Agbor et al., 2019; Ibeawuchi et al., 2015; Mukohda et al., 2019; Wu et al., 2022).

Interestingly, data from the Human Protein Atlas (proteinatlas.org) indicates that one of the organs with the highest level of RhoBTB1 expression in humans is the placenta (the other being skeletal muscle), and single cell sequencing revealed that RhoBTB1 expression is specifically localized to SCT cells (Lizio et al., 2015; Nobusada et al., 2025; Uhlén et al., 2015). Thus, the goal of this study was to identify and characterize novel RhoBTB1‐target proteins in placenta and to assess if they are regulated by CUL3‐mediated ubiquitination. Placentas from normal and those with hypertensive disorders of pregnancy were used given the RhoBTB1 and CUL3 link with hypertension. After identifying a series of potential binding partners, we validated one of these, S‐Phase Kinase Associated Protein 2 (SKP2). Like RhoBTB1, SKP2 is abundantly expressed in the placenta and is regulated in a RhoBTB1/CUL3‐dependent manner. RhoBTB1 and SKP2 also exhibit an overlapping pattern of cellular expression in the placenta including in SCTs.

## 2 METHODS

### 2.1 Study approval

All protocols were approved by the Animal Care and Use Committee and Institutional Review Board at the Medical College of Wisconsin. Surgical and experimental procedures adhered to the National Institutes of Health's Guide for the Care and Use of Laboratory Animals. We selected human placental samples from the Medical College of Wisconsin's Maternal Research Placental and Cord Blood Bank (IRB Approvals PRO00035848 and PRO00029926). Written consent was required and obtained from all participating subjects.

### 2.2 Placental sample selection

Samples were selected if they underwent a cesarean birth without labor and carried no additional co‐morbidities beyond hypertensive disorders of pregnancy. Patients were matched among cohorts by gestational age, maternal age, body mass index, and presence or absence of chronic hypertension. In addition, the patient's gravidity and parity and placental pathology results were collected. We collected placenta from term and preterm pregnancies with and without a diagnosis of preeclampsia. The diagnosis of preeclampsia was based on the standard American College of Obstetrics and Gynecology definitions and the electronic medical record was evaluated to confirm the accuracy of the diagnosis (Obstetricians ACo & Gynecologists, 2020). Term was defined as gestational age greater than or equal to 37 weeks gestation at time of delivery and preterm was anything prior to 37 weeks of gestation. Placental samples were sent to the histology lab where tissue was fixed in formalin and embedded in paraffin. Additional placental samples were snap frozen in liquid nitrogen. All banked placental samples for this study were obtained, and sections (2–3 consecutive sections per slide) were taken from the maternal surface, fetal surface, membrane and umbilical cord and are collected at a consistent, uniform location from the umbilical cord insertion. All samples included in this analysis were formalin‐fixed, paraffin embedded from the maternal surface. A total of 9 samples were subjected to RNAscope in situ hybridization (Term Control *n* = 3; Preterm non‐Pre *n* = 2; Term PreE *n* = 2; Preterm PreE *n* = 2). We obtained 4–10 images per section at various magnifications.

Mouse placenta was obtained from C57BL/6J control mice at gestational days 14.5 and 17.5 (Jackson Laboratory), housed at 22°C on a 14:10 light cycle with ad libitum access to Inotiv 2920× chow and filtered/chlorinated water as previously described in detail (Lawton et al., 2024). Mice were euthanized by CO_2_, and tissues were fixed in 10% neutral buffered formalin and embedded in paraffin by the Histology Core at Children's Research Institute of Medical College of Wisconsin and Children's Hospital of Wisconsin.

### 2.3 RNAScope

In situ hybridization (ISH) was performed using RNAScope as per the manufacturer's (ACD Bio) instructions and as previously described (Opichka et al., 2023; Opichka et al., 2025). To validate the use of the *RhoBTB1* in the ISH probe, a *RhoBTB1* scrambled probe was examined, but no signal was observed in the tissue specimens (data not shown). A positive and negative control were utilized in all replicates. In human placenta, platelet‐endothelial cell adhesion molecule‐1 (*PECAM1*) was used as a marker for endothelium, and melanoma cell adhesion molecule (*MCAM*) and *CYP19A1*, a gene encoding cytochrome P450 enzyme, were used as markers for the SCT cell layer (Uhlén et al., 2015). Several co‐localization markers were explored in the murine placenta. *Slc16a1* encodes for MCT1 and is specific for syncytiotrophoblast layer I (SynI). *Prl8a8* (PlpCγ) is a marker for murine spongiotrophoblast. *Pecam1* (CD31) is a specific marker for fetal endothelium (Croy Bay et al., 2014). We used a red chromogenic marker for *RhoBTB1*, and a cyan probe was used for the cell‐specific co‐localization marker. Images were captured and processed with a Keyence BZ‐X series microscope.

### 2.4 Cell culture

HTR‐8/SVneo cells (human female placental trophoblast cells, ATCC) (Lapehn et al., 2025) were cultured in Dulbecco's modified Eagle medium (11885084, Gibco, Thermo Fisher Scientific, USA) containing 5% fetal bovine serum (10082147, Gibco, Thermo Fisher Scientific, USA) and 1% penicillin–streptomycin (15140122, Gibco, Thermo Fisher Scientific, USA) and maintained in a humidified CO_2_ incubator at 37°C with 5% CO_2_ as described previously (Kumar et al., 2023). Cells were washed with Dulbecco's phosphate buffered saline (DPBS, 14190144, Gibco, Thermo Fisher Scientific, USA) and harvested using 0.05% trypsin–EDTA (25300054, Gibco, Thermo Fisher Scientific, USA) for subsequent sub‐culturing.

### 2.5 Biotinylation and proximity labeling by APEX2

APEX2 proximity labelling was done as performed earlier with minor modifications (Kumar et al., 2023). HTR8/SVneo cells transfected with appropriate constructs were maintained as above. Cells were treated with an inhibitor of neddylation which acts as a pan‐Cullin inhibitor (MLN4924, 1 μM, 951950‐33‐7, Calbiochem, USA) and with the proteasomal inhibitor MG132 (10 μM, M7449, Sigma‐Aldrich, USA) after 16 h and 20 h of transfection, respectively. Next, cells were incubated in 500 μM biotin phenol (SML2135, Sigma‐Aldrich, USA) reconstituted in complete media at 37°C for 30 min and then were exposed to flash exposure of 1 mM H_2_O_2_ (H1009, Sigma‐Aldrich, USA) for 1 min for biotinylation at room temperature in the dark. The biotinylation reaction was rapidly quenched after 1 min using three washes of ice‐cold DPBS containing quenchers: 5 mM Trolox (238813, Sigma‐Aldrich, USA), 10 mM sodium ascorbate (PHR1279, Sigma‐Aldrich, USA), and 10 mM sodium azide (190385000, Thermo Fisher Scientific, USA). Cells were then harvested and lysed on ice bath using RIPA lysis buffer containing 1 mM phenylmethylsulfonyl fluoride (PMSF), protease inhibitor cocktail (78430, Thermo Fisher Scientific, USA), and above‐mentioned reaction quenchers and then centrifuged at 15,000 *g* at 4°C for 10 min. Biotinylated proteome of HTR‐8/SVneo cells was enriched by affinity capture using streptavidin magnetic beads (88817, Thermo Fisher Scientific, USA) through overnight incubation of whole‐cell lysates with gentle rotation at 4°C. Beads were subsequently washed with the following buffers to remove non‐specific binders: RIPA buffer (3 washes), 1 M KCl (P41025‐500.0, RPI Research Products Int., USA), 0.1 M Na_2_CO_3_ (L13098.36, Thermo Fisher Scientific, USA), 2 M Urea (15505035, Thermo Fisher Scientific, USA), in 10 mM Tris (pH 8.0, T5941, Sigma‐Aldrich, USA), and RIPA buffer (3 washes). We have previously validated the functional efficacy of the fusion constructs and confirmed that the addition of the APEX2 tag to RhoBTB1 domains does not interfere in recruiting the potential binding partners of RhoBTB1 (Kumar et al., 2023).

### 2.6 Mass spectrometry

For the identification of biotinylated proteome of HTR‐8/SVneo cells, streptavidin pulldown beads bearing enriched biotinylated proteome were immobilized on a magnetic rack, the suspension buffer was removed, and beads were resuspended in 100 μL of 40% Invitrosol (MS1000, Invitrogen, Thermo Fisher Scientific, USA) prepared in 100 mM ammonium bicarbonate (09830, Fluka, Switzerland) as described previously (Kumar et al., 2023). Cysteines were reduced with 5 mM Tris (2‐carboxyethyl)phosphine hydrochloride (TCEP, 646547, Sigma‐Aldrich, USA) for 30 min at 37°C and alkylated with 10 mM iodoacetamide (100,351, MP Biomedicals, USA) for an additional 30 min at 37°C. Proteins bound to the streptavidin beads were digested directly on‐beads overnight at 37°C using 5 μg of sequencing grade modified trypsin (PR‐V5113, Promega, Thermo Fisher Scientific, USA). Peptides were cleaned using the SP2 protocol. Thermo Scientific Pierce Peptide Retention Time Calibration Mixture (88321, Pierce, Thermo Fisher Scientific, USA) was added at a final concentration of 4 nM during dilution of the samples to 20 ng/μL for retention time normalization.

Samples were analyzed using a Thermo Scientific Orbitrap Fusion Lumos mass spectrometer using data‐dependent acquisition (DDA) with HCD MS2, performed using two biological replicates (defined as independent transfections) each of B1B2 and B1B2C. Three technical replicates of each biological replicate sample were injected. Instrument settings are as previously reported but reproduced here for convenience detailed in Table S1 (Kumar et al., 2026). Technical replicates were blocked and randomized to minimize bias. A pooled quality control (QC) sample, comprising equal portions of all experimental samples, was run at the beginning, end, and between each block of technical replicates in the acquisition sequence to monitor instrument performance and consistency. Raw MS data were processed using Proteome Discoverer v2.4 (Thermo), as described previously and reproduced here Table S2 (Kumar et al., 2026). Protein identification required at least two unique peptides per protein for inclusion in downstream analysis. Mass spectrometry proteomics data have been deposited to the Proteome Xchange Consortium via the PRIDE partner repository with the dataset identifier PXD073804 and 10.6019/PXD073804.

### 2.7 Western blotting

Cells were washed and lysed using ice cold RIPA lysis buffer (50 mM Tris–HCl (pH 7.5), 150 mM NaCl, 1 mM Na_2_EDTA, 1 mM EGTA, 1% NP‐40, 1% sodium deoxycholate, 2.5 mM sodium pyrophosphate, 0.1% SDS, 1 mM β‐glycerophosphate, 1 mM Na_3_VO_4_), containing protease and phosphatase inhibitor (78442, Thermo Fisher Scientific, USA), and 1 mM PMSF (36978, Thermo Fisher Scientific, USA) reconstituted in 2‐propanol (I9030, Sigma–Aldrich, USA). Next, cells were collected and gently rotated for 10 min at 4°C, and whole‐cell lysates were prepared by centrifuging the cell homogenates at 13,000 g for 10 min at 4°C. 50 μg of protein lysates were resolved using SDS‐polyacrylamide gel electrophoresis (PAGE) (4%–20%) and transferred to nitrocellulose membranes (88018, Thermo Fisher Scientific, USA). Following transfer, membranes were blocked by incubation in 3% BSA in 1× tris‐buffered saline (TBS, 1706435, Bio‐Rad Laboratories, USA) containing 0.1% Tween‐20 (0.1% TBST, P1379, Sigma–Aldrich, USA) for 1 h at room temperature. Following blocking, membranes were incubated with primary antibodies at 4°C overnight. The following primary antibodies were used to detect target proteins: rabbit anti‐ArhGAP29 (12583‐1‐AP, Proteintech, USA), rabbit anti‐WIPI2 (28820‐1‐AP, Proteintech, USA), rabbit anti‐PPP1CB (10140‐2‐AP, Proteintech, USA), rabbit anti‐MAEA (28363‐1‐AP, Proteintech, USA), rabbit anti‐PSMD1 (17709‐1‐AP, Proteintech, USA), rabbit anti‐PSMD4 (14899‐1‐AP, Proteintech, USA), rabbit anti‐PSMD13 (15261‐1‐AP, Proteintech, USA), rabbit anti‐AIFM1 (17984‐1‐AP, Proteintech, USA), mouse anti‐HSP90 (60318‐1‐Ig, Proteintech, USA), rabbit anti‐CUL‐3 (10450S, Cell Signaling Technology, USA), and rabbit anti‐RhoBTB1 (PA5‐42077, Invitrogen, Thermo Fisher Scientific, USA). The next day, membranes were incubated with horseradish peroxidase (HRP)‐linked secondary antibodies (7074S and 7076S, Cell Signaling Technology, USA) at a 1:3000 dilution in 0.1% TBST for 1 h at room temperature and developed with Clarity Western ECL reagent (BioRad, 1705061).

### 2.8 Co‐immunoprecipitation

For Co‐IP, HTR87/SV‐neo were treated with 1 μM MLN4924 and 10 μM MG132 after transfection with appropriate constructs as described earlier (Kumar et al., 2023). 24 h after transfection, the cells were washed with ice‐cold DPBS (J67802‐K2, Thermo Fisher Scientific, USA) and lysed on ice using IP lysis buffer (20 mM Tris–HCl (pH 7.5), 150 mM NaCl, 1 mM Na_2_EDTA, 1 mM EGTA, 1% Triton X‐100, 2.5 mM sodium pyrophosphate, 1 mM β‐glycerophosphate, 1 mM Na_3_VO_4_, 1 μg/mL leupeptin, and 1 mM PMSF reconstituted in isopropanol). Cells were collected and gently rotated for 30 min at 4°C. The supernatants were then collected by centrifuging the cell homogenate at 13,000 g for 10 min at 4°C. 1000 μg of protein lysates were incubated with 20 μL of mouse anti‐myc Sepharose bead conjugate (3400S, Cell Signaling Technology, USA) overnight at 4°C with gentle rotation. The beads were collected by centrifugation at 0.5 g for 5 min at 4°C and washed 3 times (2 min each wash) with IP washing buffer (20 mM Tris–HCl (pH 7.5), 150 mM NaCl, 0.5 mM EDTA) at 4°C with gentle rotation. Immunocomplexes were then dissociated by boiling in 4× LDS sample buffer (NP0007, Invitrogen, Thermo Fisher Scientific, USA), resolved by SDS‐PAGE, and transferred to a nitrocellulose membrane. Membranes were blocked in EasyBlocker (GTX425858, GeneTex, Inc., USA), reconstituted in 0.1% TBST for 1 h at room temperature and then incubated with appropriate primary antibodies. The following antibodies were used: rabbit anti‐ArhGAP29 (12583‐1‐AP, Proteintech, USA), rabbit anti‐SKP2 (15010‐1‐AP, Proteintech, USA), rabbit anti‐myc (2278S, Cell Signaling Technology, USA), rabbit anti‐ubiquitin (43124S, Cell Signaling Technology, USA), rabbit anti‐his (2365S, Cell Signaling Technology, USA), and mouse anti‐HSP90 (60318‐1‐Ig, Proteintech, USA). The next day, membranes were incubated with HRP‐conjugated EasyBlot secondary antibodies (GTX221666‐01 and GTX221667‐01, GeneTex, Inc., USA) at 1:3000 dilution in 0.1% TBST for 1 h at room temperature developed with Clarity Western ECL reagent (BioRad, 1705061).

### 2.9 Transfection and RNA interference

For the expression of myc epitope‐tagged and myc‐APEX2‐tagged B1B2 and B1B2C domains of RhoBTB1, HTR8/SVneo cells were transfected using Lipofectamine LTX‐PLUS reagent (A12621, Thermo Fisher Scientific, USA) for 24 h according to the manufacturer's protocol. Cells in the control sets were transfected with pcDNA3.1 mammalian expression vector used for cloning of RhoBTB1 domains. For RNA interference, SMARTPool siRNA targeting RhoBTB1 (L‐009389‐00‐0010, Horizon Discovery, USA) was transfected into HTR‐8/SVneo cells using DharmaFECT transfection reagent #1 (T‐2001‐03, Horizon Discovery, USA) for 72 h according to the manufacturer's instructions. Cells in the control sets were transfected with negative control pool of 4 siRNAs (D‐001810‐10‐05, Horizon Discovery).

### 2.10 Ubiquitination assay

Transfected HEK293^WT^ and HEK293^CUL3KO^ cells were treated with MG132 (M7449, Sigma‐Aldrich, USA) or MLN4924 (951950‐33‐7, Calbiochem, USA) immediately after transfection with appropriate constructs. Next, whole cell lysates were prepared in ice‐cold RIPA lysis buffer to maintain the reducing environment to avoid bystander pull down of RhoBTB1 (Mukohda et al., 2019). Immunoprecipitation was performed. Immunocomplexes were resolved and blots were developed using the methodology mentioned above.

### 2.11 Bioinformatics

Analysis of publicly available human placenta scRNAseq datasets was conducted in R‐4.4.2 (https://www.r‐project.org/). 10× Genomics cell ranger matrix files from GSE174399, GSE174481, and GSE198373 were downloaded from the Gene Expression Omnibus (GEO) (Marsh et al., 2022; Shannon et al., 2022; Zheng et al., 2022). Datasets were analyzed using Seurat 5.0.0 R package following quality control settings as described in the original publications (Hao et al., 2024). Cell type identities were assigned based on cell type markers used in the original publications. Gene expression correlations between RhoBTB1 and SKP2 were calculated using distance correlation, and directionality of the RhoBTB1‐SKP2 relationship was determined by Spearman's correlation (Hou et al., 2022).

## 3 RESULTS

**FIGURE 1:**
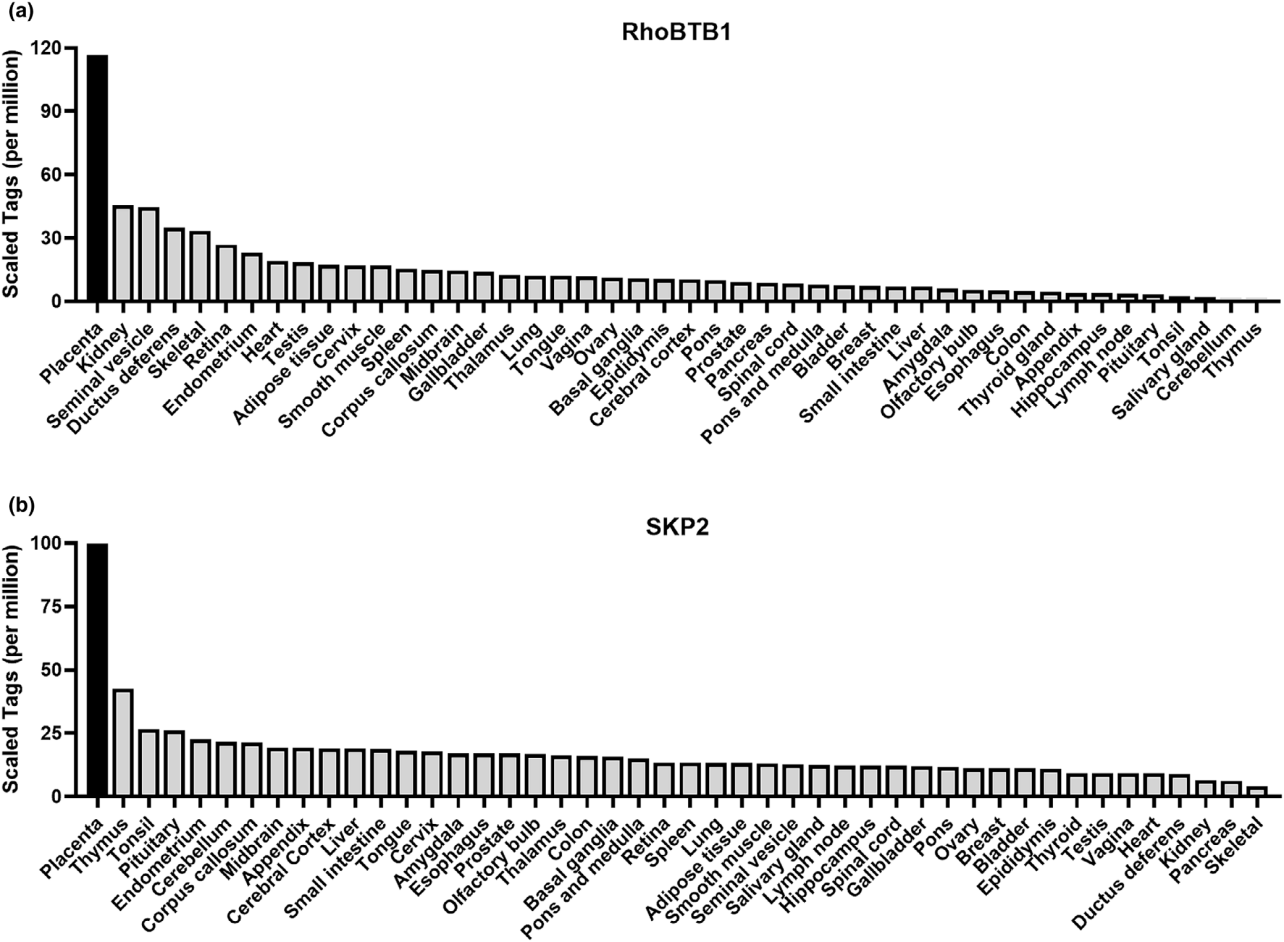
Relative expression of RhoBTB1 (a) and SKP2 (b) in human tissues. Relative expression in tags per million was re‐plotted from the FANTOM5 dataset from The Human Protein Atlas (Takahashi et al., 2012). Data is ordered from highest to lowest expression. The order of the tissues is different for RhoBTB1 and SKP2.

**FIGURE 2:**
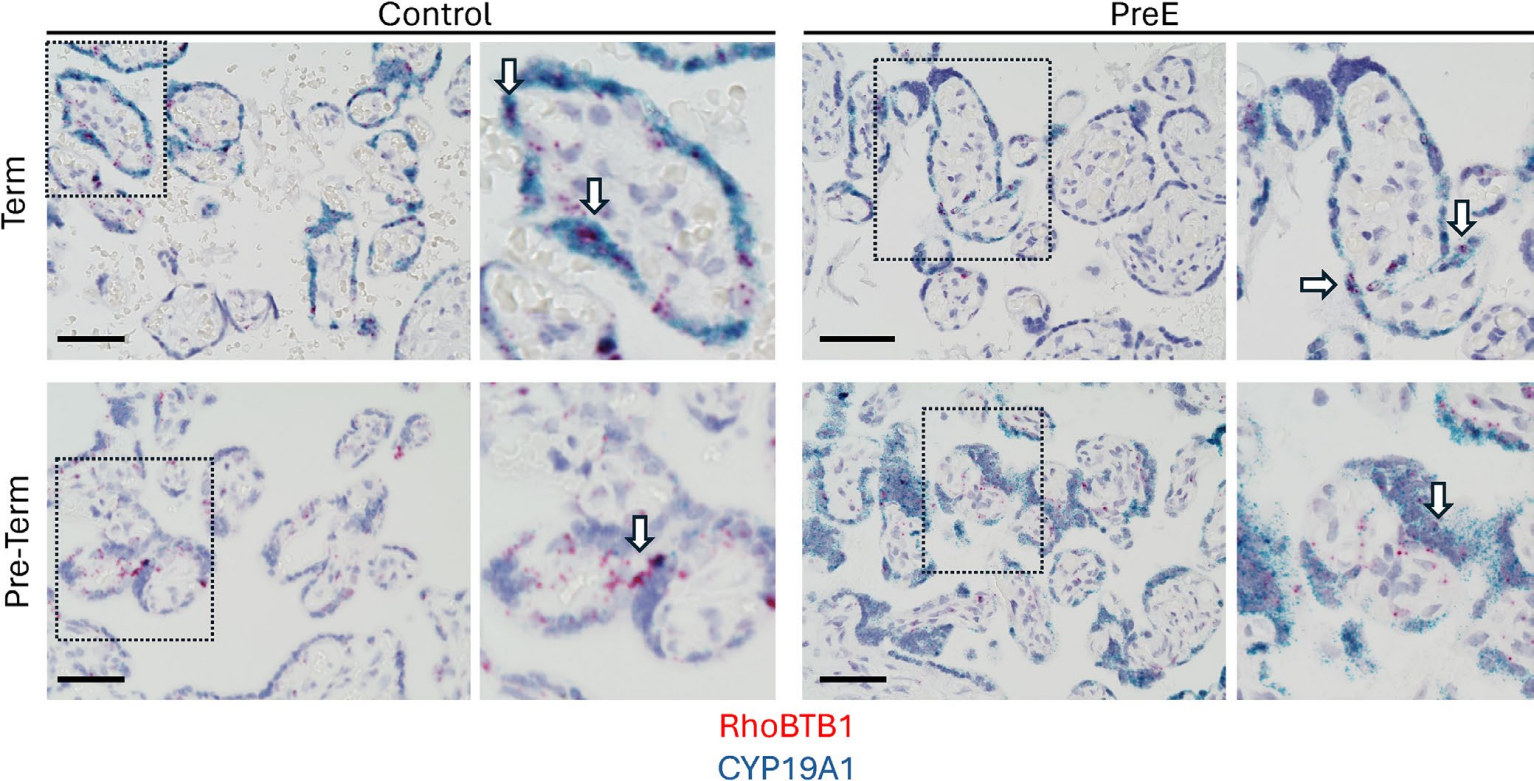
Expression of RhoBTB1 in syncytiotrophoblasts in human placenta. Representative RNAScope in situ hybridization of human placental samples (maternal side) from term and preterm control (healthy) and preeclamptic (PreE) pregnancies demonstrating co‐localization of RhoBTB1 (red puncta) with CYP19A (blue), a SCT marker. Scale bar is 50 μm. The region inside the dotted rectangle is expanded to the right. Arrows indicate co‐localization.

**FIGURE 3:**
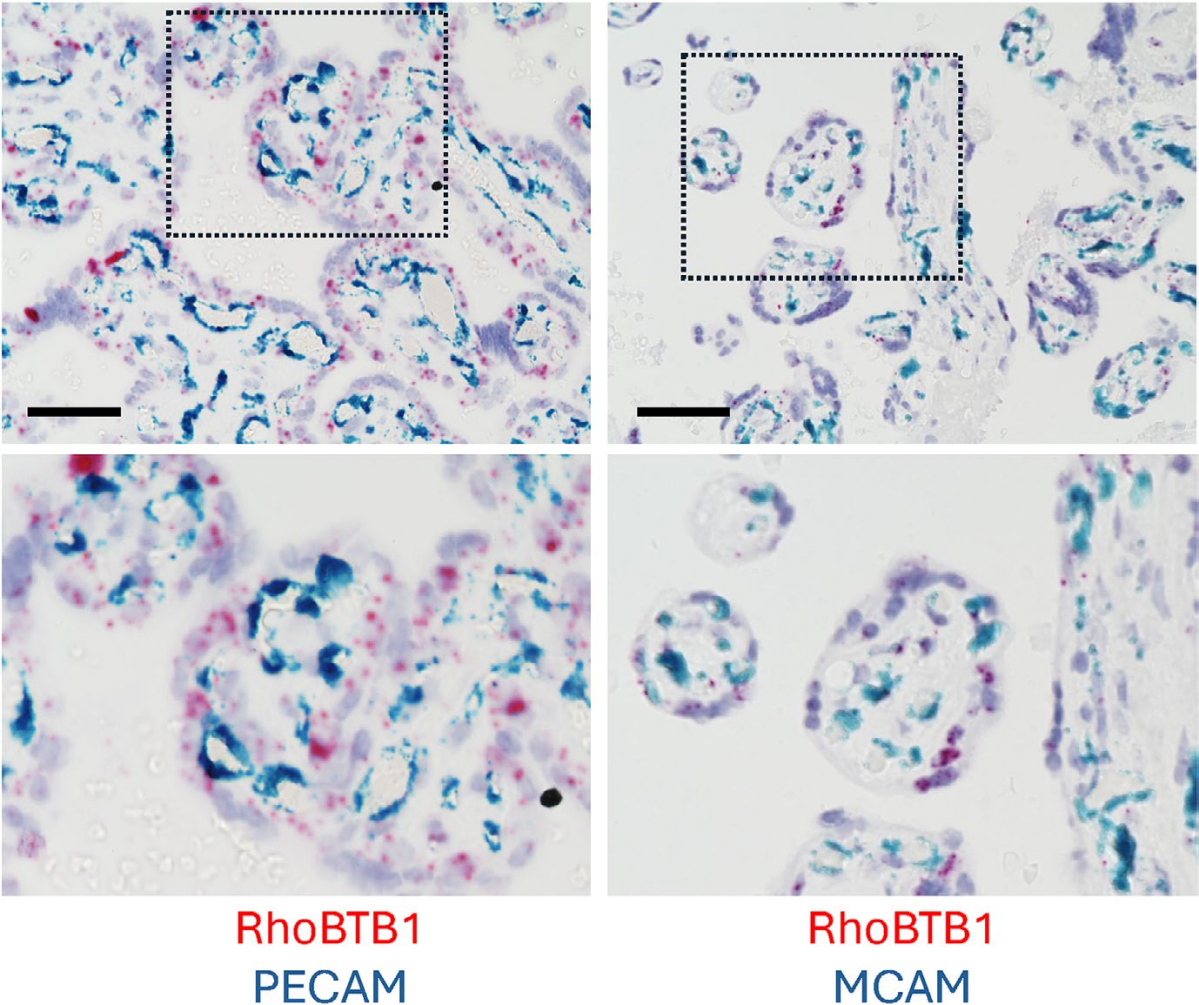
RhoBTB1 expression in endothelial cells in human placenta. Representative RNAScope in situ hybridization of human placental samples (maternal side) from preterm normal (left) and preterm preeclamptic (right) pregnancies. Expression of RhoBTB1 (red puncta) with two markers (PECAM, MCAM) of endothelial cells (blue) is shown. Scale bar is 50 μm. The region inside the dotted rectangle is expanded below.

**FIGURE 4:**
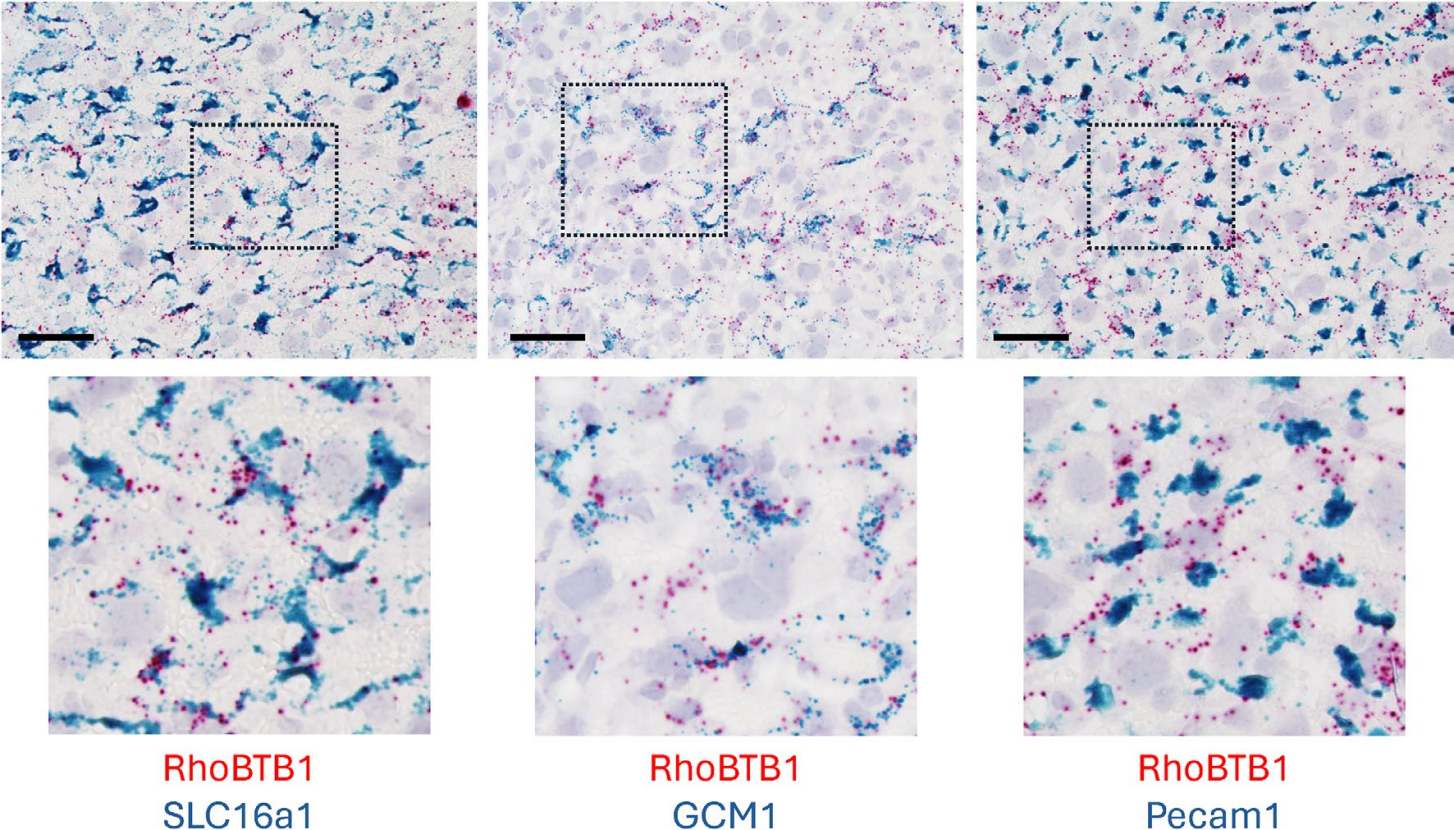
Expression of RhoBTB1 in mouse placenta. Representative RNAScope in situ hybridization of a mouse 17.5 gestation day placenta. Expression of RhoBTB1 (red puncta), markers of SCT cell layer I (SLC16a1, blue puncta) and syncytiotrophoblast cell layer II (Gcm1 markers, blue puncta) are shown. Scale bar is 50 μm. The region inside the dotted rectangle is expanded below.

One of the tissues with the highest level of RhoBTB1 expression is the placenta (Figure 1a). RNAScope in situ hybridization was performed on the maternal side from a total of 9 human placental samples. By virtue of its co‐localization with the SCT marker CYP19A1, we determined that *RhoBTB1* was expressed in the SCT layer of the term‐delivered human placenta (*n* = 3) (Figure 2). This finding was then replicated in duplicate from placentas delivered pre‐term. Given that *RhoBTB1* is a PPARγ target gene, and serum from preeclamptic pregnancies had reduced levels of PPARγ ligands even before the onset of clinical features of preeclampsia, we also examined the localization of *RhoBTB1* in placentas from preeclamptic pregnancies (Waite et al., 2005). Like normal placenta, *RhoBTB1* expression was localized to SCT cells in both preeclamptic pre‐term and term placenta (*n* = 2 each). The localization of RhoBTB1 in SCT cells is consistent with freely available single cell sequencing data. There was scant evidence that *RhoBTB1* co‐localized with the endothelial markers *PECAM* or *MCAM* in human placenta, although their localization in neighboring cells was often observed (Figure 3). Interestingly, single cell sequencing data suggests that *RhoBTB1* is expressed in vascular endothelium. In situ hybridization was also used to determine types of cells that express *RhoBTB1* in the mouse placenta from gestation day 14.5 (data not shown) and 17.5 (Figure 4). Due to its co‐localization with *Slc16a1*, *Gcm1*, and *Pecam1*, we can conclude that *RhoBTB1* is similarly expressed in SCT layers I and II in the mouse placenta. Unlike human placenta, *RhoBTB1* was also expressed in some endothelial cells.

**FIGURE 5:**
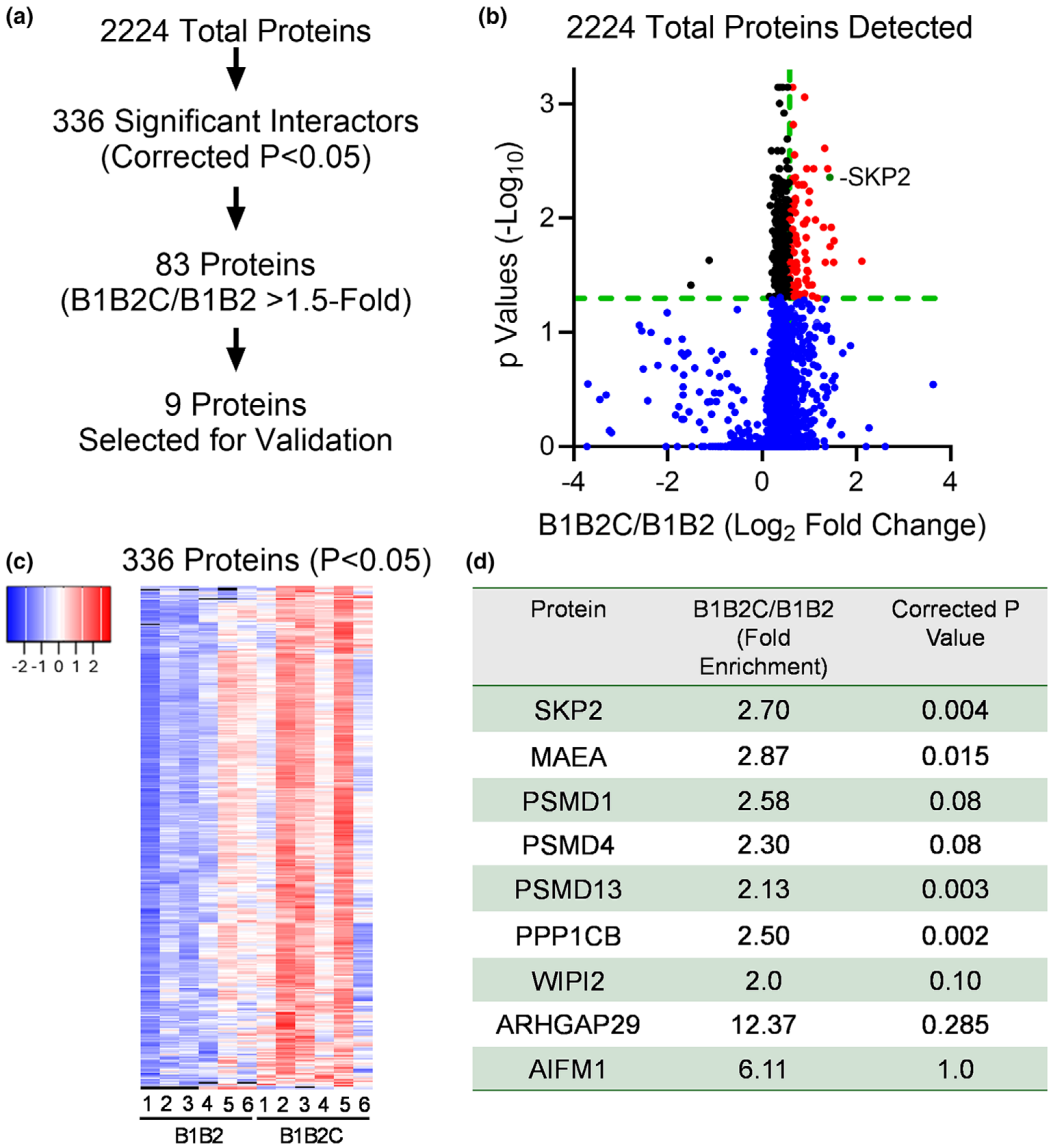
Profiling of the RhoBTB1 HTR8 proteome. (a) Schematic of the selection of proteins to examine mechanistically. (b) Volcano plot of proteomic data. The magnitude of change (Log2 fold change) is plotted on the x‐axis against the statistical significance (−Log10) on the y‐axis. Proteins highlighted in red represent higher interaction towards B1B2C. SKP2 is highlighted in green. Proteins highlighted in blue represent those below statistical significance. (c) Heatmap and hierarchical clustering of significantly interacting HTR8 differential proteome identified by mass spectrometry. (*N* = 3 technical of 2 biological replicates each) per group. (d) Nine proteins selected by applying a cut‐off interaction of B1B2C/B1B2 >1.5‐fold and statistical significance (*p* < 0.05 adjusted by Benjamini‐Hochberg procedure) or identified as potentially highly expressed in placenta.

We next employed the ascorbate peroxidase 2 (APEX2) proximity labeling to identify RhoBTB1 target proteins. RhoBTB1 consists of five distinct domains: (1) a GTPase domain, (2) a proline‐rich domain, (3) two BTB domains, and (4) a C‐terminal domain. We previously showed that the two BTB and C‐terminal domains (termed B1B2C) are necessary to interact with RhoBTB1 targets and that the GTPase and proline‐rich domains are dispensable for mediating its function as a CUL3 adaptor (Kumar et al., 2023). We employed a B1B2C‐APEX2 fusion protein as bait to capture RhoBTB1 binding partners in HTR8/SVneo (HTR8) cells. HTR8 cells are a SV40 T antigen immortalized cell line originally derived from placental trophoblasts (Graham et al., 1993). We identified a total of 2224 potentially interacting proteins by mass spectrometry, 336 of which were considered significant interactors as they exhibited a corrected *p* value of <0.05 across the samples (Figure 5a–c and Extended Dataset S1). In order to further enrich for RhoBTB1 binding proteins, we compared the mass spectrometry data with another set of samples derived from a fusion protein termed B1B2‐APEX2 which lacked the C‐terminal domain of RhoBTB1. 83 proteins were at least 1.5‐fold more abundant in B1B2C‐APEX2 compared with B1B2‐APEX2 and were also significantly significant (Table S3). We first chose 4 of those proteins (SKP2, MAEA, PSMD13, PPP1CB) because they exhibited higher B1B2C vs. B1B2 enrichment, had corrected *p* values less than 0.05, and exhibited evidence of expression in the placenta including in SCT cells according to the Human Protein Atlas (Figure 5d) (Gao et al., 2025). An additional 5 proteins were chosen because they exhibited high (PSMD1, PSMD4, WIPI2) or very high (ArhGAP29, AIFM) B1B2C vs. B1B2 enrichment (even though they were not statistically significant) and exhibited evidence of high expression in the placenta. Interestingly, 8 of the 9 proteins (all except AIFM) exhibited an enrichment of close to 1.0 (range 0.8–1.4) when we examined the mass spectroscopy signals comparing B1B2C with a mutant of B1B2C (B1B2C^P353A^) which prevents the binding of RhoBTB1 to CUL3 but preserves the binding of substrates to RhoBTB1 (Table S4) (Kumar et al., 2023). Thus, at least 8 of the selected proteins bound to RhoBTB1 in a CUL3‐independent manner consistent with the role of RhoBTB1 as a substrate adaptor. In contrast, the mass spectroscopy data suggest the binding of AIFM to RhoBTB1 may be CUL3‐dependent.

Many of these proteins play a role in the ubiquitin proteasomal pathway. SKP2 was immediately considered a high value candidate because it encodes a member of the F‐box family of proteins which serves as a subunit of another ubiquitin ligase called SCF (SKP1‐cullin‐F‐box) (Nakayama & Nakayama, 2005). Moreover, SKP2 exhibits a pattern of tissue‐specific expression which resembles RhoBTB1, that is, its highest level of expression is the placenta (Figure 1b).

**FIGURE 6:**
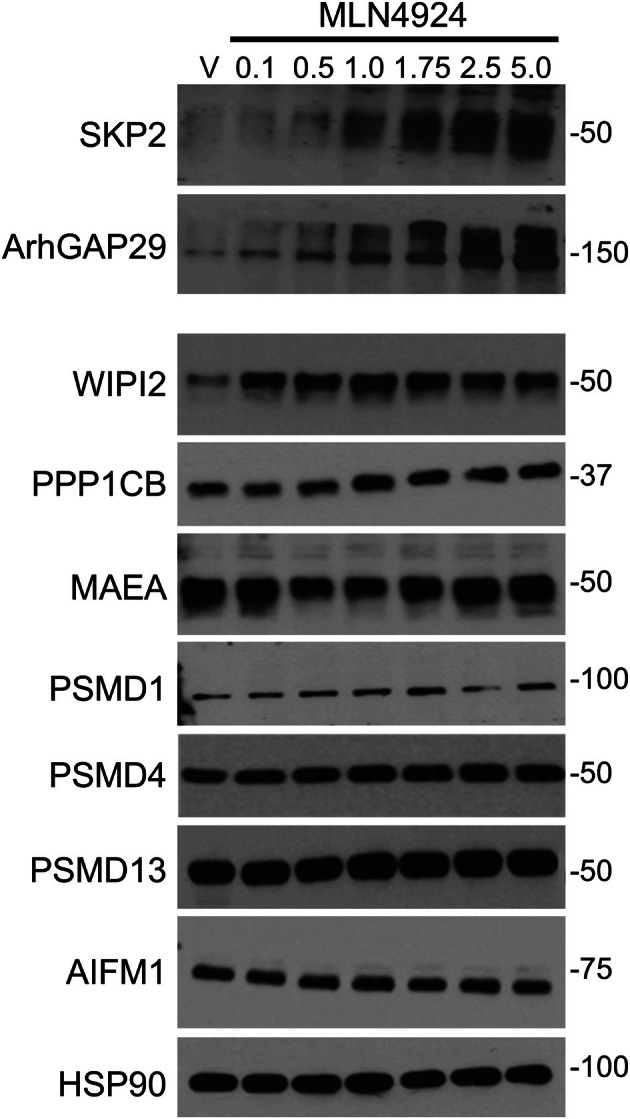
Expression of putative RhoBTB1 targets in response to cullin blockade. Immunoblot of the indicated proteins in HTR8 cells treated with increasing doses of MLN4924 (μM). HSP90 was used as a protein loading control. The SKP2 and ArhGAP29 experiments were replicated independently. Actual size markers were transferred from the original blots.

We next asked if each of the RhoBTB1 target proteins was regulated by CUL3. To accomplish this, HTR8 cells were treated with MLN4924, an inhibitor of neddylation (Pelham et al., 2012). Neddylation of Cullin proteins is required for its activity, and thus MLN4924 acts as a pan‐Cullin inhibitor. We expected that inhibiting Cullin proteins, in particular CUL3, would lead to an increase in abundance of the targets due to inhibition of ubiquitination and proteasomal degradation. Indeed, expression of SKP2 and ArhGAP29 markedly increased in response to MLN4924, suggesting they are likely degraded in a Cullin‐dependent manner (Figure 6). The SKP2 and ArhGAP29 data were replicated in separate experiments (data not shown). There was no clear evidence for increased abundance of the other seven candidate targets. Because APEX2‐mediated labeling permits the labeling of proteins in close proximity but not in direct contact to the bait suggests they may represent false positive associations with RhoBTB1, associations with other BTB‐domain containing proteins, or may just be in close proximity to RhoBTB1 in the cell. It is also possible that they are RhoBTB1 binding proteins which are not regulated by the Cullin family. Based on this, we focused the remainder of the studies on SKP2 and ArhGAP29.

**FIGURE 7:**
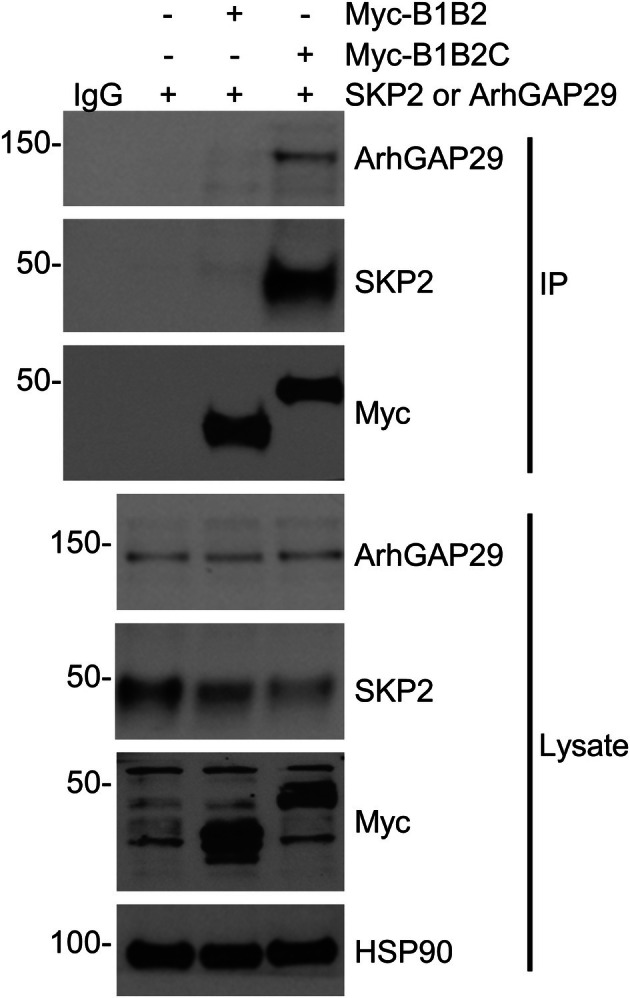
Direct interaction between RhoBTB1 and ArhGAP29 and SKP2. Co‐immunoprecipitation assay was performed with myc‐tagged RhoBTB1 domains (B1B2C and B1B2) transfected in HTR8 cells using anti‐myc beads or isotype‐matched IgG as control. Cells in the control lane were transfected with pcDNA3.1 empty vector. Immunocomplexes were western blotted with indicated anti‐sera (ArhGAP29, SKP2, or Myc). IP represents immunoprecipitates, Lysate represents whole cell lysates. Molecular weight markers are translated from original blots. Data is representative of two independent experiments. HSP90 was used as an internal control for protein loading.

**FIGURE 8:**
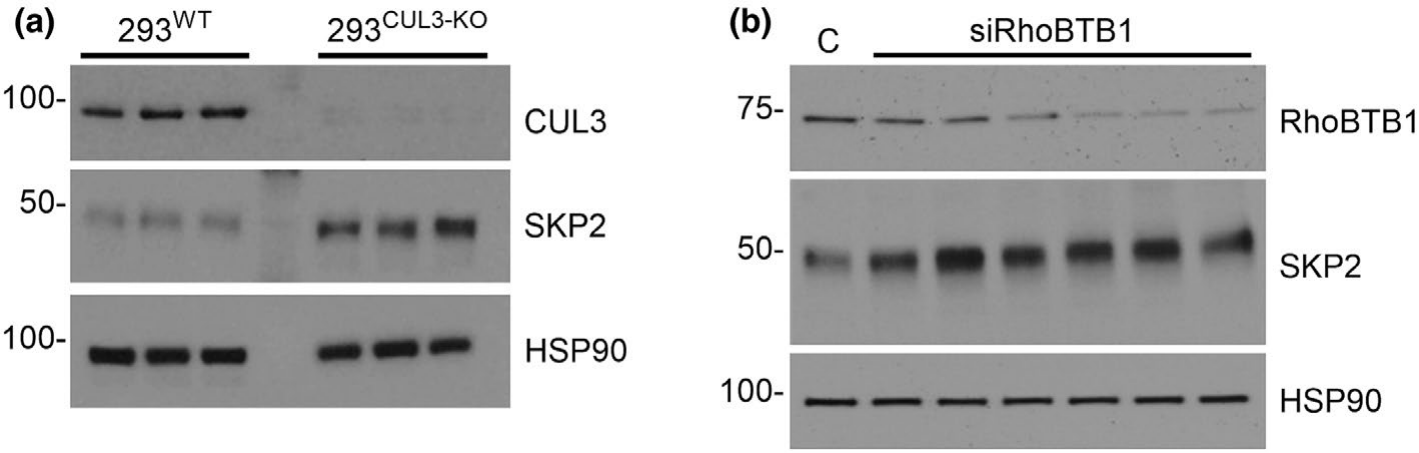
SKP2 is a RhoBTB1‐mediated CUL3 target. (a) Relative levels of CUL3 and SKP2 in HEK293^WT^ and HEK293^CUL3KO^ cells. (b) Relative levels of RhoBTB1 and SKP2 in HTR8 cells transfected with scrambled control (C) and siRNA targeting *RhoBTB1* (*siRhoBTB1*) for 72 h. HSP90 was used an internal control for protein loading. Molecular weight markers are translated from original blots.

**FIGURE 9:**
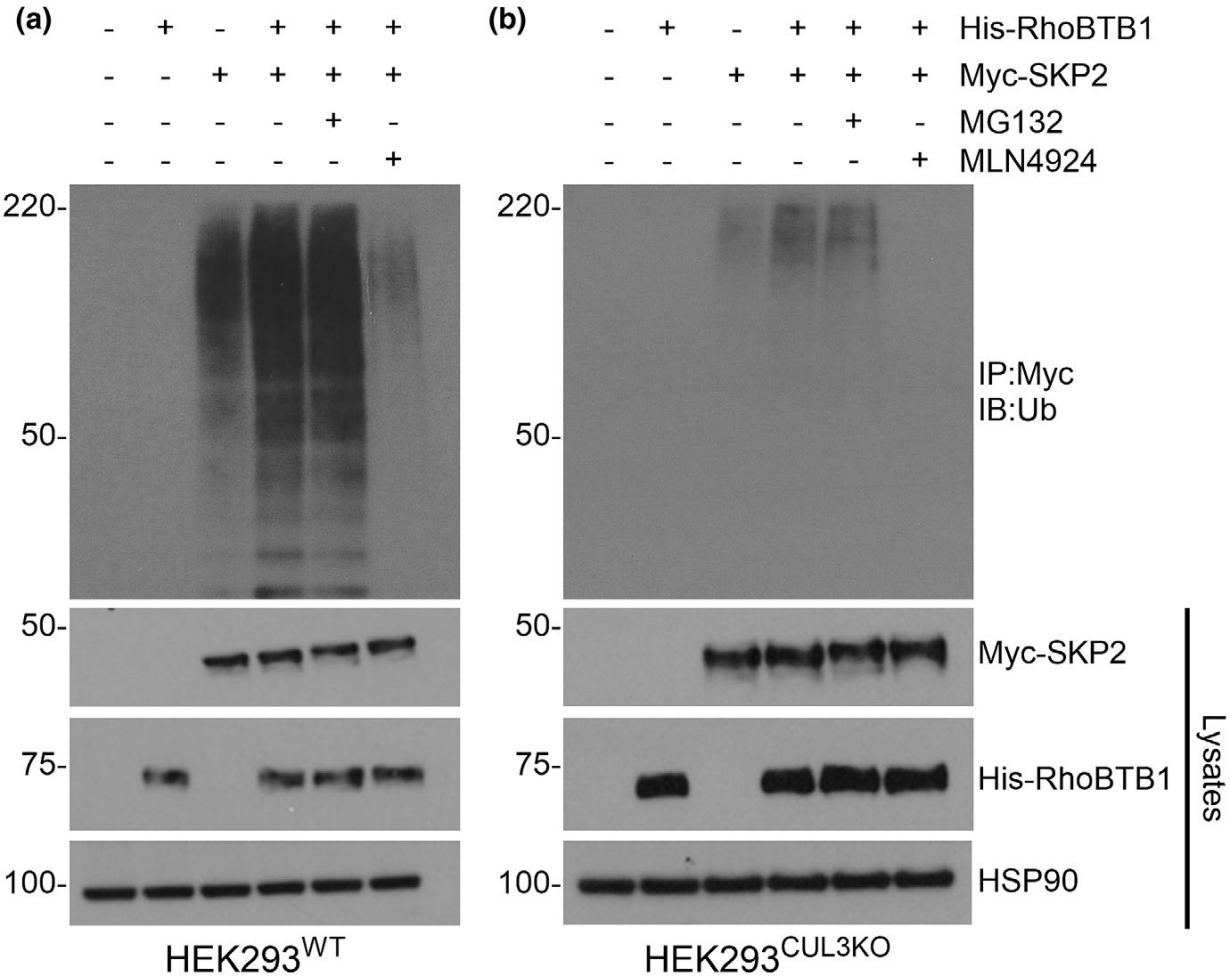
RhoBTB1‐dependent and RhoBTB1‐independent polyubiquitination of SKP2. Representative immunoblots showing polyubiquitination observed in wild‐type HEK293 cells (a, HEK293^WT^) and CRISPR/Cas9 edited CUL3 knock out HEK293 (b, HEK293^CUL3KO^) cells transfected with the indicated His‐tagged RhoBTB1, Myc‐tagged RbFox2, and ubiquitin constructs. Whole‐cell lysates were collected, and immunoprecipitation was performed under denaturing conditions that prevent RhoBTB1 from getting pulled down. Immune complexes were probed with the indicated antisera. The top blots are the IP, and the bottom three blots are from whole cell lysates. Molecular weight markers are translated from original blots. HSP90 was used as an internal control for protein loading. These data are representative of two independent experiments.

We next validated the direct protein–protein interaction of SKP2 and ArhGAP29 with RhoBTB1 by co‐immunoprecipitation. Consistent with the mass spectrometry results, both proteins were found to co‐immunoprecipitate with B1B2C but not B1B2, further validating them as RhoBTB1 binding proteins (Figure 7). Interestingly, expression of SKP2, but not ArhGAP29 (data not shown) markedly increased in abundance in CRISPR‐Cas9 generated CUL3‐deficient HEK293 cells (HEK293^CUL3KO^, Figure 8a) and in HTR8 cells treated with an siRNA targeting *RhoBTB1* (Figure 8b). This suggests that of the nine proteins selected from the mass spectrometry data, SKP2 may be the only bona fide RhoBTB1 target protein regulated by CUL3. Finally, we performed a ubiquitin assay in HEK293 and CUL3‐deficient HEK293 (HEK293^CUL3KO^). Cells were transfected with a combination of His‐tagged full‐length RhoBTB1, Myc‐tagged SKP2, and HA‐tagged ubiquitin. SKP2 was immunoprecipitated with the antisera to the Myc epitope and the Western blots were probed with an anti‐ubiquitin antibody to detect the ubiquitinated form of SKP2 (Figure 9a). A modest ubiquitinated SKP2 signal was detected in the absence of RhoBTB1 which increased in its presence suggesting SKP2 may be ubiquitinated in a RhoBTB1‐dependent and RhoBTB1‐independent manner. Because most of the signal was lost when the cells were treated with MLN4924 and in HEK293^CUL3KO^ cells, this strongly suggests that SKP2 is ubiquitinated by a CUL3‐dependent mechanism (Figure 9b).

**FIGURE 10:**
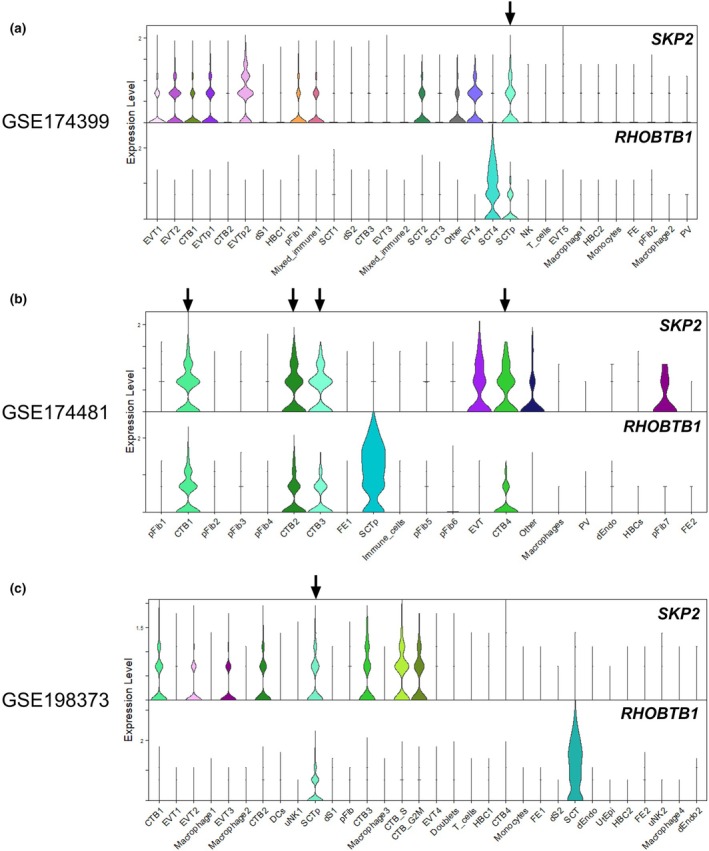
Co‐expression of RhoBTB1 and SKP2 in human placenta. Reanalysis of single cell RNA sequencing data from three freely available datasets (GSE174399 (a), GSE174481 (b), GSE198373 (c)) from the Gene Expression Omnibus (GEO). Gene expression plots show the counts per million (CPM) of SKP2 versus RhoBTB1 in individual cells and include the dataset‐wide distance correlation (dCor: Distance correlation statistic), distance correlation *p* value, and Spearman's *r* to show direction of the relationship. The downward black arrows indicate clusters co‐expressing SKP2 and RhoBTB1. CTB, cytotrophoblast; DC, dendritic cell; dEndo, decidual endothelial cell; dS, decidual stromal cell; FE, fetal endothelial cell; HBC, fetal Hofbauer cell; NK/uNK, uterine natural killer cell; pFib, placental fibroblast; PV, perivascular cell; SCT, syncytiotrophoblasts; SCTp, syncytiotrophoblast precursor cells; UtEpi, uterine epithelial cells.

We next asked if SKP2 and RhoBTB1 are co‐expressed in human placenta. To do this, we re‐examined three published single cell sequencing datasets. In the first (GSE174399) derived from control placentas from 4 patients, *SKP2* and *RhoBTB1* were co‐expressed in SCT precursor cells and SCTs (Figure 10a and Figure S1) (Zheng et al., 2022). In the second dataset (GSE174481) derived from 7 control patients, co‐expression was evident in several clusters including cytotrophoblasts and less in SCTs (Figure 10b and Figure S2) (Shannon et al., 2022). In the third dataset (GSE198373) derived from 4 control patients, co‐expression was observed in an intermediate SCT cell type and in cytotrophoblasts and less in SCT precursor cells (Figure 10c and Figure S3) (Marsh et al., 2022). These data provide evidence for the co‐expression of *RhoBTB1* and *SKP2* in the placenta in a variety of trophoblast cells. It is notable that there was generally a weak to moderate dependency and a negative correlation between expression of RhoBTB1 and SKP2 (dCor = 0.25–0.32, Spearman's *r* = −0.49 to −0.53; Figure S4). Thus, higher expression of RhoBTB1 generally correlated with lower expression of SKP2 which is largely consistent with the hypothesis that cells with higher expression of RhoBTB1 would target SKP2 for ubiquitination and ultimate degradation. It is important to note that expression of CUL3 is widely expressed in many placental cell types and thus the machinery to regulate SKP2 is present.

## 4 DISCUSSION

The main findings from this study are that RhoBTB1, an adaptor which delivers protein substrates to CUL3 for ubiquitination and proteasomal degradation, is (1) robustly expressed in the human and mouse placenta, (2) localized mainly to SCT, (3) likely binds to many placental proteins including ArhGAP29 and SKP2, and (4) binds and delivers SKP2 to the CUL3 complex for ubiquitination and proteasomal degradation. Thus, RhoBTB1 likely plays a role in the regulation of protein homeostasis in the placenta.

From RNAScope in situ hybridization, we confirmed RhoBTB1 expression in the placenta; specifically, in the SCT cell layer. RhoBTB1 might also be expressed in some placental endothelial cells which would be consistent with expression in endothelial cells in other tissues including lung, ovary, spleen, and thymus. Other major cell types expressing RhoBTB1 include the proximal tubule of the kidney and resident macrophages in a variety of tissues. Similar findings were observed in the mouse placenta. There was no qualitative change in co‐localization based on whether the placenta was obtained pre‐term or at term, or from a preeclamptic pregnancy. Of course, there may be differences in level of expression of RhoBTB1 in the placenta based on disease state, but this was not determined. We recognize that the lack of quantification of the RNAscope could be considered a weakness of the study.

We employed APEX2‐mediated proximity labeling to identify the RhoBTB1 proteome in HTR‐8 cells. HTR‐8 cells are a trophoblast cell line that endogenously express RhoBTB1 and are easy to transfect. We recognize that the use of a cell line could be considered a limitation, but it facilitated the initial APEX2‐mediated screen for RhoBTB1 binding proteins that could later be validated in primary placental cells or placental tissue. We utilized a strategy to compare the proximity labeled proteome identified by fusing the B1B2C domains of RhoBTB1 with a fusion protein lacking the C‐terminal domain (B1B2). We previously reported that the C‐terminal half of RhoBTB1 consisting of the first and second BTB domains and the C‐terminal domain is all that is required to bind RhoBTB1 substrates and to target their ubiquitination (Kumar et al., 2023). Removing the C‐terminal domain from the two upstream BTB domains eliminates those associations. Thus, comparing the B1B2C and B1B2 proteomes provides a mechanism to enrich for true RhoBTB1 binding proteins. This is necessary given that over 2200 proteins were identified by mass spectroscopy. In our first proof‐of‐principle study performed in HEK293 cells, nearly 80% of the proteins identified by this method were validated as RhoBTB1 binding proteins (Kumar et al., 2023). A lower but still impressive success rate of 50% was observed in A7R5 smooth muscle cells (Kumar et al., 2026). In this study, 83 proteins were identified by mass spectroscopy that exhibited both an enrichment for binding the B1B2C domain and were deemed statistically significant. Two of these, SKP2 and ArhGAP29, increased in abundance in response to inhibition of Cullin activity, and both of these co‐immunoprecipitated with RhoBTB1. We focused further efforts on SKP2 because like RhoBTB1, it is highly and selectively expressed in placenta, and its expression increased in HEK293 cells genetically engineered to lack CUL3 and in HTR8 cells treated with an siRNA targeting RhoBTB1. Finally, we showed that SKP2 is ubiquitinated in a CUL3‐dependent manner and utilized RhoBTB1 as the substrate adaptor. There was some ubiquitination in the absence of transfected RhoBTB1 suggesting the possibility that SKP2 might utilize another adaptor.

Interestingly, SKP2 encodes a member of the F‐box family of proteins. The F‐box domain serves as one of four subunits of another ubiquitin ligase called SCF (SKP1‐cullin‐F‐box) which plays a role in the turnover of proteins which regulate the cell cycle, signal transduction and transcription. Thus, SKP2 may regulate cell cycle proteins in a RhoBTB1‐dependent manner. Given the dynamic nature of placental development, it is tempting to speculate that SKP2 regulates a series of cell cycle proteins which may control placental growth and development. If, after additional experimentation, this model is proven true, it would implicate a complicated genetic circuit initiated by PPARγ to control the post‐translational stability of cell cycle proteins through PPARγ‐RhoBTB1‐CUL3‐SKP2 in the placenta.

### 4.1 Clinical relevance and conclusions

Previous studies on SKP2 have implicated it in poor pregnancy outcomes including early miscarriages, suggesting impaired development of the decidua, which plays a crucial role in supporting and regulating embryo implantation, placental development, and maternal‐fetal immune tolerance (Fotovati et al., 2011; Lv et al., 2021; Yamauchi et al., 2020). SKP2 has been shown to facilitate cell survival by limiting DNA damage and apoptosis triggered by oxidative stress in melanoma cells (Wang et al., 2017). Moreover, glucose transporter 1 (GLUT1), a protein whose dysregulation is implicated in preeclampsia, is a downstream target of SKP2. When human endometrial stromal cells were knocked down for SKP2, both mRNA and protein expression of GLUT1 were reduced (Lüscher et al., 2017; Yang et al., 2022). Therefore, SKP2 may have similar functions in placental development and further exploration of its role in the placental could lead to a more complete understanding of placental function as well as its potential role in preeclampsia.

RhoBTB1‐expressing SCT cells are in direct contact with the maternal blood in the intervillous space and represent a multinucleated structure with microvilli. Not only does the SCT cell function as a barrier, but it also is a major endocrine organ secreting multiple hormones and proteins into the maternal circulation (Turco & Moffett, 2019). Changes in the SCT layer in placentas from pregnancies compromised by hypertension and preeclampsia include oxidative, inflammatory, mitochondrial, or immune stress (Cindrova‐Davies et al., 2018; Fisher, 2015; Huppertz, 2008; Huppertz, 2011; Redman & Staff AC, 2015). In response to oxidative stress, the SCT releases inflammatory cytokines, anti‐angiogenic agents, cell‐free fetal DNA, and exosomes into the maternal circulation (Burton et al., 2019). It is believed that the maternal response to these elements leads to the clinical findings of preeclampsia, a life‐threatening disorder of pregnancy complicating about 5%–10% of all pregnancies (Wang et al., 2021). The risks preeclampsia poses to the mother and the fetus can result in devastating outcomes including seizures, heart failure, long‐term cardiovascular complications, preterm birth, hemorrhage, and death (Obstetricians ACo & Gynecologists, 2020). Women who have suffered from preeclampsia may be at higher risk for cardiovascular events in the future (Ahmed et al., 2014; Amaral et al., 2015; Bushnell et al., 2014; Charlton et al., 2014; Lazdam et al., 2012). It will be interesting to assess in future studies if RhoBTB1 is mechanistically involved in preeclampsia. It is notable that PPARγ is thought to play a role in preeclampsia and RhoBTB1 is a PPARγ target (Fang et al., 2021; Pelham et al., 2012).

## AUTHOR CONTRIBUTIONS

**Alina L. Tvina:** Conceptualization; data curation; formal analysis; investigation; methodology. **M. Christine Livergood:** Conceptualization; data curation; formal analysis; investigation; methodology. **Gaurav Kumar:** Conceptualization; data curation; formal analysis; investigation; methodology. **Valerie W. Hole:** Conceptualization; data curation; formal analysis. **Megan A. Opichka:** Data curation; investigation; methodology. **Luisangeli Muniz Irizarry:** Investigation; methodology. **Ko‐Ting Lu:** Data curation; investigation; methodology. **Kelsey K. Wackman:** Data curation. **Justin L. Grobe:** Formal analysis; project administration; supervision. **Pablo Nakagawa:** Formal analysis; investigation; methodology; project administration; supervision. **Jennifer J. McIntosh:** Conceptualization; data curation; formal analysis; investigation; methodology; project administration; supervision. **Curt D. Sigmund:** Conceptualization; formal analysis; funding acquisition; investigation; methodology; project administration; supervision.

## FUNDING INFORMATION

This study was funded by the National Institutes of Health (HL084207, HL144807, HL177123 to C.D.S., HL1509340 and TR001436 to J.J.M., HL134850 and DK133121 to J.L.G., HL153101 to P.N.), the Advancing a Healthier Wisconsin Endowment (AHW5520631 to C.D.S.; AHW5520747 to G.K., AHW5520639 to P.N.), the American Heart Association (CDA1048244 to P.N.), and grants from MCW Cardiovascular Research Center to G.K. and P.N.

## CONFLICT OF INTEREST STATEMENT

The authors have declared that no conflict of interest exists.

## Supporting information


Data S1.

Data S2.

**Figure S1:** Seurat identifies 31 cell clusters from four human placenta scRNAseq datasets from GSE174399. Matrix files from D1, D8, D9, and D13 were downloaded from GEO and reanalyzed in R using the Seurat package. (a) Uniform manifold approximation and projection (UMAP) plot of cell clusters and their identities. (b) Clusters split by patient. (c) UMAP plots of marker genes for unique cell types and trophoblast identities. (d) Heat map of marker genes used to identify different cell types. (e) Percent of clusters that express neither SKP2 or RhoBTB1 (SKP2− RhoBTB1−), that express only SKP2 (SKP2+ RhoBTB1−), that express only RhoBTB1 (SKP2− RhoBTB1+), and that co‐express SKP2 and RhoBTB1 (SKP2+ RhoBTB1+). CTB, cytotrophoblast; dS, decidual stromal cell; FE, fetal endothelial cell; HBC, fetal Hofbauer cell; NK, uterine natural killer cell; pFib, placental fibroblast; PV, perivascular cell; SCT, syncytiotrophoblasts; SCTp, syncytiotrophoblast precursor cells.
**Figure S2:** Seurat identifies 21 cell clusters from seven human placenta scRNAseq datasets from GSE174481. Matrix files from P21, P89, P130, P244, P277, P295, P899 were downloaded from GEO and reanalyzed in R using the Seurat package. (a) Uniform manifold approximation and projection (UMAP) plot of cell clusters and their identities. (b) Clusters split by sex and the gestational age (GA) of the fetus. (c) UMAP plots of marker genes for unique cell types and trophoblast identities. (d) Heat map of marker genes used to identify different cell types. (e) Percent of clusters that express neither SKP2 or RhoBTB1 (SKP2− RhoBTB1−), that express only SKP2 (SKP2+ RhoBTB1−), that express only RhoBTB1 (SKP2− RhoBTB1+), and that co express SKP2 and RhoBTB1 (SKP2+ RhoBTB1+). CTB, cytotrophoblast; dEndo, decidual endothelial cell; FE, fetal endothelial cell; HBC, fetal Hofbauer cell; pFib, placental fibroblast; SCT, syncytiotrophoblasts; SCTp, syncytiotrophoblast precursor cells.
**Figure S3:** Seurat identifies 32 cell clusters from four human placenta scRNAseq datasets from GSE198373. Matrix files from 17_SC, 17_VC, 18_SC, 18_VC, 23_SC, 23_VC, 24_SC, 24_VC were downloaded from GEO and reanalyzed in R using the Seurat package. (a) Uniform manifold approximation and projection (UMAP) plot of cell clusters and their identities. (b) Clusters split by tissue type. (c) UMAP plots of marker genes for unique cell types and trophoblast identities. (d) Heat map of marker genes used to identify different cell types. (e) Percent of clusters that express neither SKP2 or RhoBTB1 (SKP2− RhoBTB1−), that express only SKP2 (SKP2+ RhoBTB1−), that express only RhoBTB1 (SKP2− RhoBTB1+), and that co‐express SKP2 and RhoBTB1 (SKP2+ RhoBTB1+). CTB, cytotrophoblast; DC, dendritic cell; dEndo, decidual endothelial cell; dS, decidual stromal cell; FE, fetal endothelial cell; HBC, fetal Hofbauer cell; uNK, uterine natural killer cell; pFib, placental fibroblast; SCT, syncytiotrophoblasts; SCTp, syncytiotrophoblast precursor cells; UtEpi, uterine epithelial cells.
**Figure S4:** Correlation of RhoBTB1 and SKP2 Expression. Gene expression plots show the counts per million (CPM) of SKP2 versus RhoBTB1 in individual cells and include the dataset wide distance correlation (dCor: distance correlation statistic), distance correlation *p* value, and Spearman's *r* to show direction of the relationship.
**Table S1:** Chromatography and MS instrument acquisition settings.
**Table S2:** Mass spectrometry data processing parameters.
**Table S3:** HTR8 RhoBTB1 proteome (B1B2C/B1B2 abundance ratio >1.5‐fold; adjusted *p* < 0.05).
**Table S4:** B1B2C/B1B2 abundance vs. B1B2CP353A/B1B2C ratio.

## Data Availability

A file with raw blots is provided as supplemental material. Mass spectrometry proteomics data has been deposited to the Proteome Xchange Consortium via the PRIDE partner repository with the dataset identifier PXD073804 and 10.6019/PXD073804. The data underlying this article will be shared upon reasonable request to the corresponding author and will be available on the Harvard Dataverse.
