# Supplementary material for: Evidence that S‐phase kinase associated protein 2 (SKP2) is ubiquitinated and degraded via a Rho‐related BTB domain containing 1 (RhoBTB1) and Cullin‐3 mechanism in placenta
Source: Physiol Rep. 2026 Aug 4;14(15):e71039. doi: 10.14814/phy2.71039 (PMC13437954; doi:10.14814/phy2.71039)
Supplement: Supplementary file 1 [file PHY2-14-e71039-s001.pdf]

# Supplemental Figures and Tables

Evidence that S-Phase Kinase Associated Protein 2 (SKP2) is Ubiquitinated and Degraded via a Rho-related BTB Domain Containing 1 (RhoBTB1) and Cullin-3 Mechanism in Placenta

Alina L. Tvina[1*], M. Christine Livergood[1*], Gaurav Kumar[2], Valerie W. Hole[2], Megan A. Opichka, Luisangeli Muniz Irizarry[2], Ko-Ting Lu[2], Kelsey K. Wackman[2], Justin L. Grobe[2], Pablo Nakagawa[2], Jennifer J. McIntosh[1] and Curt D. Sigmund[2]

*, Denotes Equal Contribution

[1]Department of Obstetrics and Gynecology, Cardiovascular Center

[2]Department of Physiology, Cardiovascular Center

Medical College of Wisconsin, Milwaukee, WI, USA, 53226

Short Title: SKP2 is a RhoBTB1-CUL3 Target in Placenta

Conflict-of-interest statement: The authors have declared that no conflict of interest exists.

*Corresponding Author:
Curt D. Sigmund, Ph.D.
Department of Physiology
Medical College of Wisconsin
8701 Watertown Plank Road
Milwaukee, WI 53226-0509
Phone: 414-955-8277
E-mail: csigmund@mcw.edu

**Supplementary Figures and Legends**

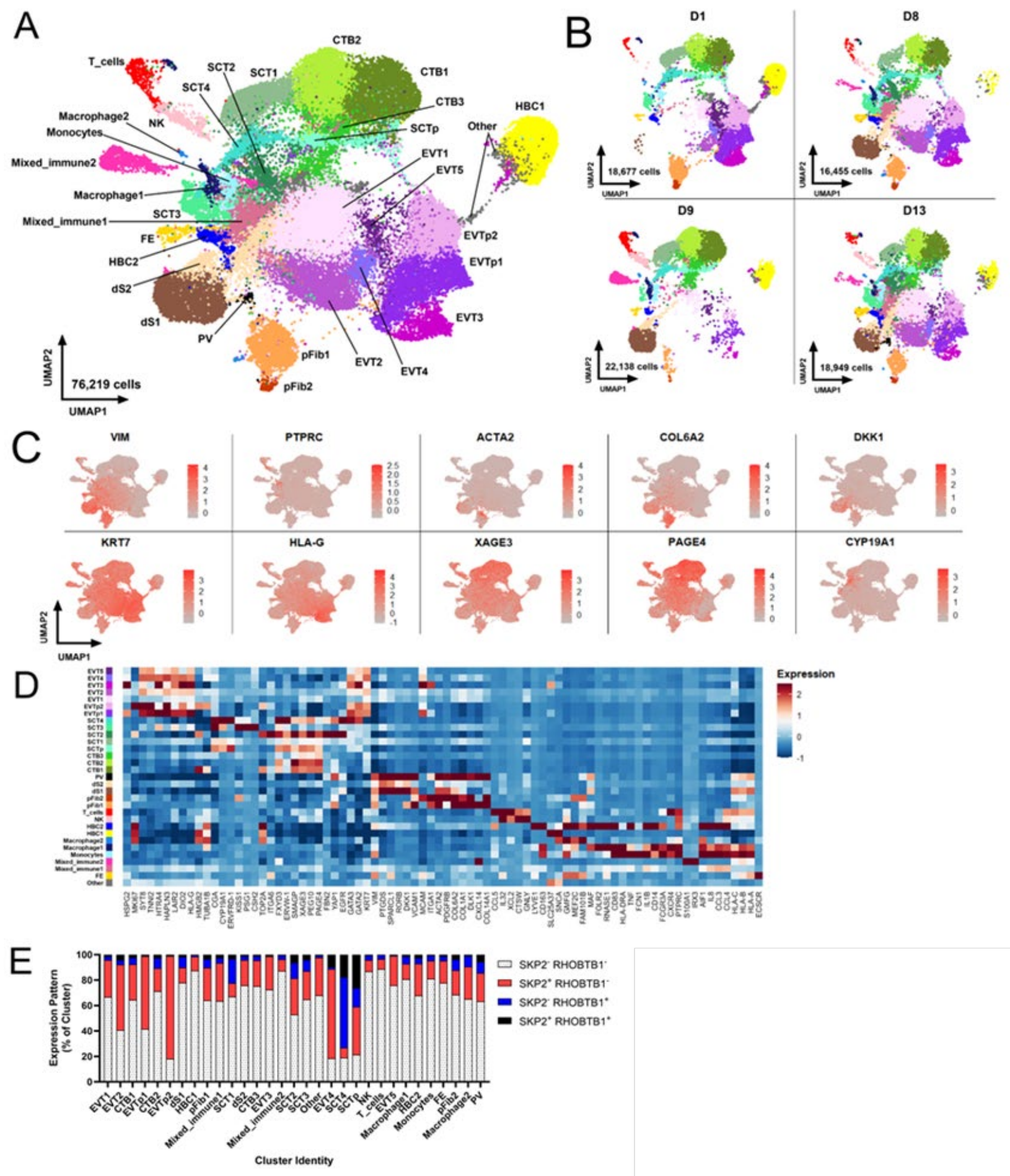

**Figure S1.** *Seurat identifies 31 cell clusters from four human placenta scRNAseq datasets from GSE174399.* Matrix files from D1, D8, D9, and D13 were downloaded from GEO and reanalyzed in R using the Seurat package. (A) Uniform manifold approximation and projection (UMAP) plot of cell clusters and their identities. (B) Clusters split by patient. (C) UMAP plots of marker genes for unique cell types and trophoblast identities. (D) Heat map of marker genes used to identify different cell types. (E) Percent of clusters that express neither SKP2 or RHOBTB1 (SKP2- RHOBTB1-), that express only SKP2 (SKP2+ RHOBTB1-), that express only RHOBTB1 (SKP2- RHOBTB1+, and that co-express SKP2 and RHOBTB1 (SKP2+ RHOBTB1+). CTB, cytotrophoblast; dS, decidual stromal cell; FE, fetal endothelial cell; HBC, fetal Hofbauer cell; NK, uterine natural killer cell; pFib, placental fibroblast; PV, perivascular cell; SCT, syncytiotrophoblasts; SCTp, syncytiotrophoblast precursor cells.

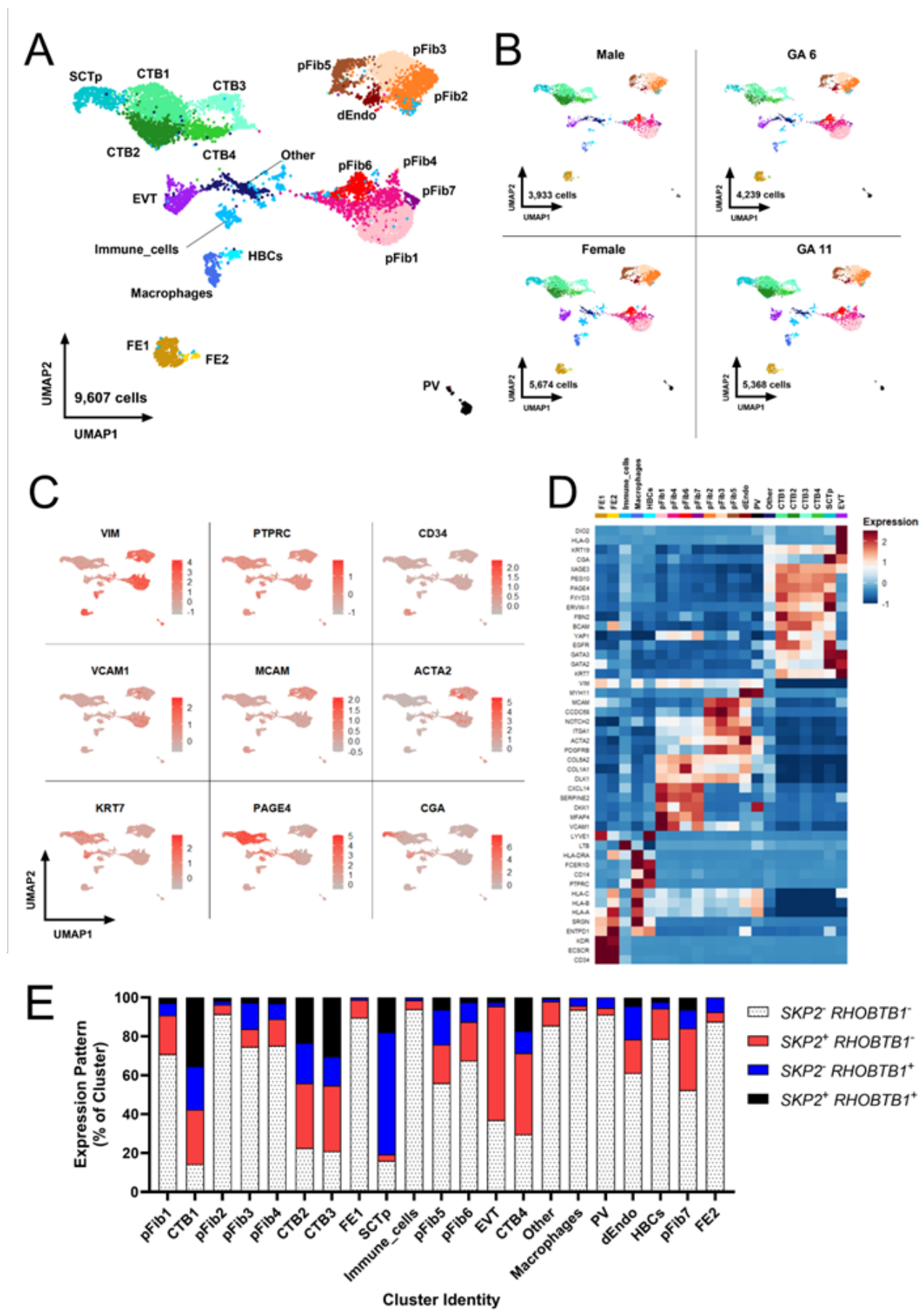

**Figure S2.** *Seurat identifies 21 cell clusters from seven human placenta scRNAseq datasets from GSE174481. Matrix files from P21, P89, P130, P244, P277, P295, P899 were downloaded from GEO and reanalyzed in R using the Seurat package.* (A) Uniform manifold approximation and projection (UMAP) plot of cell clusters and their identities. (B) Clusters split by sex and the gestational age (GA) of the fetus. (C) UMAP plots of marker genes for unique cell types and trophoblast identities. (D) Heat map of marker genes used to identify different cell types. (E) Percent of clusters that express neither SKP2 or RHOBTB1 (SKP2- RHOBTB1-), that express only SKP2 (SKP2+ RHOBTB1-), that express only RHOBTB1 (SKP2- RHOBTB1+, and that co-express SKP2 and RHOBTB1 (SKP2+ RHOBTB1+). CTB, cytotrophoblast; dEndo, decidual endothelial cell; FE, fetal endothelial cell; HBC, fetal Hofbauer cell; pFib, placental fibroblast; SCT, syncytiotrophoblasts; SCTp, syncytiotrophoblast precursor cells.

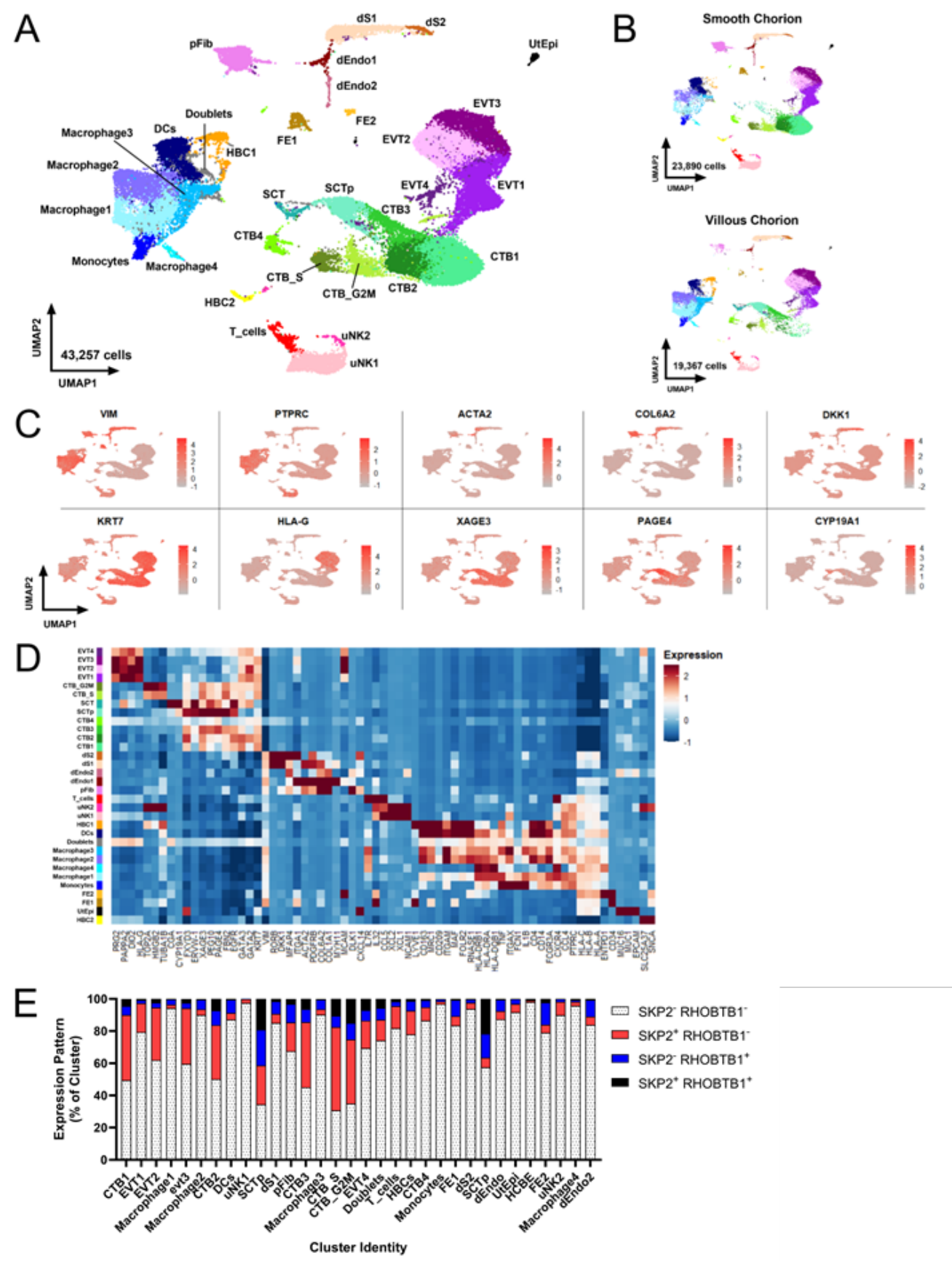

**Figure S3.** *Seurat identifies 32 cell clusters from four human placenta scRNAseq datasets from GSE198373. Matrix files from 17_SC, 17_VC, 18_SC, 18_VC, 23_SC, 23_VC, 24_SC, 24_VC were downloaded from GEO and reanalyzed in R using the Seurat package.* (A) Uniform manifold approximation and projection (UMAP) plot of cell clusters and their identities. (B) Clusters split by tissue type. (C) UMAP plots of marker genes for unique cell types and trophoblast identities. (D) Heat map of marker genes used to identify different cell types. (E) Percent of clusters that express neither SKP2 or RHOBTB1 (SKP2- RHOBTB1-), that express only SKP2 (SKP2+ RHOBTB1-), that express only RHOBTB1 (SKP2- RHOBTB1+, and that co-express SKP2 and RHOBTB1 (SKP2+ RHOBTB1+). CTB, cytotrophoblast; DC, dendritic cell; dEndo, decidual endothelial cell; dS, decidual stromal cell; FE, fetal endothelial cell; HBC, fetal Hofbauer cell; uNK, uterine natural killer cell; pFib, placental fibroblast; SCT, syncytiotrophoblasts; SCTp, syncytiotrophoblast precursor cells; UtEpi, uterine epithelial cells.

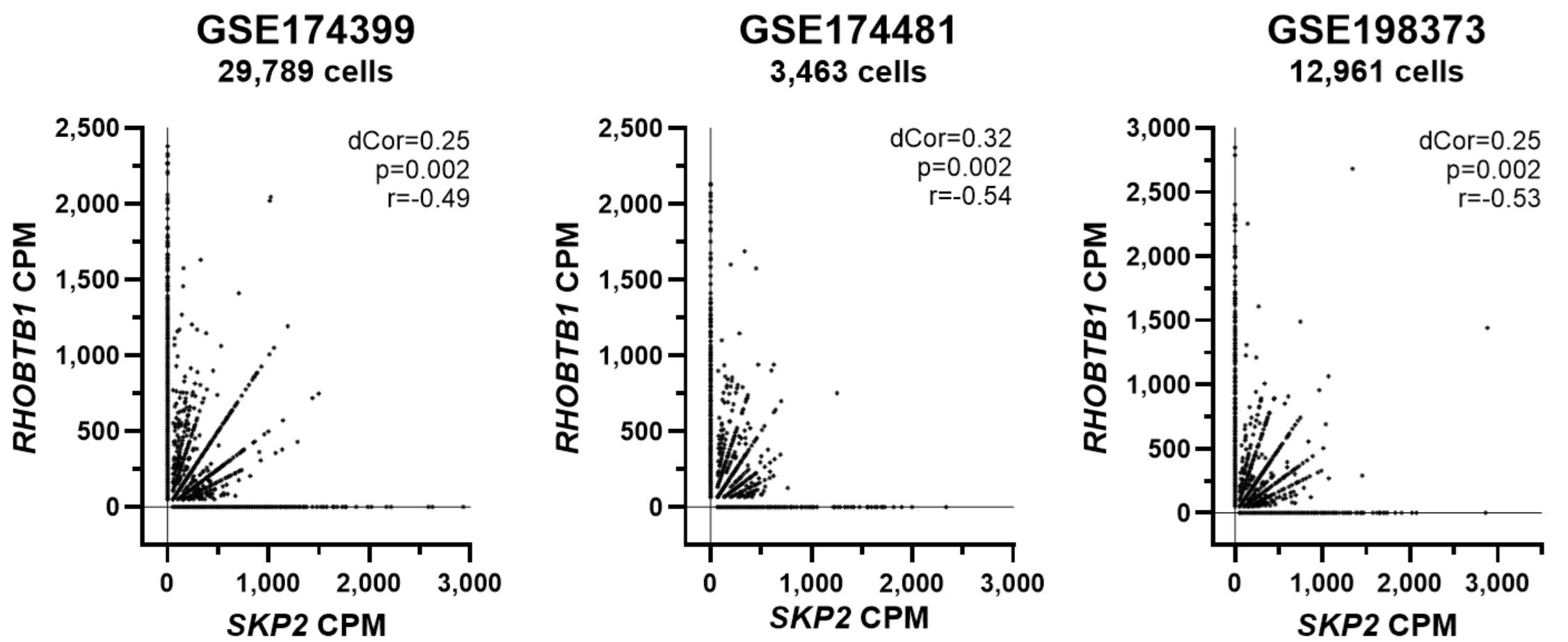


**Figure S4.** *Correlation of RhoBTB1 and SKP2 Expression*. Gene expression plots show the counts per million (CPM) of SKP2 versus RHOBTB1 in individual cells and include the dataset-wide distance correlation (dCor: distance correlation statistic), distance correlation p value, and Spearman's r to show direction of the relationship.

Table S1. Chromatography and MS Instrument Acquisition Settings.

| | | | |
|---|---|---|---|
| Sample Volume | 20 µL | Isolation Window | 1.6 m/z |
| Stationary Phase | Thermo Acclaim PepMap C18 75µm × 50cm | MS2 AGC Target | 5e4 |
| LC Solvent A | 100% $H_2O$, 0.1% formic acid | MS2 Maximum IT | 54 ms |
| LC Solvent B | 80% acetonitrile, 0.1% formic acid | Normalized Collision Energy | 30 |
| Gradient Ramp and Duration Flow Rate | 2.5-5% B in 1 minute<br>5-7% B in 4 minutes<br>7-28% B in 72 minutes<br>28-60%B in 10 minutes<br>60-99%B in 4 minutes<br>300 nL/min | Minimum Intensity Req. | 5e5 |
| Mass Spectrometer | Thermo Orbitrap Fusion Lumos | Dynamic Exclusion | 60.0 s |
| Spray Voltage | 2.1 kV | MS2 acquisition | Data dependent, 3 s cycle time, Centroid |
| In-Source CID | 0.0 eV | MS2 Fragmentation | HCD |
| MS1 scan range | 375-1500 m/z | MS2 Detection | Orbitrap |
| MS1 resolution | 120,000 @ 200 m/z | MS2 fixed first mass | 110 m/z |
| MS1 AGC Target | 4e5 | MS2 resolution | 30,000 @ 200 m/z |
| MS1 Maximum IT | 50 ms | Advanced Precursor Determination | on |

Table S2. Mass Spectrometry Data Processing Parameters

| | | | |
|---|---|---|---|
| Platform | ProteomeDiscoverer 2.4 | Quantitation | Precursor Ions Quantifier, Peak Area |
| Search Algorithms | SequestHT | Normalization | Total Peptide Amount |
| Validation | Percolator | Scaling | Validation |
| Database | Uniprot Rattus norvegicus with isoforms, 2022-04-30, MaxQuant Contaminants Recombinant sequence | Ratio Calculation | Summed Abundance Based |
| Digest | Trypsin (semi) 2 Missed Cleavages Allowed | Hypothesis Test | ANOVA (Individual Proteins) |
| Precursor mass tolerance | 10 ppm | Target FDR (Strict) for PSMs: | 0.01 |
| Fragment mass tolerance | 0.02 Da | Target FDR (Relaxed) for PSMs: | 0.05 |
| Static Modifications | Carbamidomethyl (C) | Target FDR (Strict) for Peptides: | 0.01 |
| Dynamic Modifications | Oxidation (M), Acetylation (protein N-terminus) | Target FDR (Relaxed) for Peptides: | 0.05 |

Supplemental Table 3: HTR8 RhoBTB1 Proteome
(B1B2C/B1B2 Abundance Ratio > 1.5-Fold; Adjusted P<0.05)

| Accession Number | Protein Name | B1B2C/B1B2 | Adjusted P Value |
|---|---|---|---|
| Q13404 | UBE2V1 | 4.334 | 0.023779 |
| Q7L5Y9 | MAEA | 2.873 | 0.01573 |
| P61081 | UBE2M | 2.867 | 0.024406 |
| O43294 | TGFB1I1 | 2.767 | 0.012057 |
| O94822 | LTN1 | 2.719 | 0.017726 |
| Q13309 | SKP2 | 2.706 | 0.00439 |
| Q14185 | DOCK1 | 2.62 | 0.00368 |
| Q9Y5Y2 | NUBP2 | 2.537 | 0.024406 |
| P62140 | PPP1CB | 2.508 | 0.002437 |
| Q9NQW6 | ANLN | 2.472 | 0.011999 |
| O75683 | SURF6 | 2.248 | 0.049909 |
| P11169 | SLC2A3 | 2.191 | 0.010363 |
| Q9UNM6 | PSMD13 | 2.133 | 0.00368 |
| Q9BVS4 | RIOK2 | 2.095 | 0.047943 |
| Q12788 | TBL3 | 2.088 | 0.038216 |
| O15013 | ARHGEF10 | 2.006 | 0.005796 |
| Q9BZL6 | PRKD2 | 1.984 | 0.007268 |
| Q6PGP7 | TTC37 | 1.981 | 0.038576 |
| Q9Y4X5 | ARIH1 | 1.965 | 0.029319 |
| Q8ND04 | SMG8 | 1.932 | 0.028701 |
| Q9UNZ2 | NSFL1C | 1.931 | 0.00368 |
| P02668 | CSN3 | 1.928 | 0.010363 |
| P54652 | HSPA2 | 1.916 | 0.022755 |
| P51003 | PAPOLA | 1.913 | 0.033668 |
| Q9H6S3 | EPS8L2 | 1.908 | 0.014806 |
| Q14738 | PPP2R5D | 1.9 | 0.034911 |
| Q9UID3 | VPS51 | 1.899 | 0.023067 |
| O15260 | SURF4 | 1.878 | 0.019913 |
| Q7Z3U7 | MON2 | 1.871 | 0.011175 |
| P51610 | HCFC1 | 1.869 | 0.000868 |
| Q14671 | PUM1 | 1.865 | 0.045687 |
| Q96J02 | ITCH | 1.85 | 0.005118 |
| Q9BVP2 | GNL3 | 1.839 | 0.048238 |
| P15497 | APOA1 | 1.839 | 0.011175 |
| Q9NR12 | PDLIM7 | 1.795 | 0.005118 |
| Q13615 | MTMR3 | 1.782 | 0.049633 |
| Q9UI10 | EIF2B4 | 1.732 | 0.04655 |
| Q9HC38 | GLOD4 | 1.701 | 0.005118 |
| Q96II8 | LRCH3 | 1.694 | 0.038576 |
| Q8WUM0 | NUP133 | 1.69 | 0.035662 |
| P11802 | CDK4 | 1.684 | 0.01676 |
| Q96A49 | SYAP1 | 1.667 | 0.028461 |
| Q13620 | CUL4B | 1.659 | 0.0259 |
| P42345 | MTOR | 1.652 | 0.015253 |
| Q8TEX9 | IPO4 | 1.652 | 0.024406 |

| | | | |
|---|---|---|---|
| P49790 | NUP153 | 1.642 | 0.029494 |
| Q86US8 | SMG6 | 1.64 | 0.00705 |
| P60953 | CDC42 | 1.636 | 0.014024 |
| Q9H2U1 | DHX36 | 1.634 | 0.008944 |
| P41743 | PRKCI | 1.633 | 0.006695 |
| Q9H490 | PIGU | 1.629 | 0.00439 |
| Q9Y3I1 | FBXO7 | 1.627 | 0.024824 |
| Q9NP79 | VTA1 | 1.624 | 0.041746 |
| P28290 | ITPRID2 | 1.616 | 0.01573 |
| Q7Z460 | CLASP1 | 1.607 | 0.045668 |
| Q9NT62 | ATG3 | 1.607 | 0.002797 |
| Q96Q11 | TRNT1 | 1.602 | 0.048481 |
| Q99439 | CNN2 | 1.601 | 0.006891 |
| O60573 | EIF4E2 | 1.599 | 0.019913 |
| P40937 | RFC5 | 1.598 | 0.041803 |
| P20936 | RASA1 | 1.592 | 0.007755 |
| Q92973 | TNPO1 | 1.582 | 0.001514 |
| Q9NR09 | BIRC6 | 1.577 | 0.004492 |
| O94979 | SEC31A | 1.577 | 0.046518 |
| Q9Y2T2 | AP3M1 | 1.575 | 0.004706 |
| Q9NSD9 | FARSB | 1.57 | 0.012439 |
| Q9UHI6 | DDX20 | 1.568 | 0.000713 |
| Q9ULA0 | DNPEP | 1.565 | 0.038517 |
| O75886 | STAM2 | 1.554 | 0.00787 |
| Q9NXF1 | TEX10 | 1.552 | 0.007074 |
| P07737 | PFN1 | 1.539 | 0.026815 |
| P56192 | MARS1 | 1.534 | 0.008096 |
| Q8N3C0 | ASCC3 | 1.529 | 0.008678 |
| P08559 | PDHA1 | 1.526 | 0.01803 |
| P23258 | TUBG1 | 1.525 | 0.028535 |
| Q9HC35 | EML4 | 1.523 | 0.010363 |
| P50213 | IDH3A | 1.52 | 0.02421 |
| Q9Y5B9 | SUPT16H | 1.518 | 0.01406 |
| P00403 | MT-CO2 | 1.511 | 0.037438 |
| Q8IW35 | CEP97 | 1.509 | 0.033295 |
| Q15717 | ELAVL1 | 1.505 | 0.019454 |
| Q9NRG9 | AAAS | 1.504 | 0.033208 |
| O14737 | PDCD5 | 1.504 | 0.02501 |

Supplemental Table 4:
B1B2C/B1B2 Abundance vs B1B2C$^{P353A}$/B1B2C Ratio

| Protein | B1B2C/B1B2 | B1B2C$^{P353A}$/B1B2C |
|---|---|---|
| SKP2 | 2.706 | 1.106 |
| MAEA | 2.873 | 1.297 |
| PSMD1 | 2.588 | 1.233 |
| PSMD4 | 2.308 | 1.104 |
| PSMD13 | 2.133 | 1.304 |
| PPP1CB | 2.508 | 1.416 |
| WIPI2 | 2.004 | 0.879 |
| ARHGAP29 | 12.37 | 0.818 |
| AIFM | 6.109 | 0.16 |
