# Supplementary material for: Evidence that S‐phase kinase associated protein 2 (SKP2) is ubiquitinated and degraded via a Rho‐related BTB domain containing 1 (RhoBTB1) and Cullin‐3 mechanism in placenta
Source: Physiol Rep. 2026 Aug 4;14(15):e71039. doi: 10.14814/phy2.71039 (PMC13437954; doi:10.14814/phy2.71039)

Figure 6 Original

SKP1
Reversed

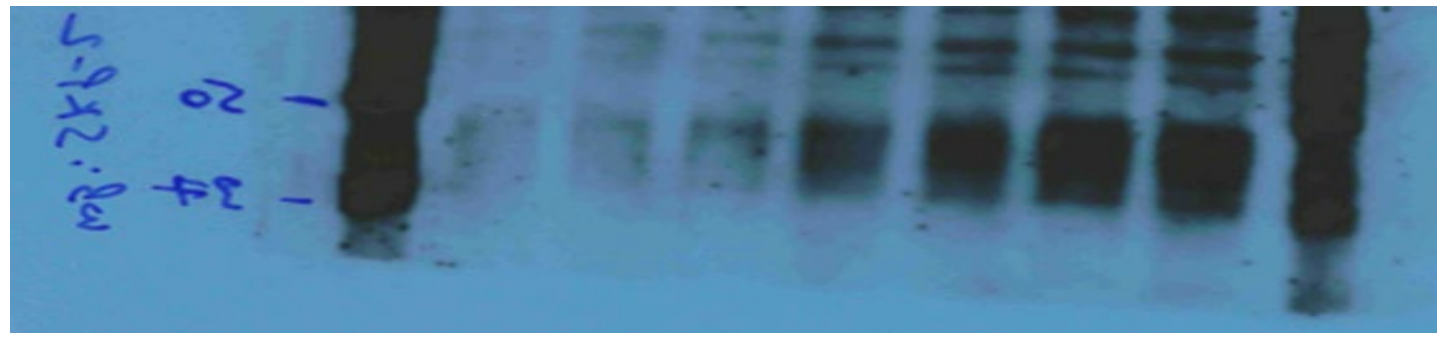

ArhGAP29
Reversed

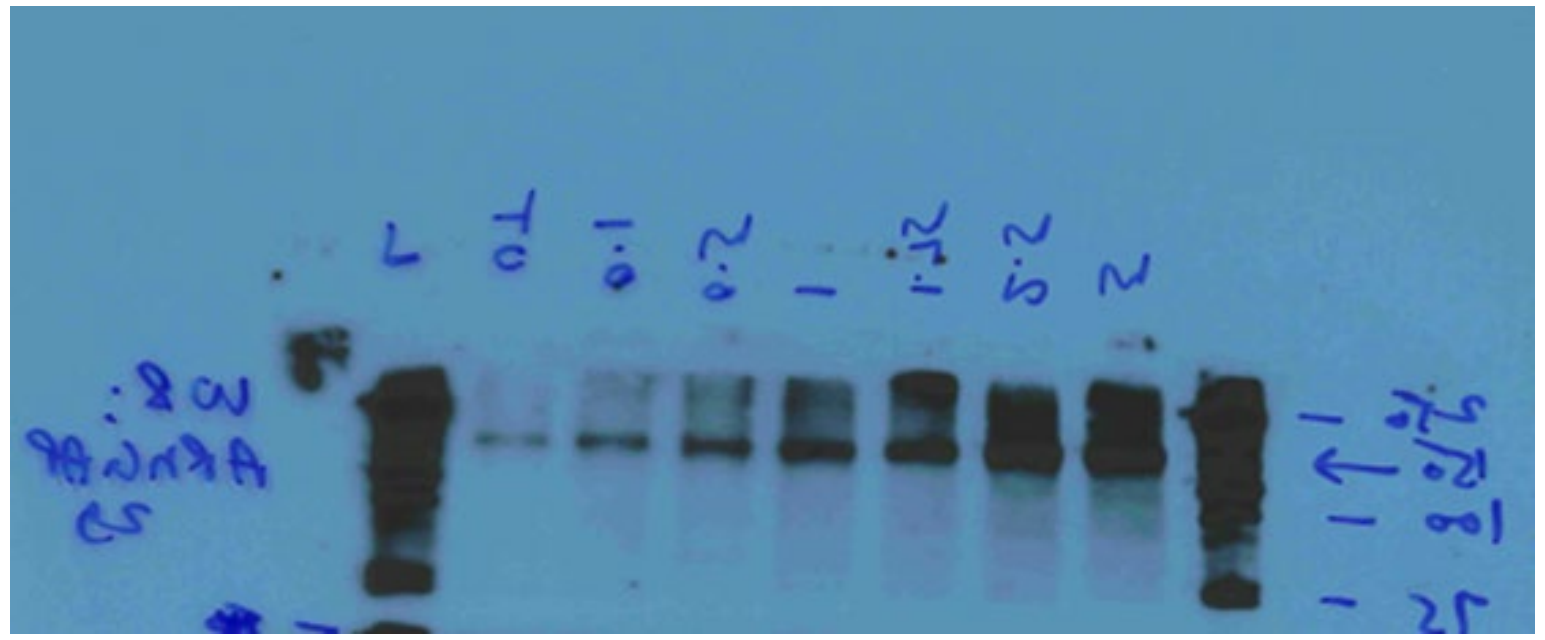

WIPI2

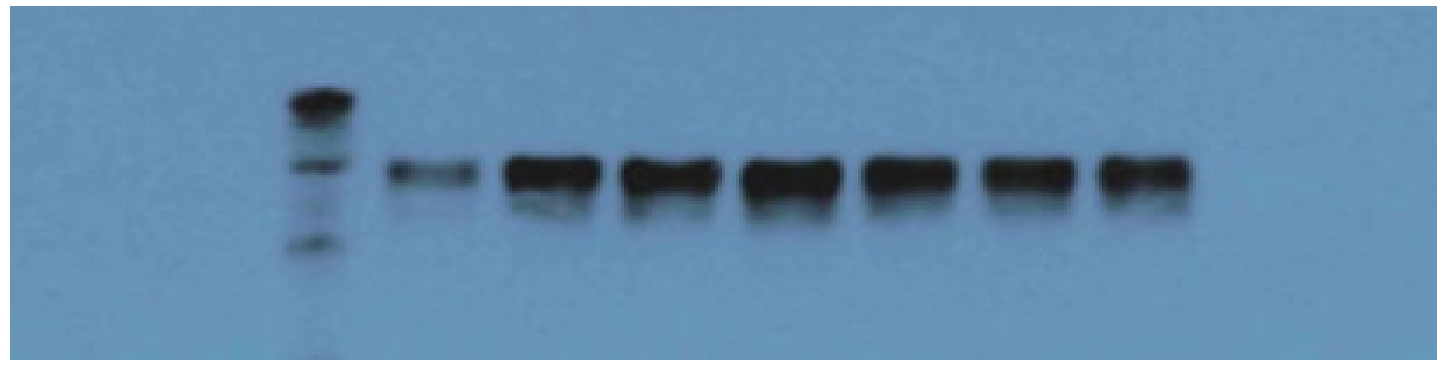

PPP1CB

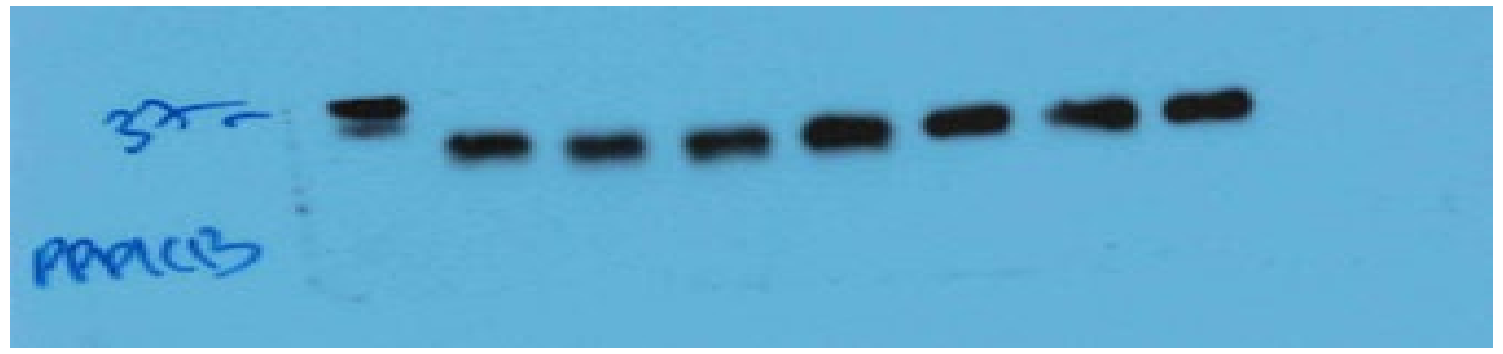

MAEA

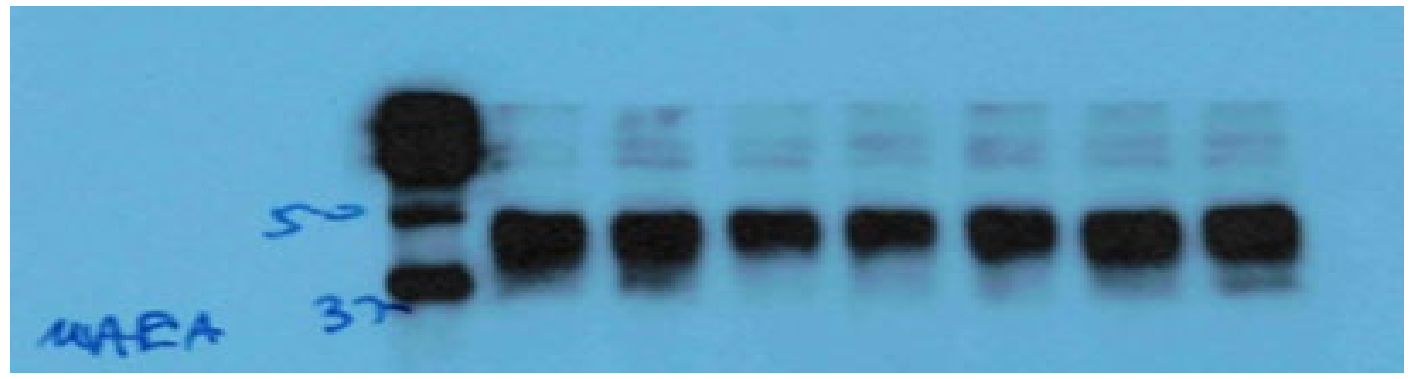

PSMD1

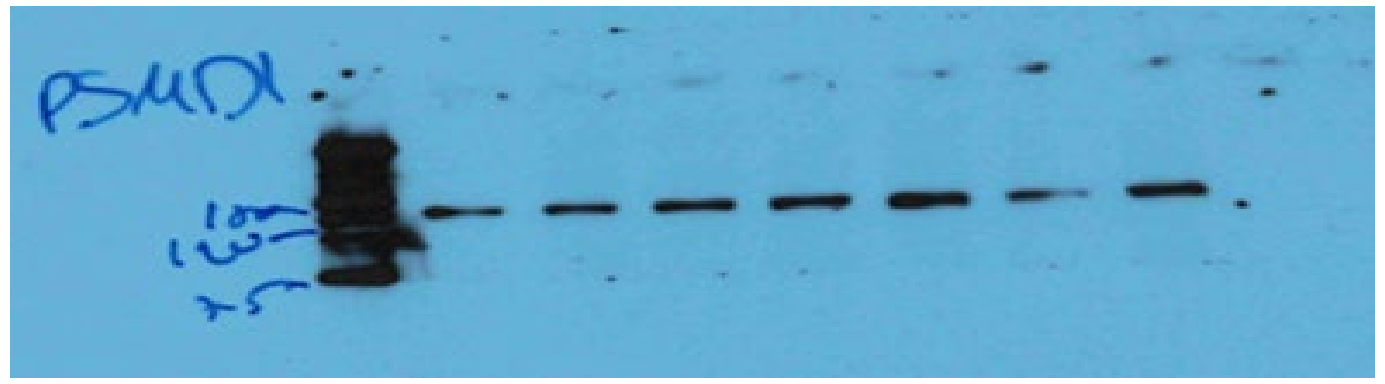

PSMD4

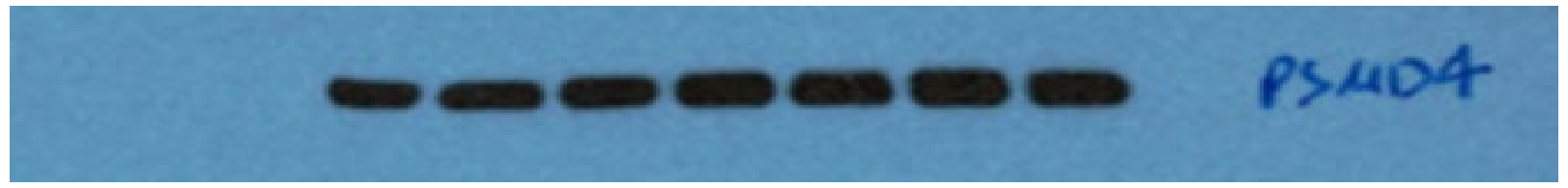

PSMD13

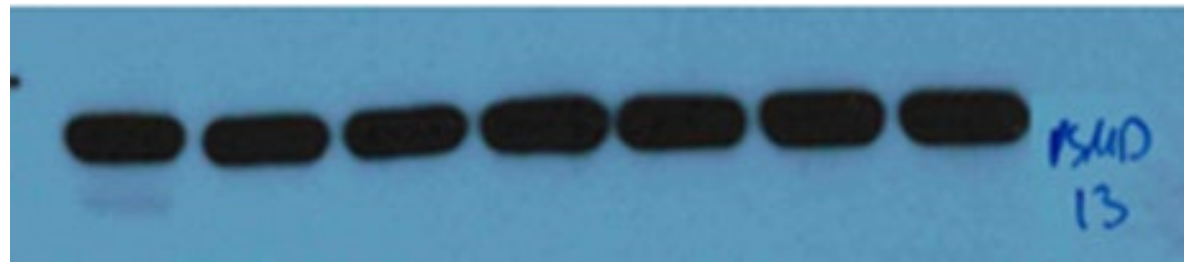

AIFM

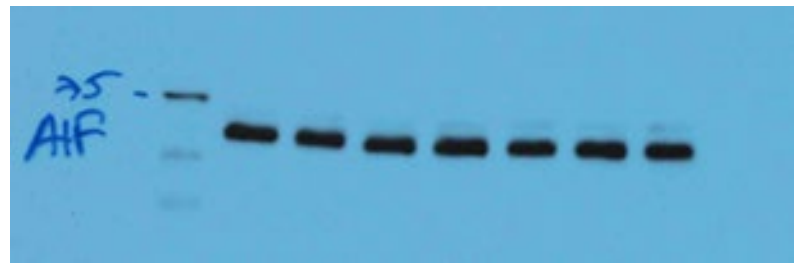

HSP90

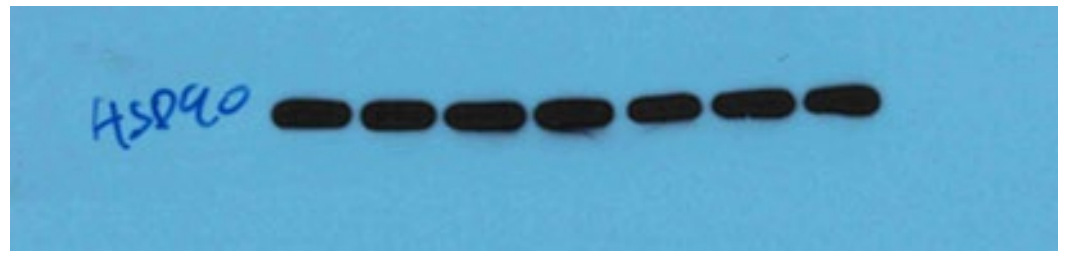

Figure 6: Color Shifted to Gray Scale

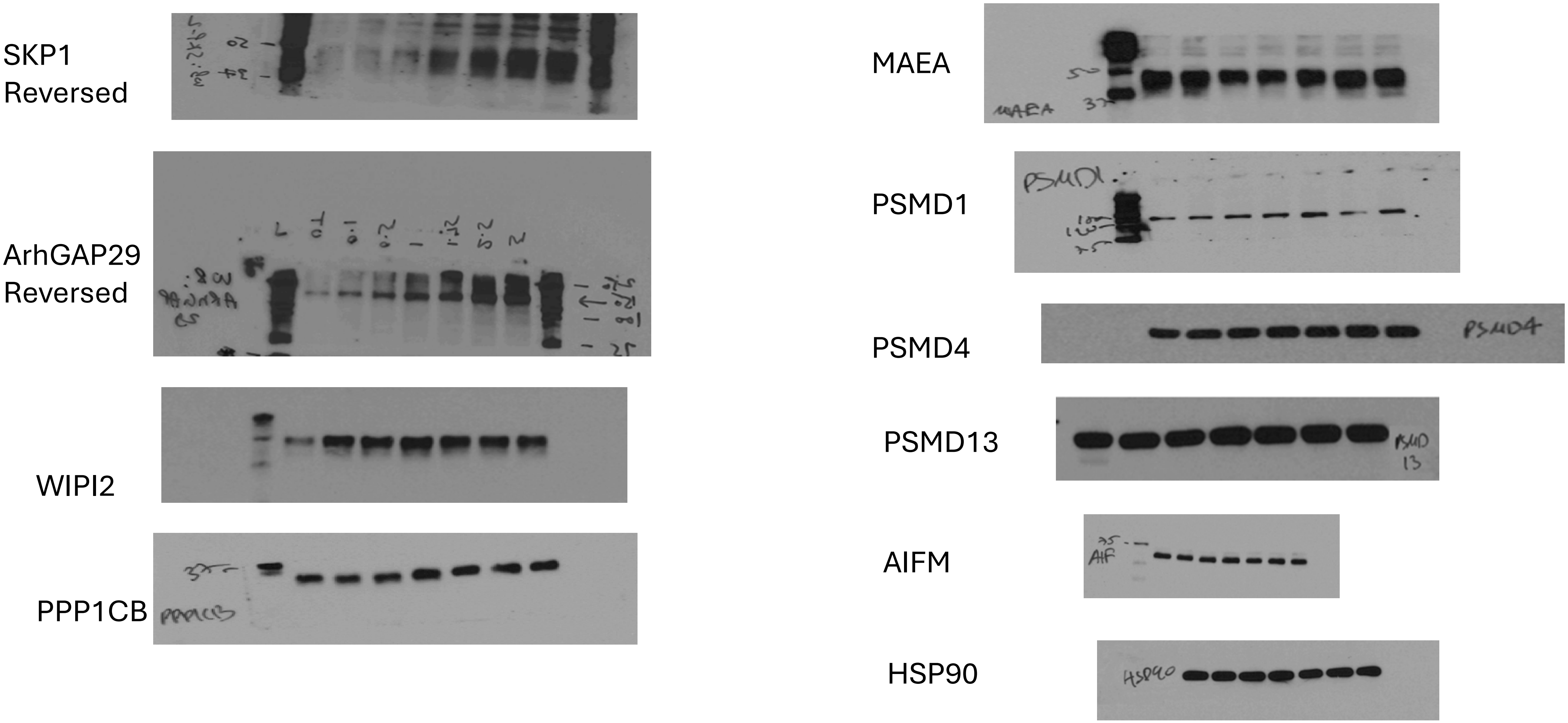

Figure 6: Cropped to Width, Aligned

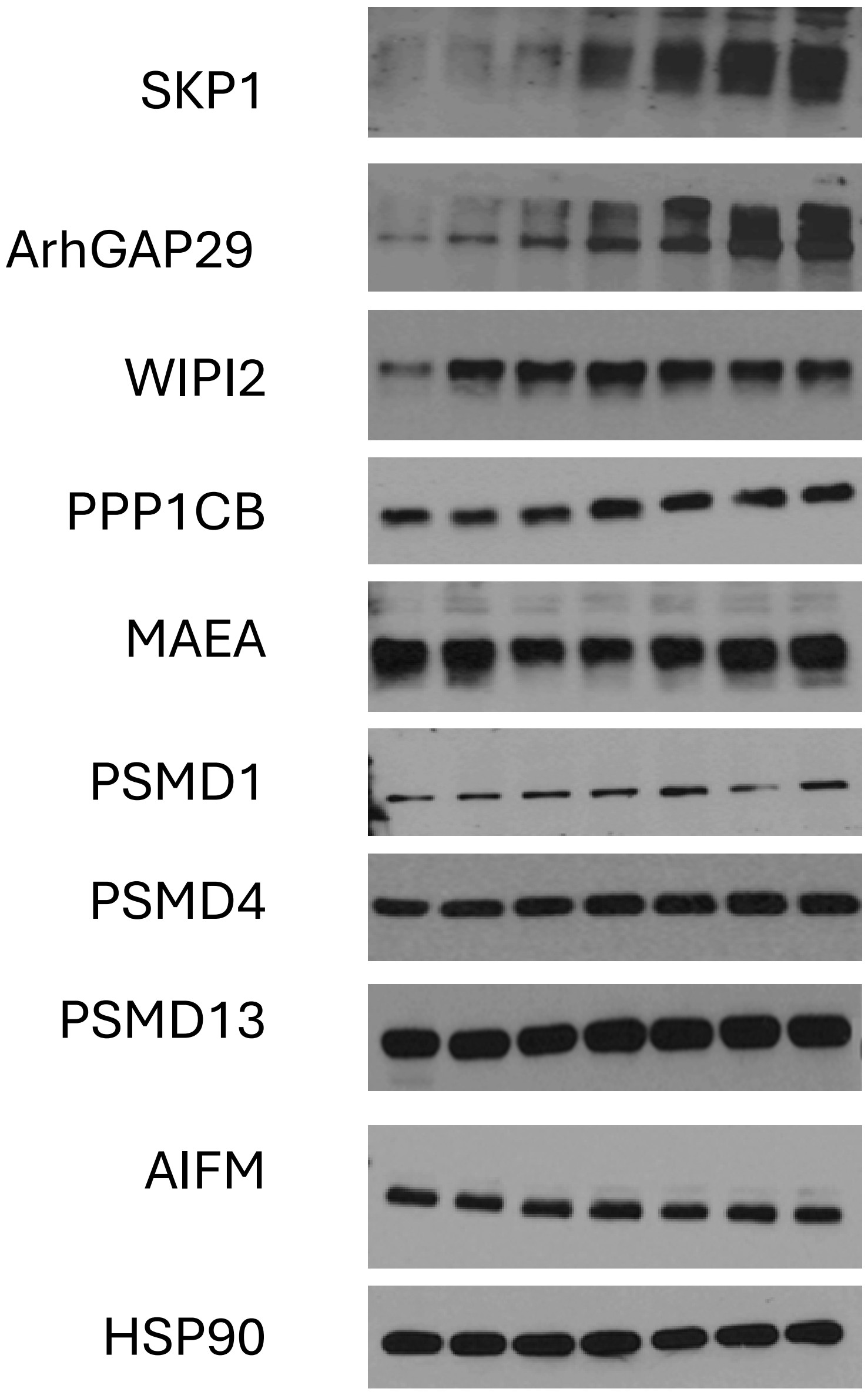

Figure 6

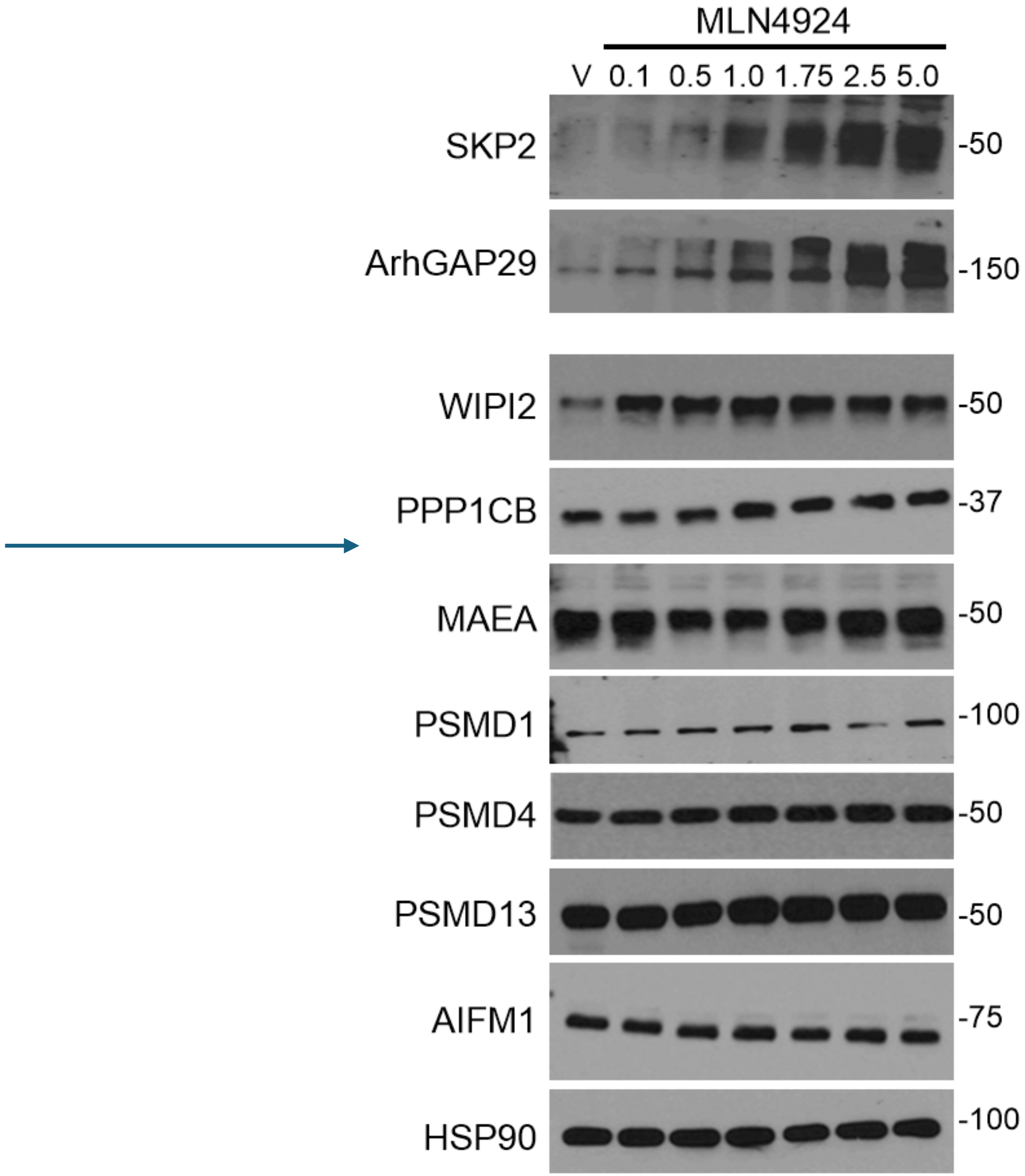

Figure 7 Original

Blot 1 and 4

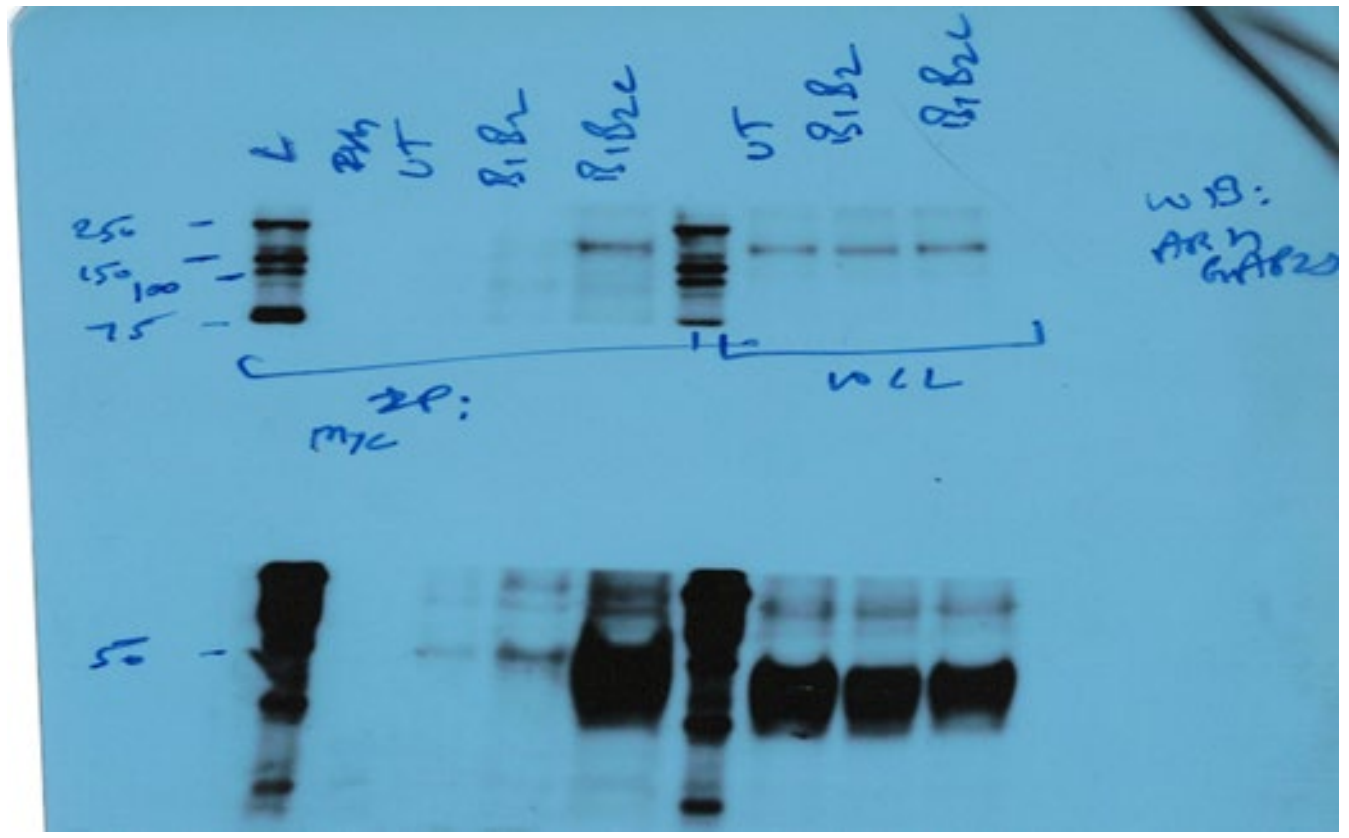

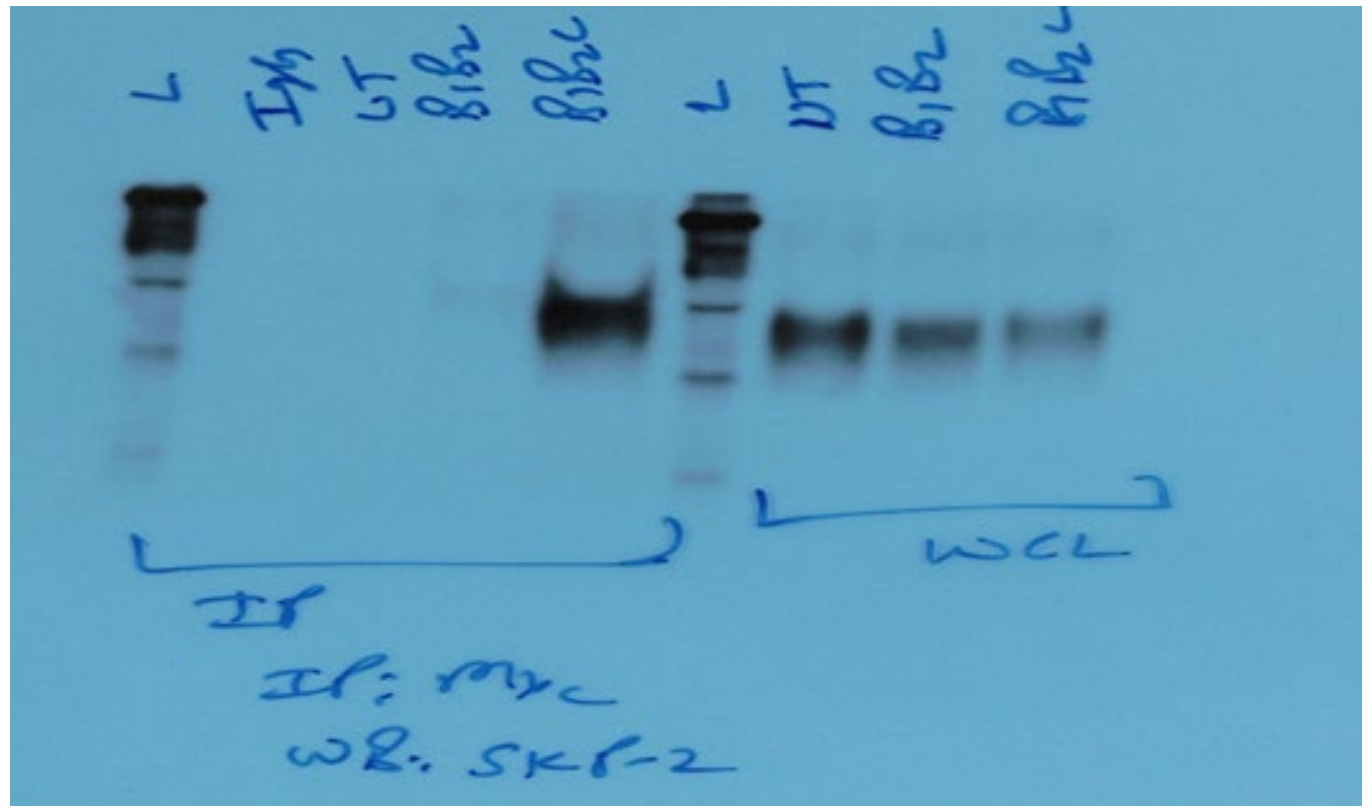

Blot 2 and 5

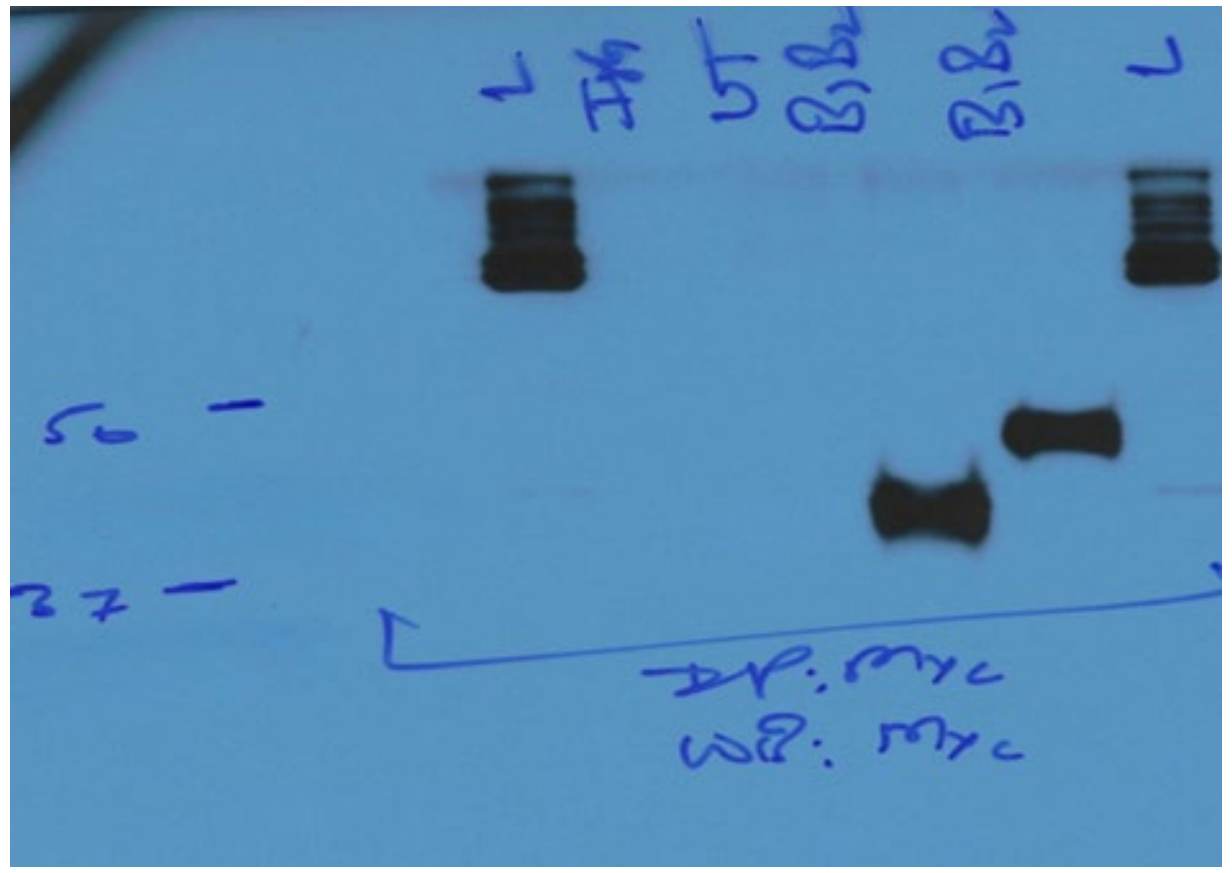

Blot 3

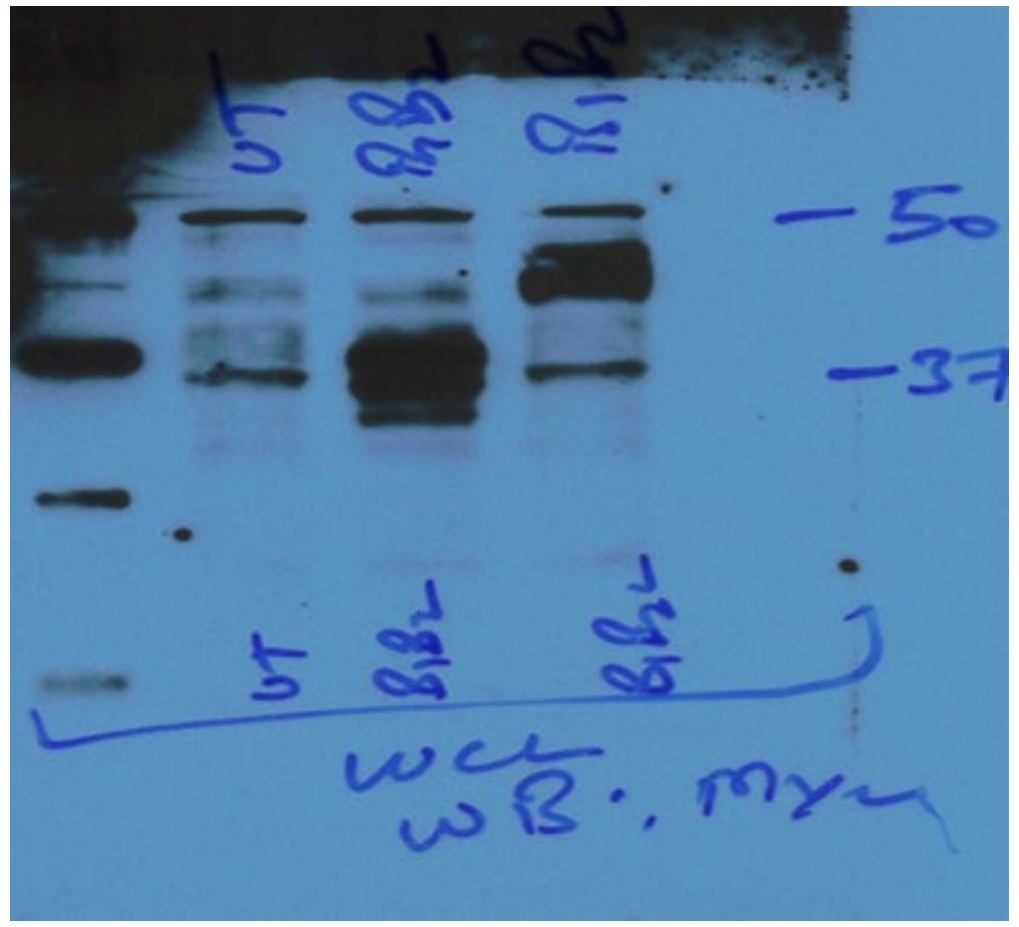

Blot 6

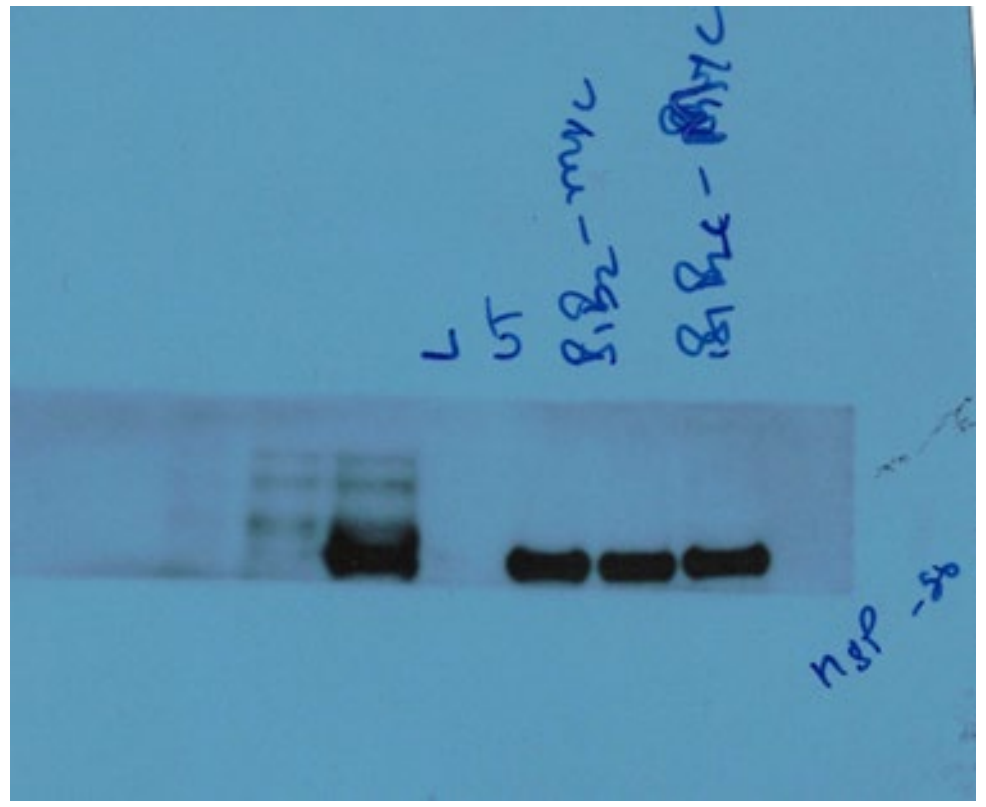

Blot 7

Figure 7 Converted to Gray Scale

Blot 1 and 4

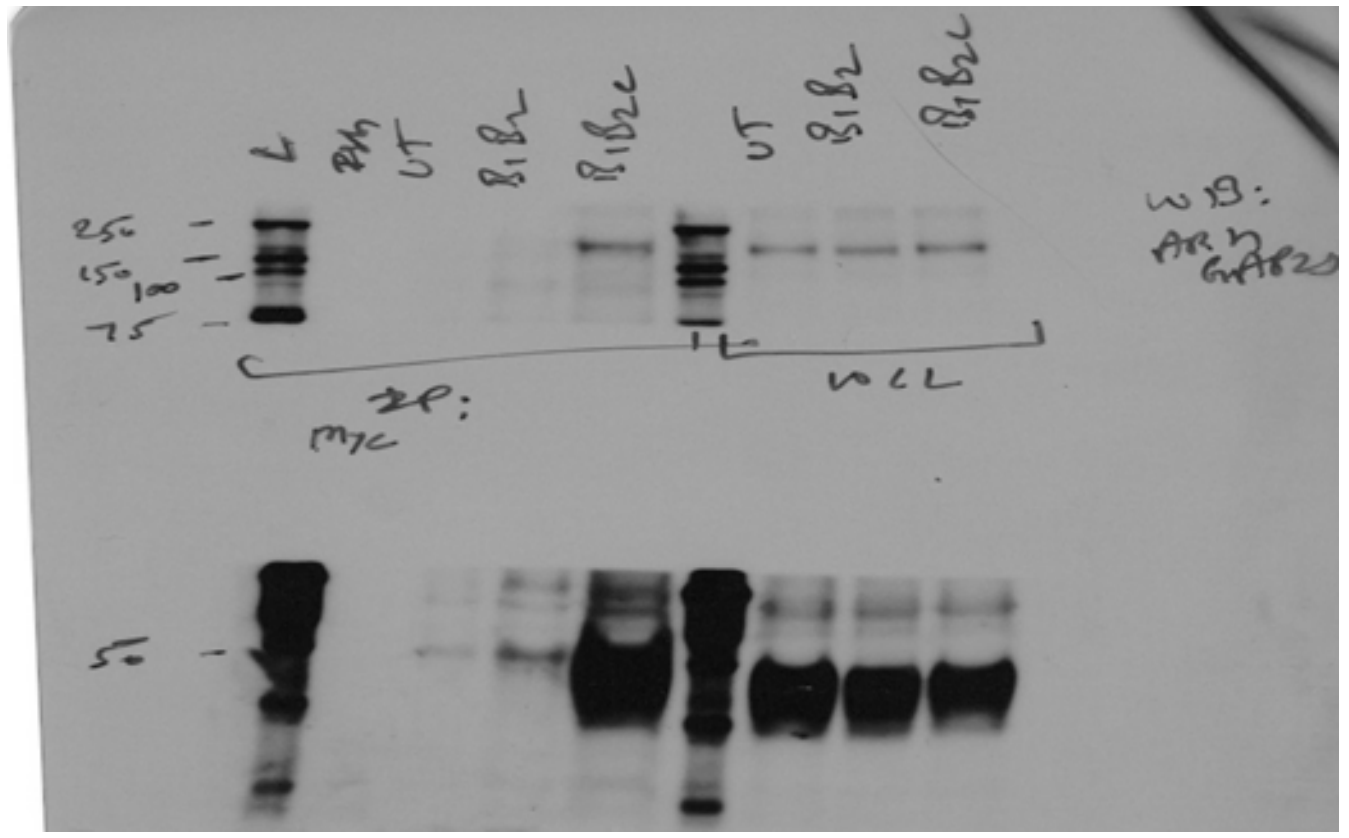

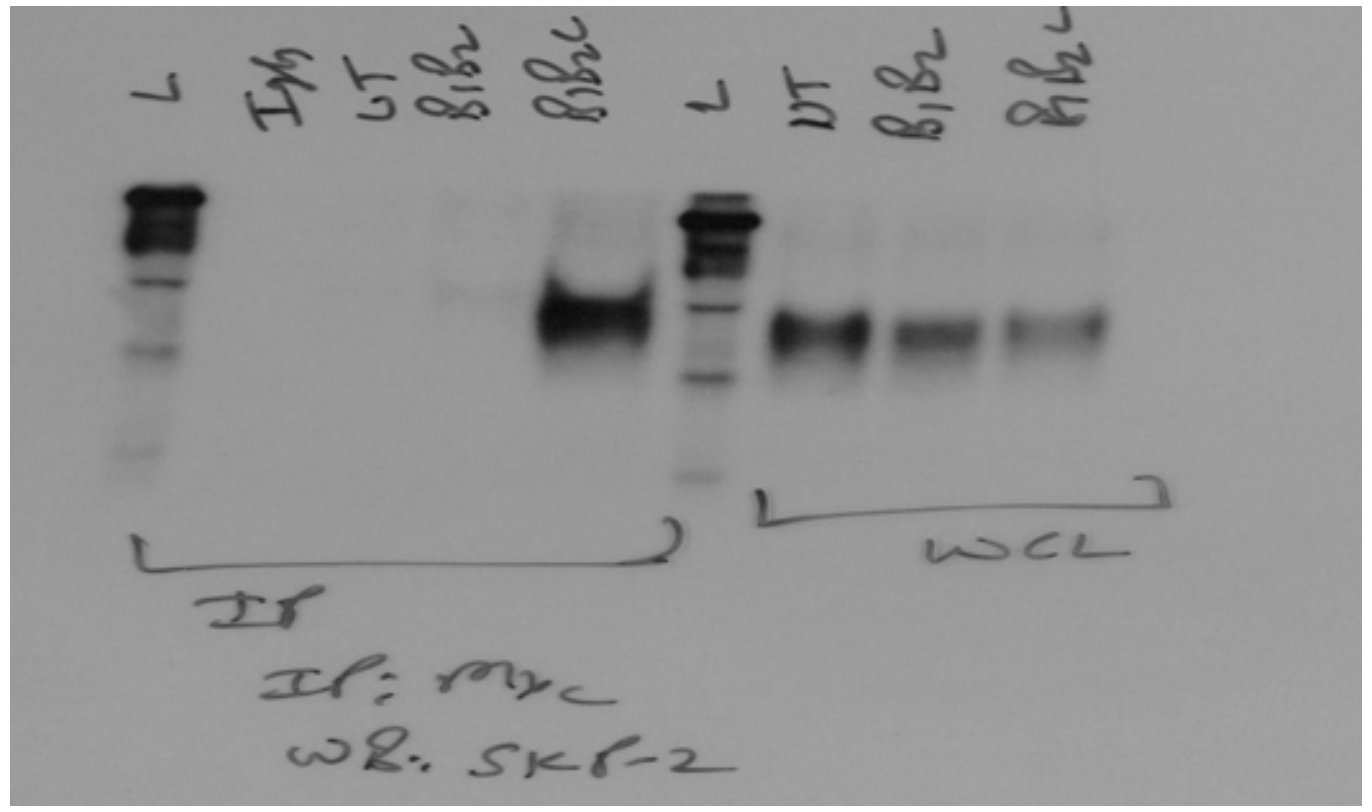

Blot 2 and 5

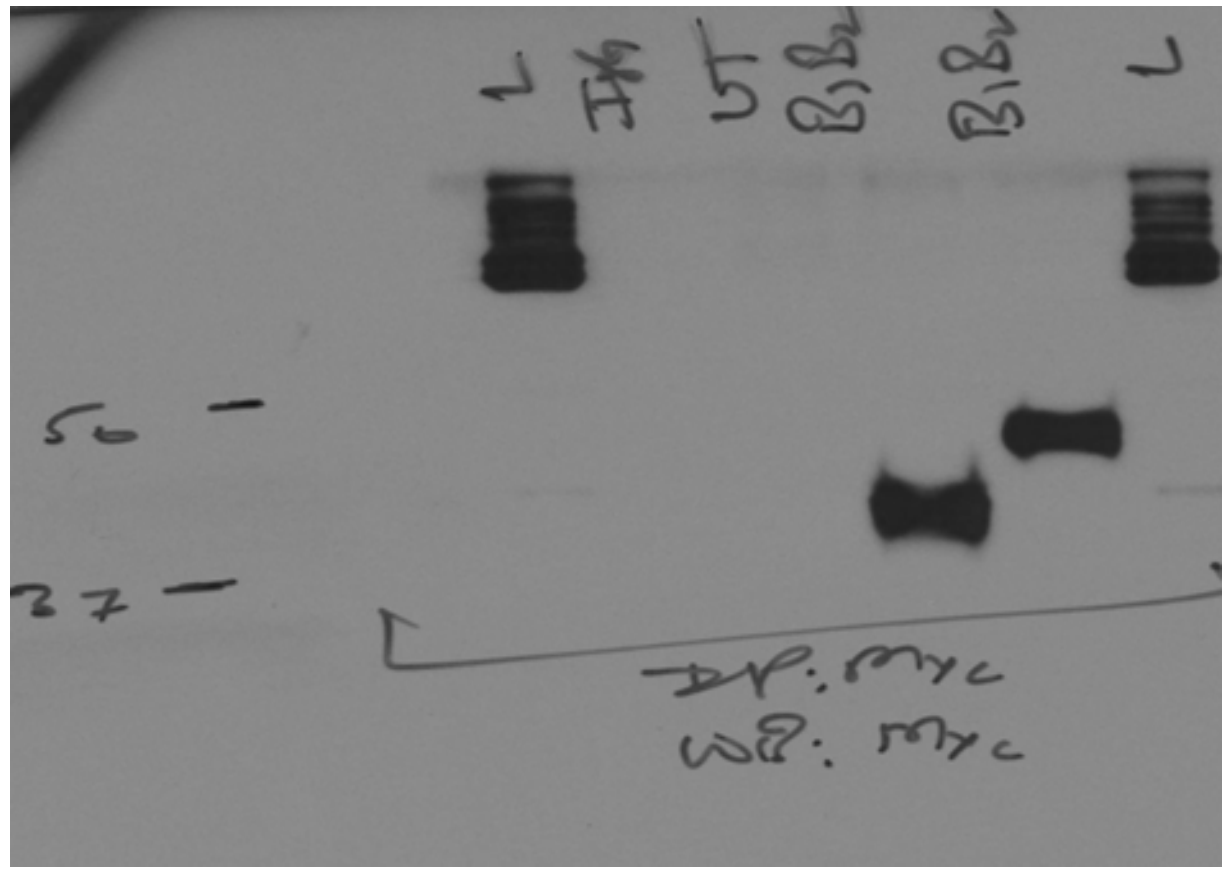

Blot 3

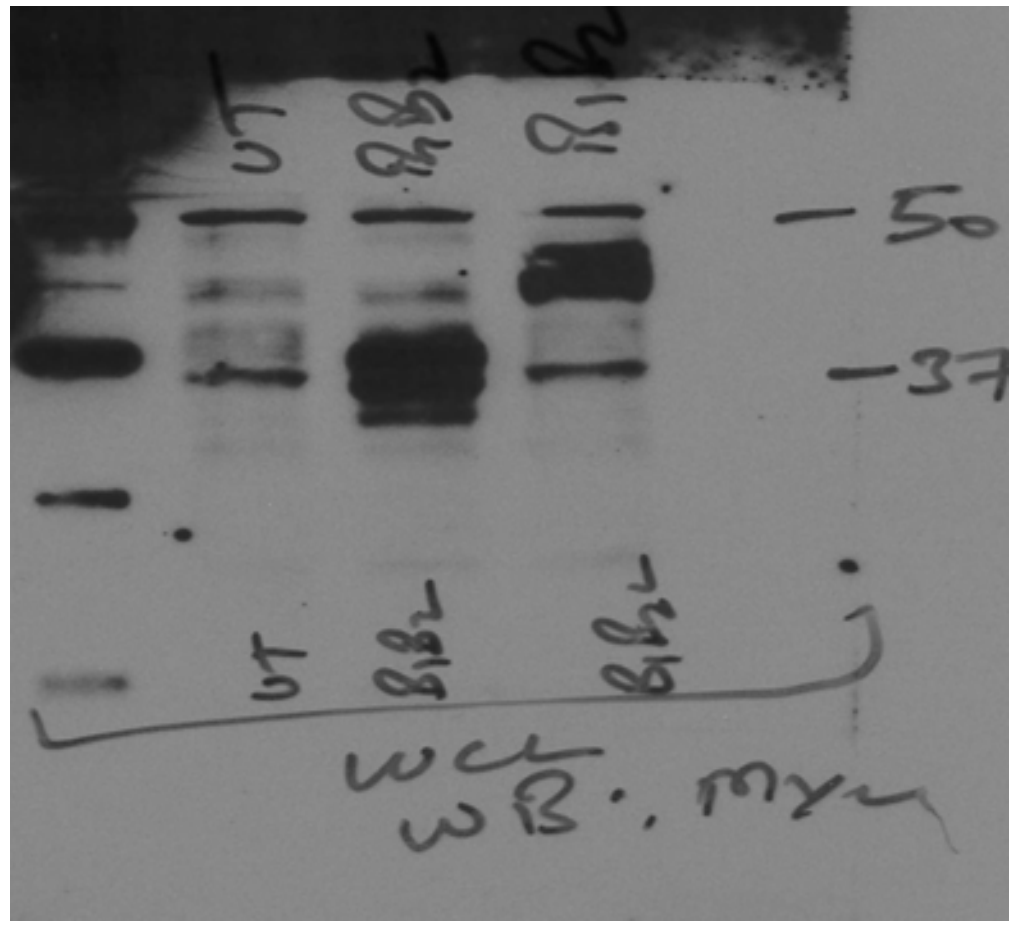

Blot 6

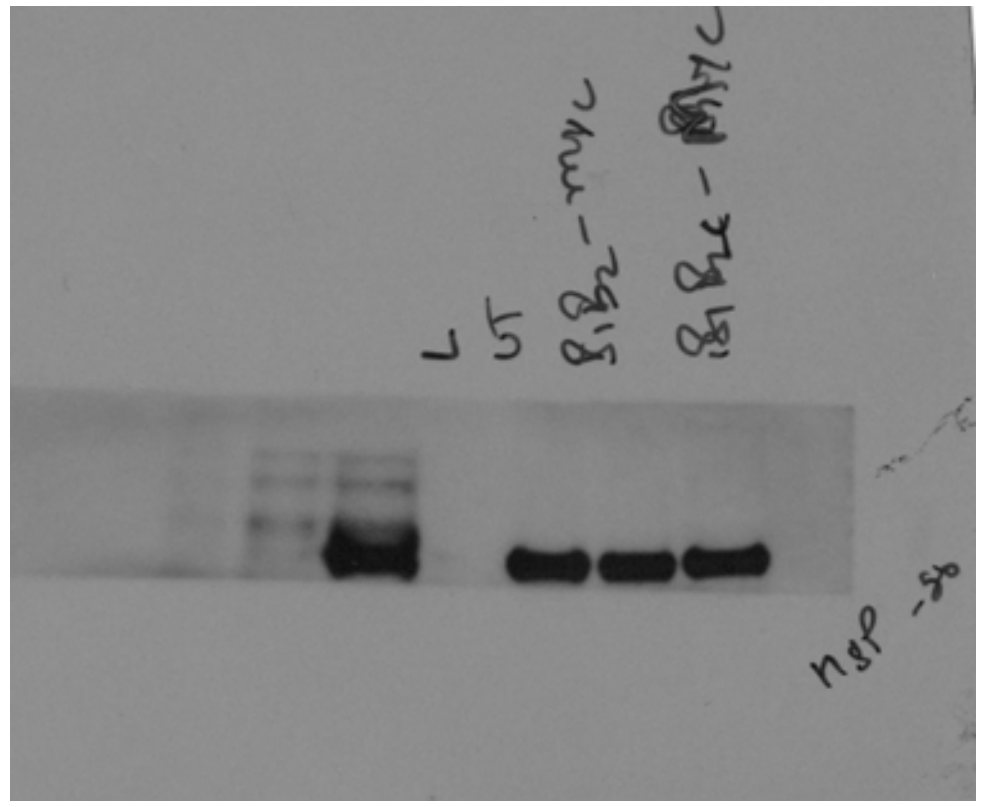

Blot 7

Figure 7 Cropped and Aligned

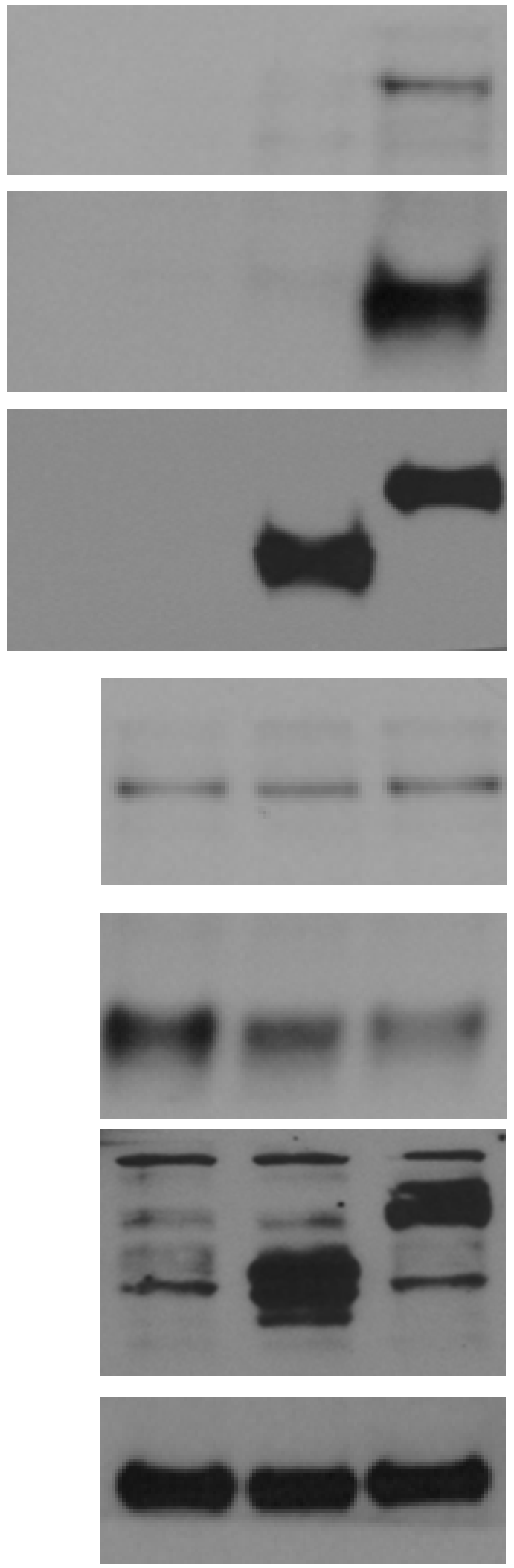

Figure 7

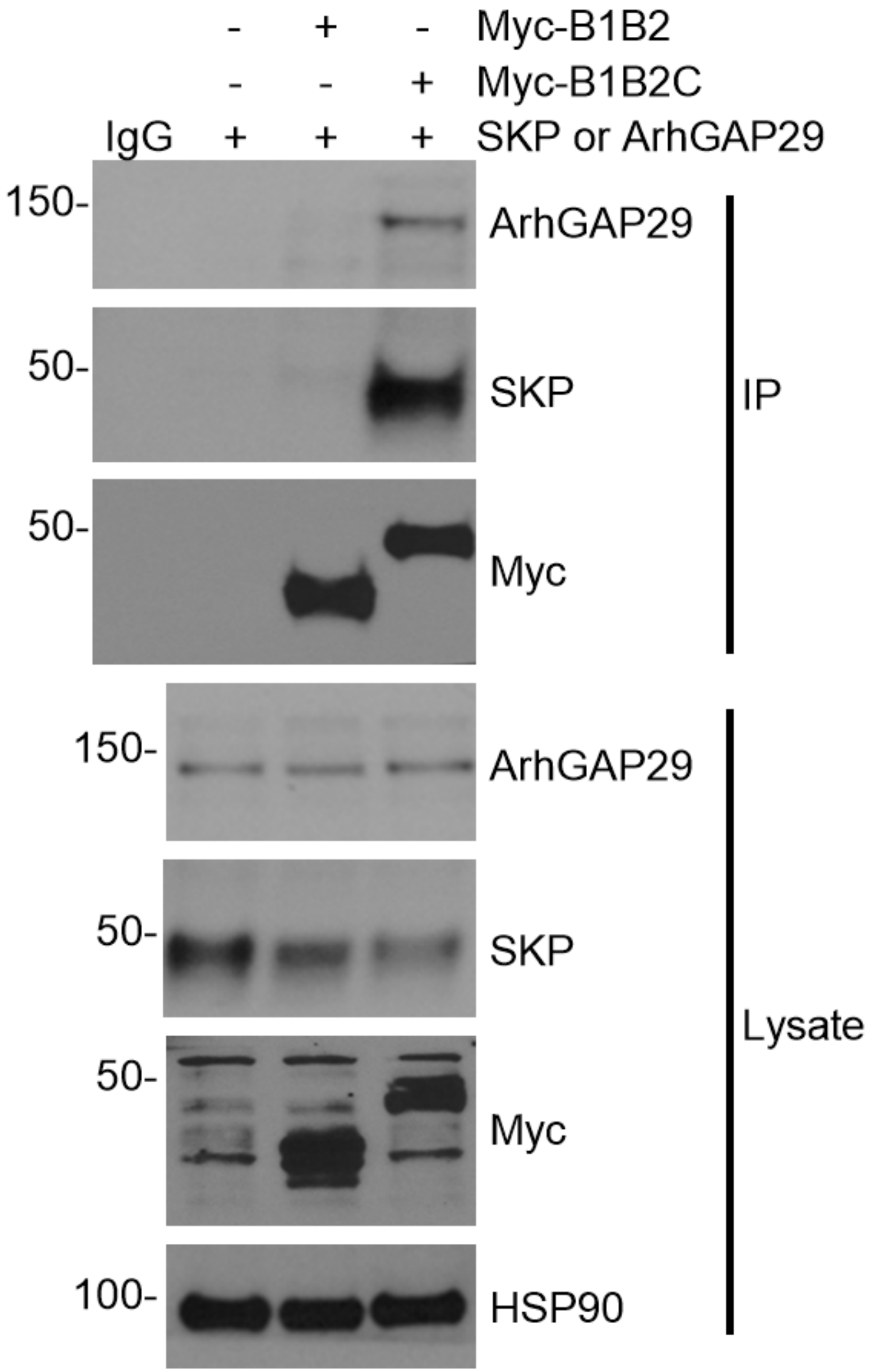

Figure 8A Original

CUL3

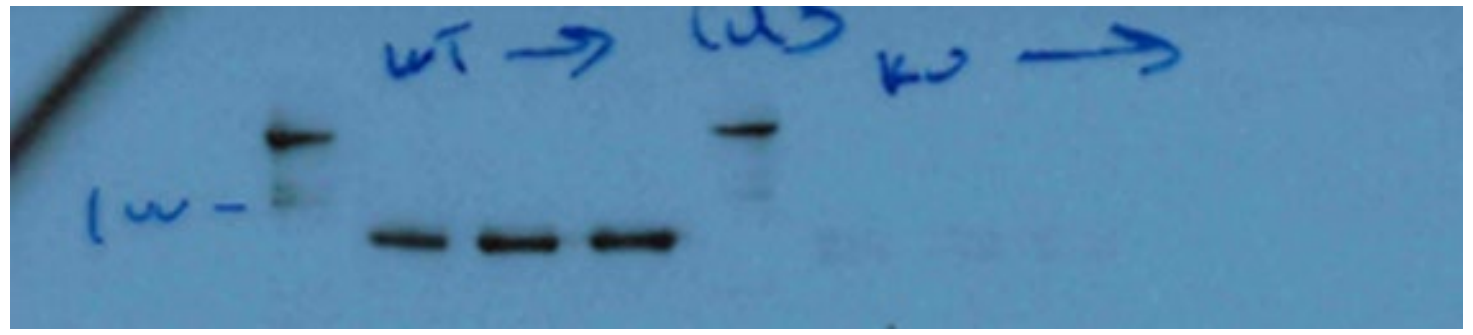

SKP2

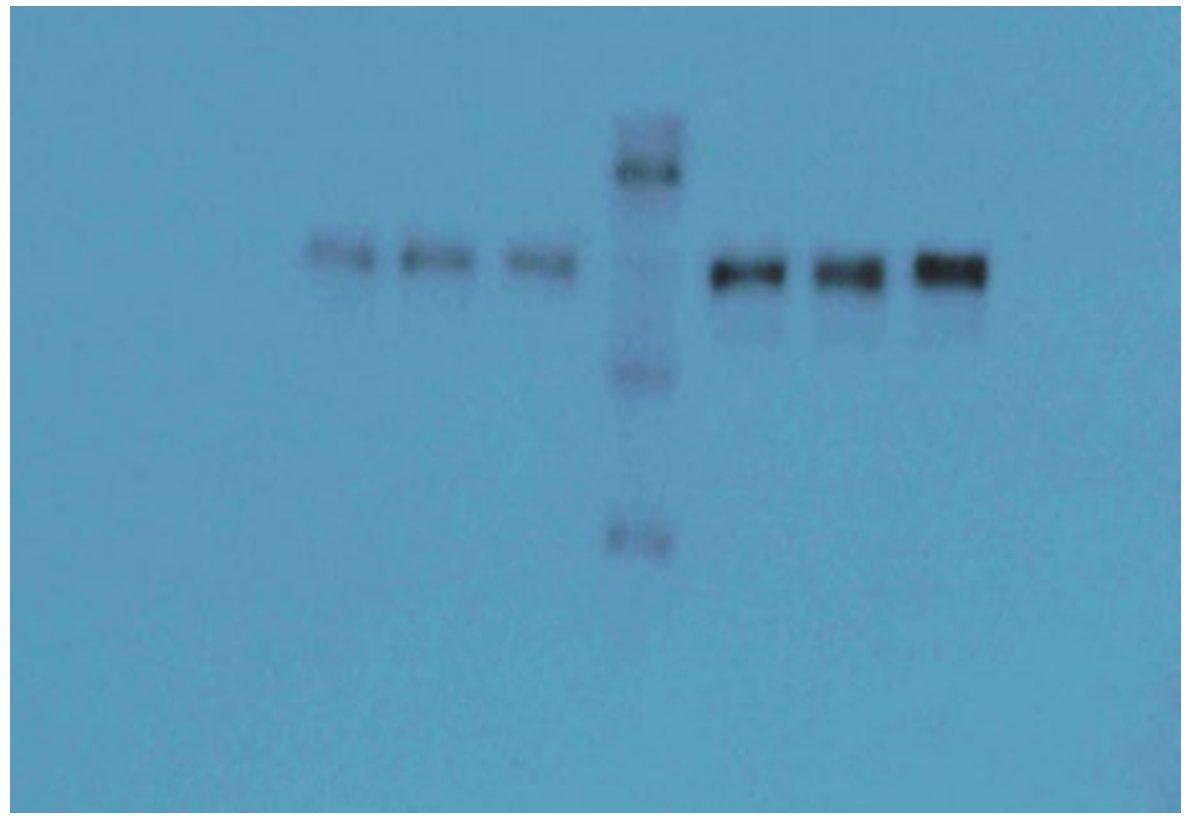

HSP90

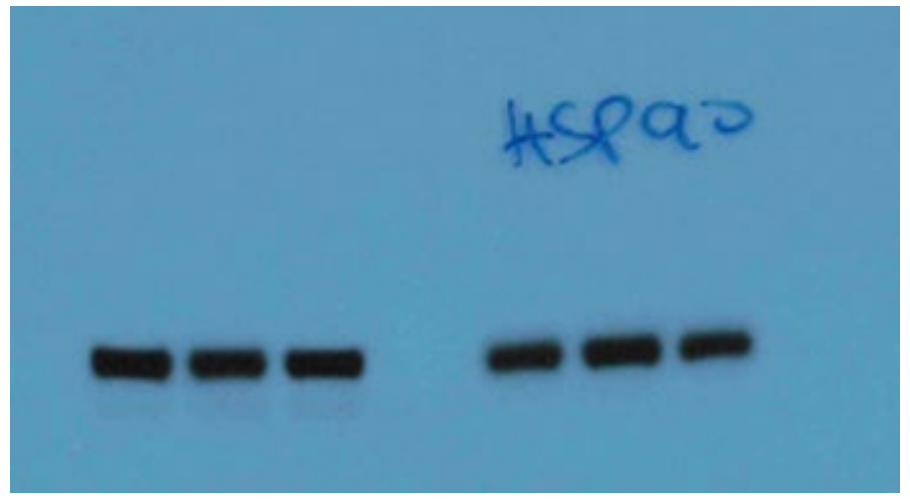

Figure 8A Gray Scale

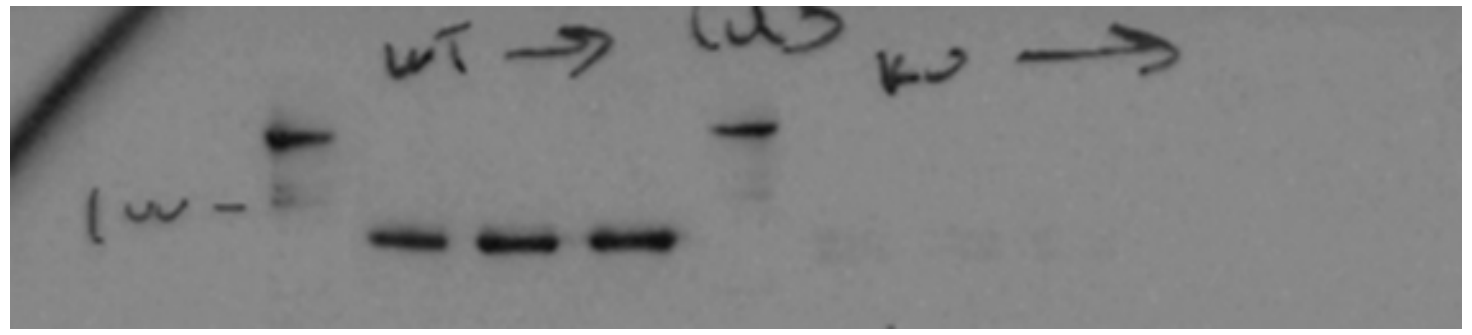

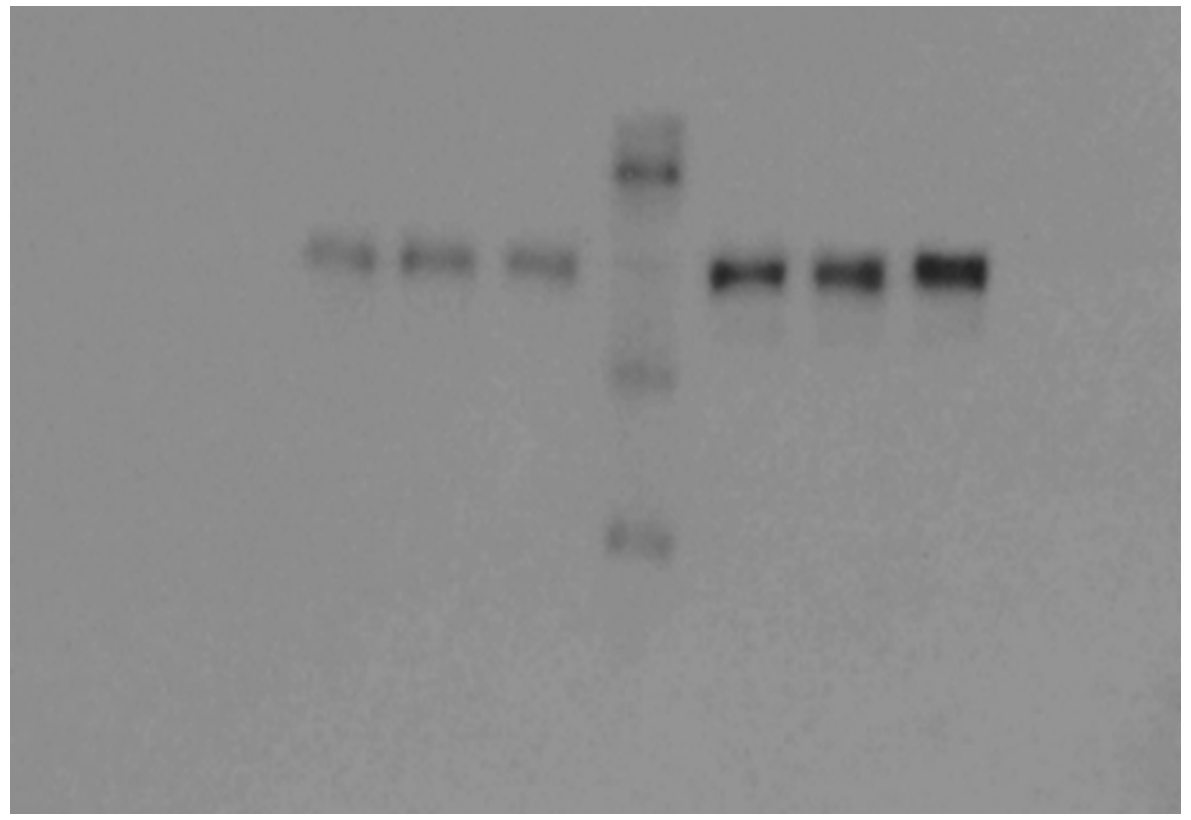

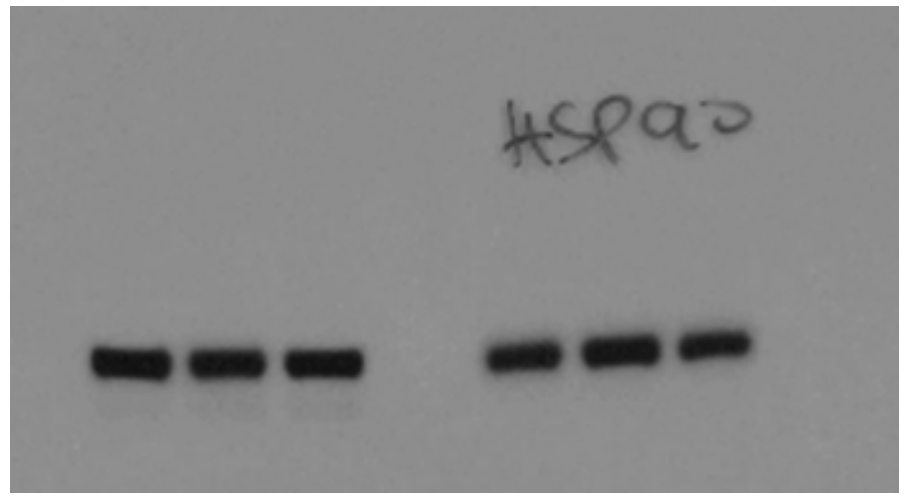

Figure 8A Cropped and Aligned

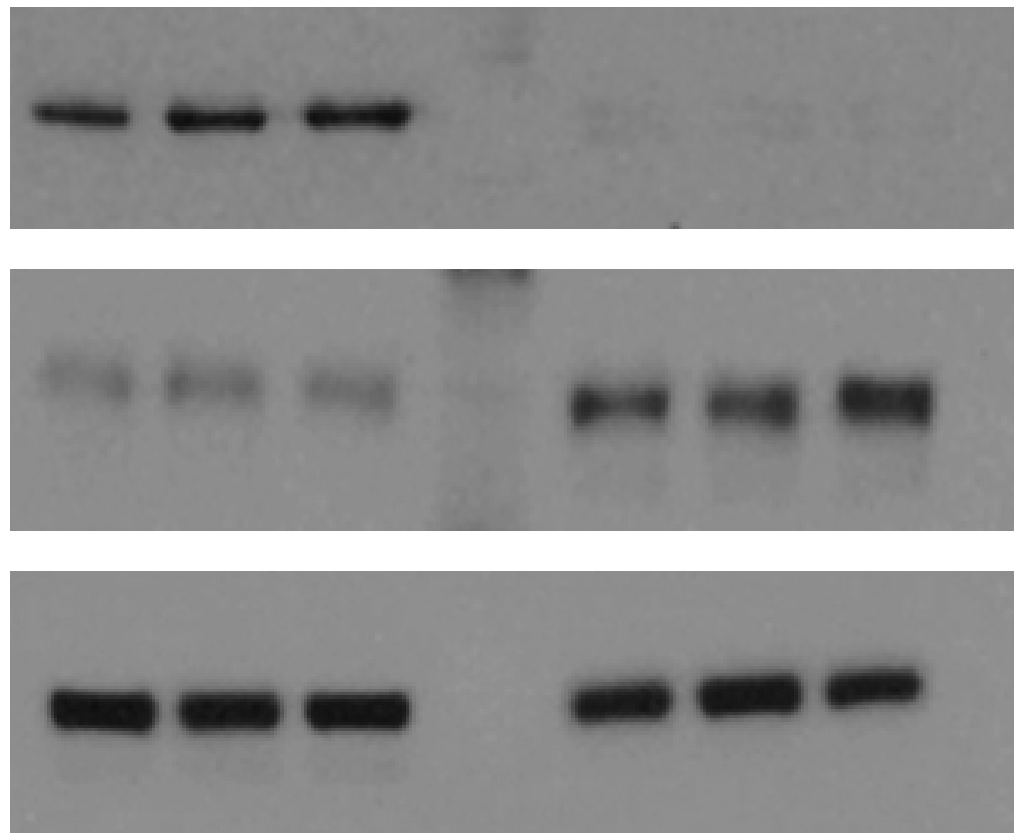

Figure 8A

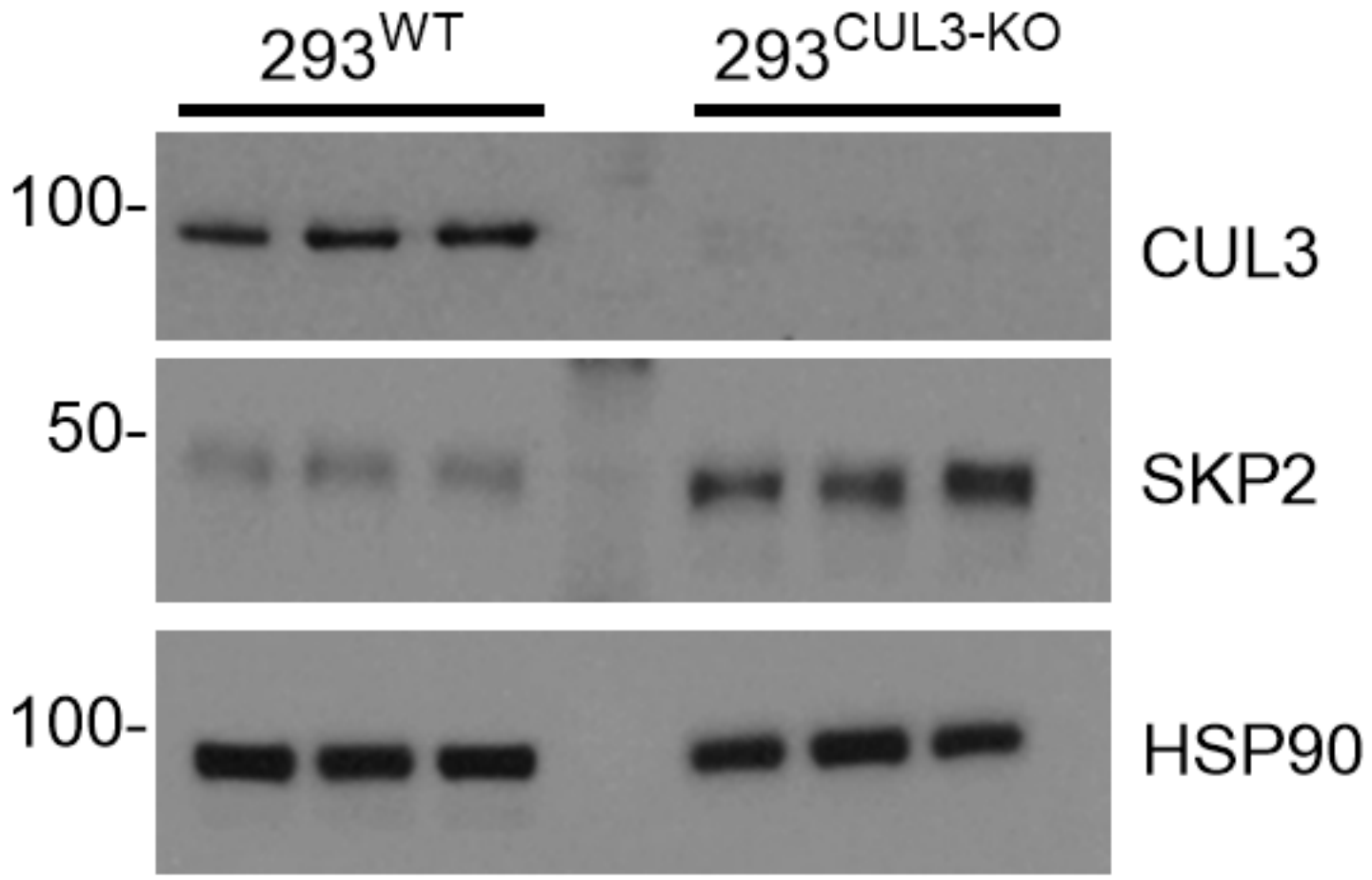

Figure 8B Original

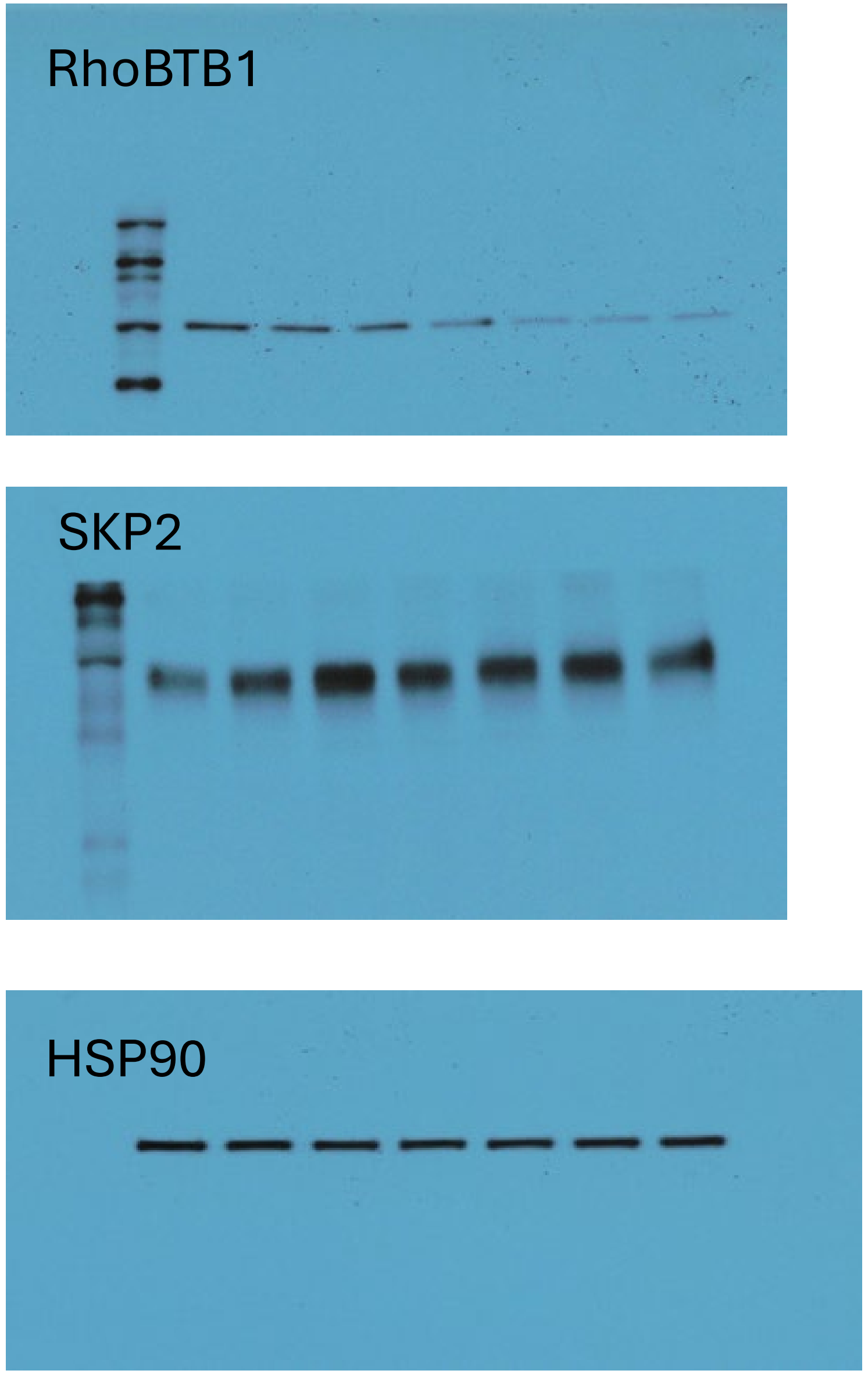

Figure 8B Gray Scale

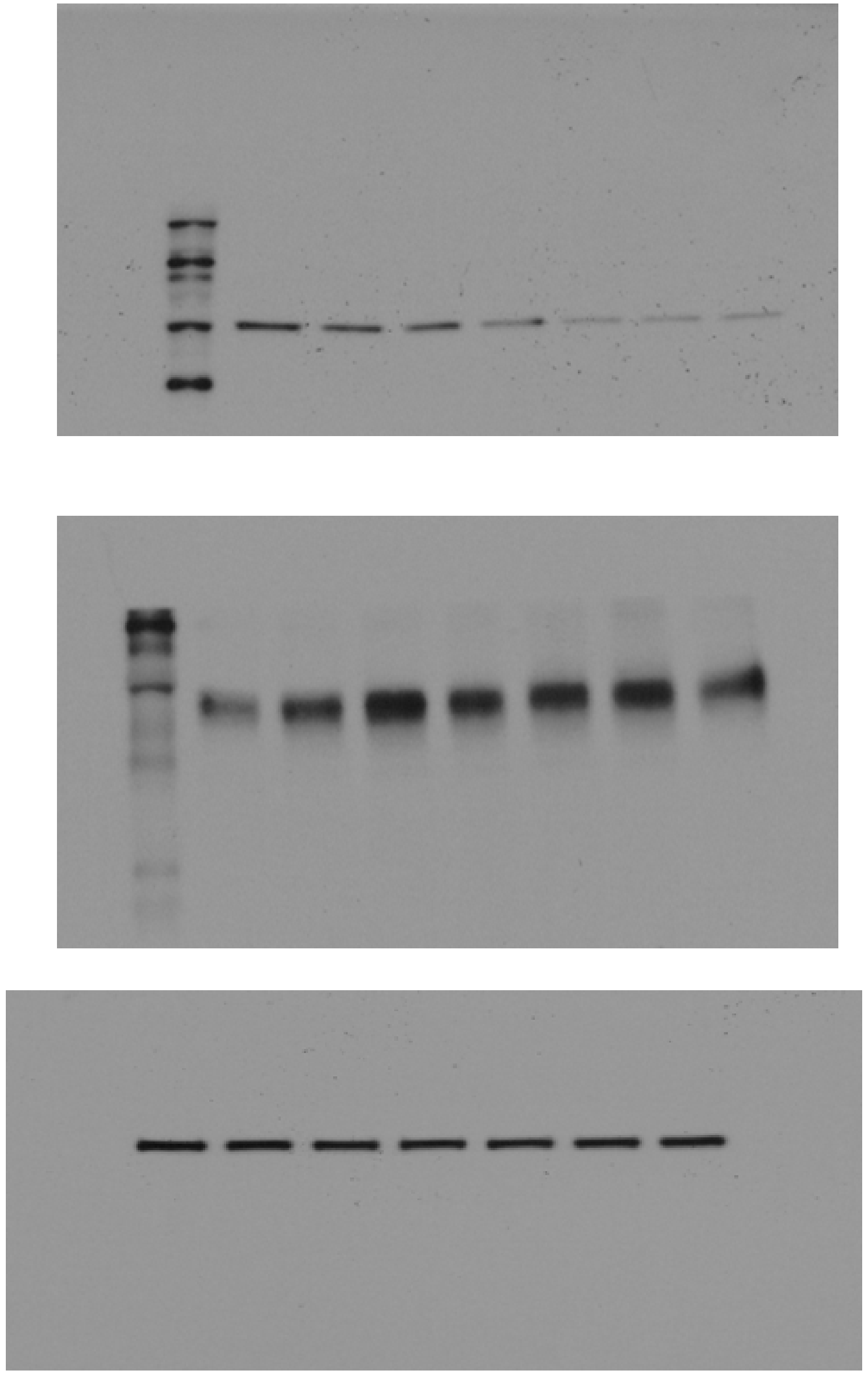

Figure 8B Cropped and Aligned

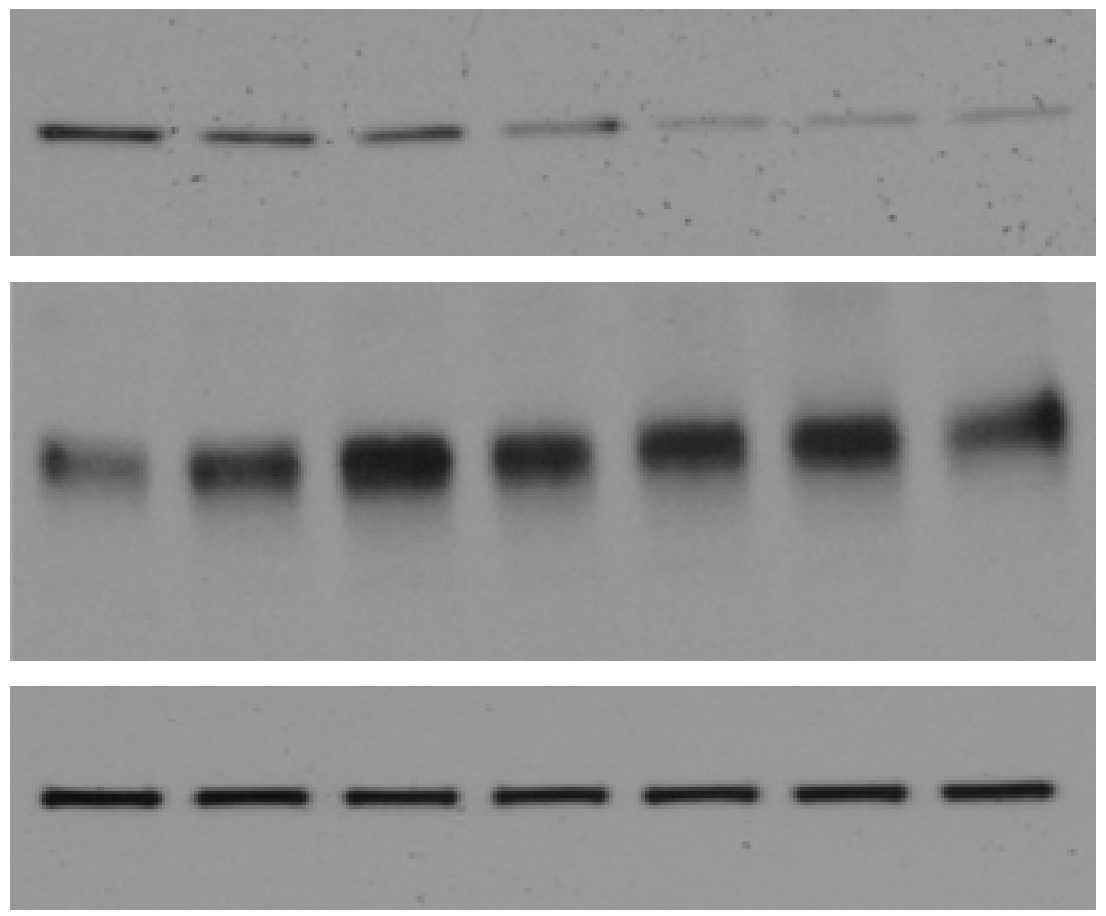

Figure 8B

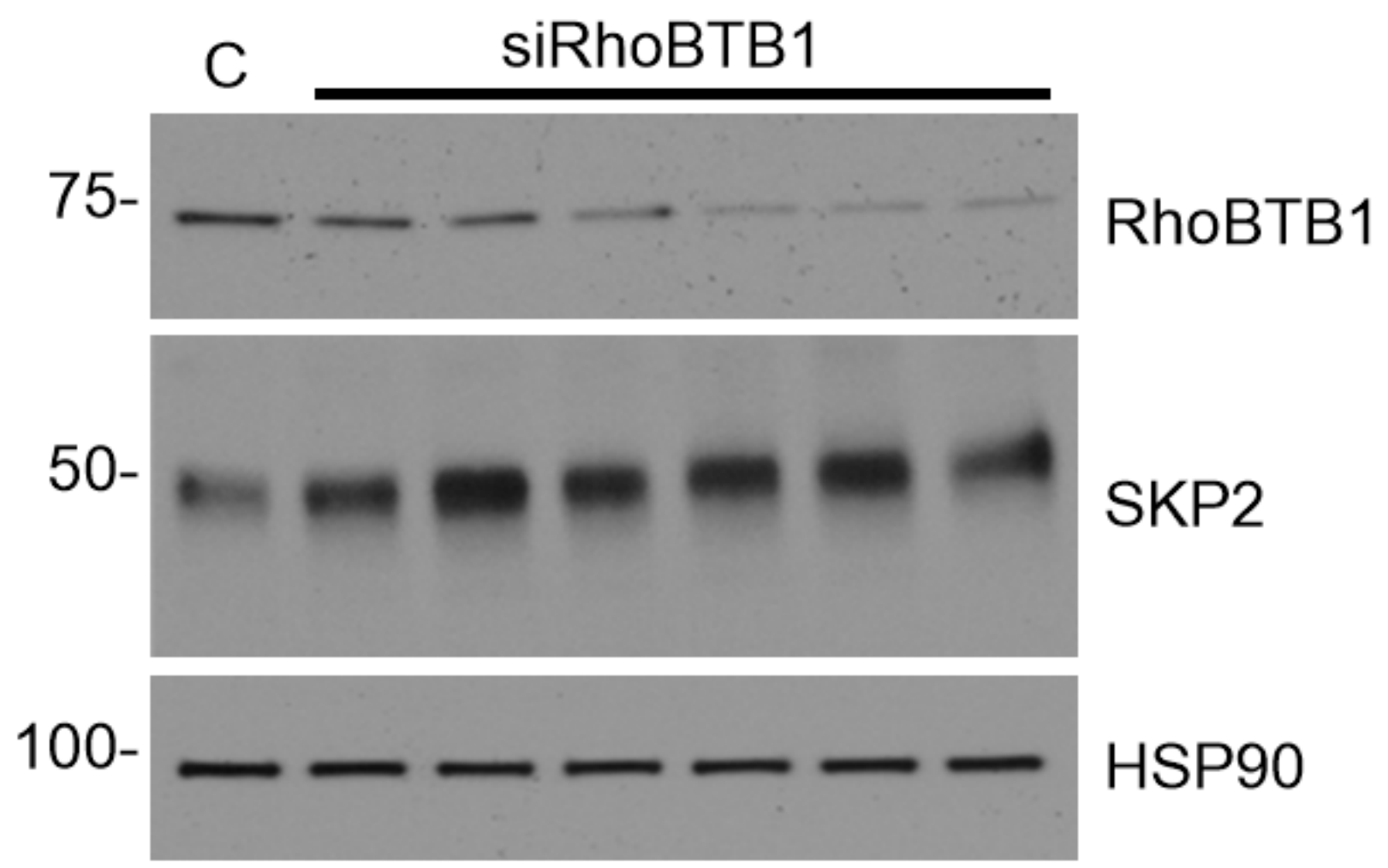

Figure 9A Raw

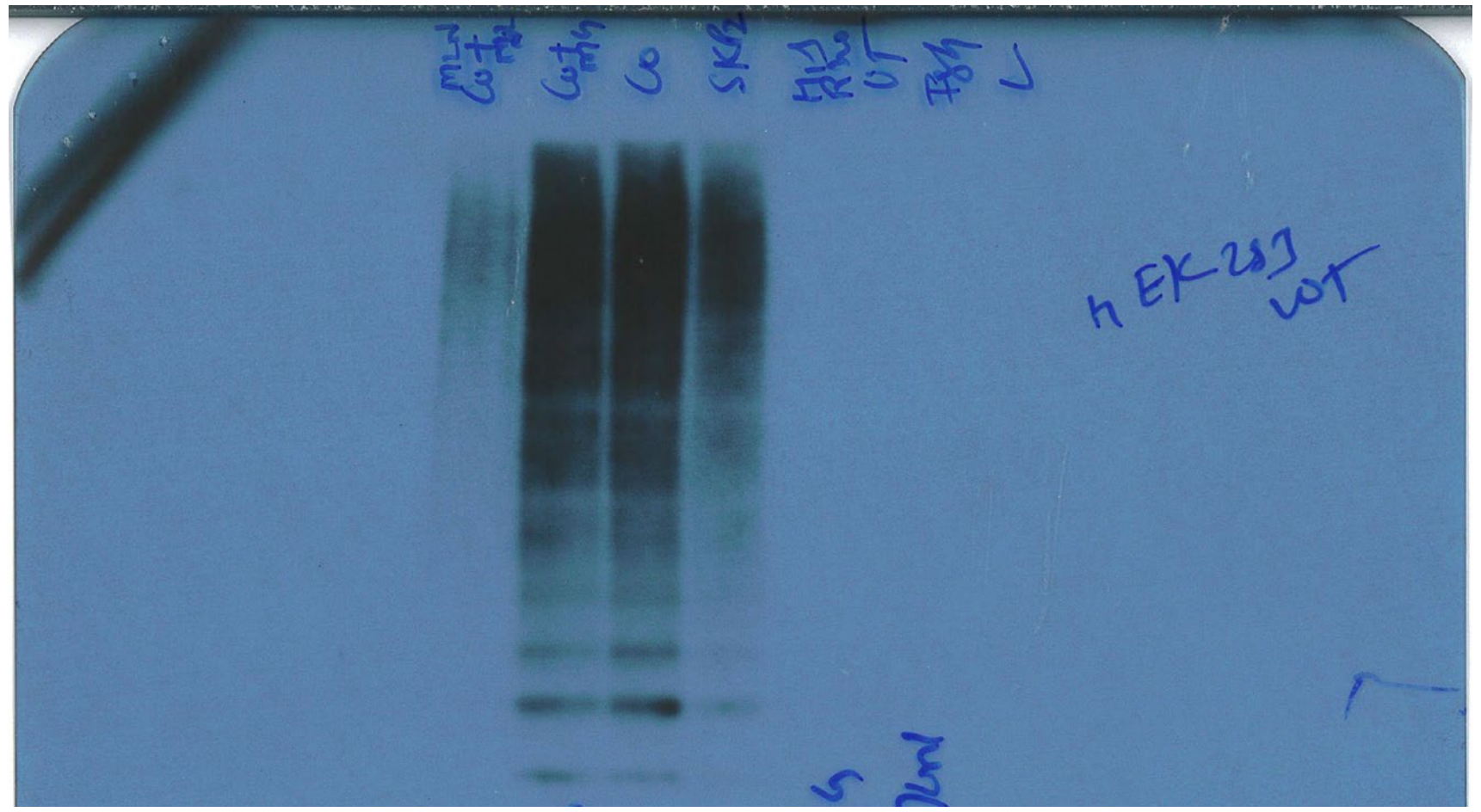

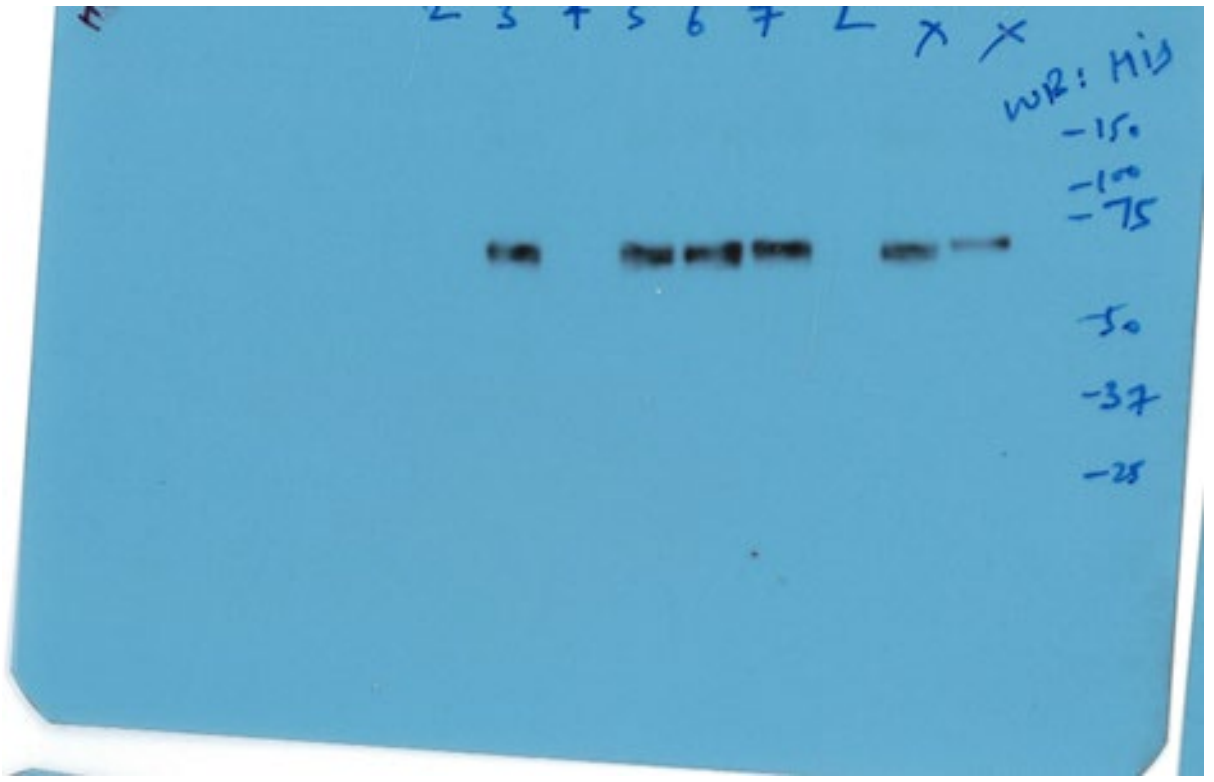

Blot # 3 : His RhoBTB1

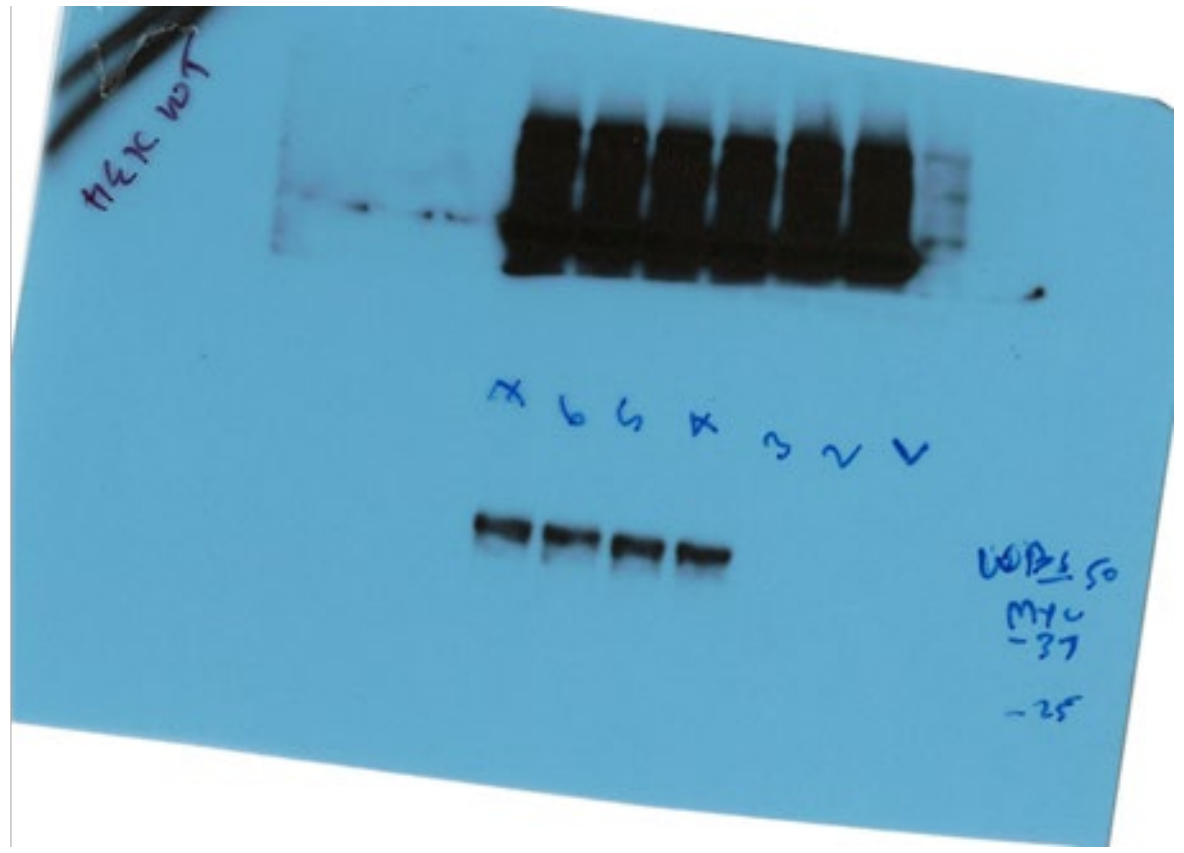

Blot # 2: SKP 2

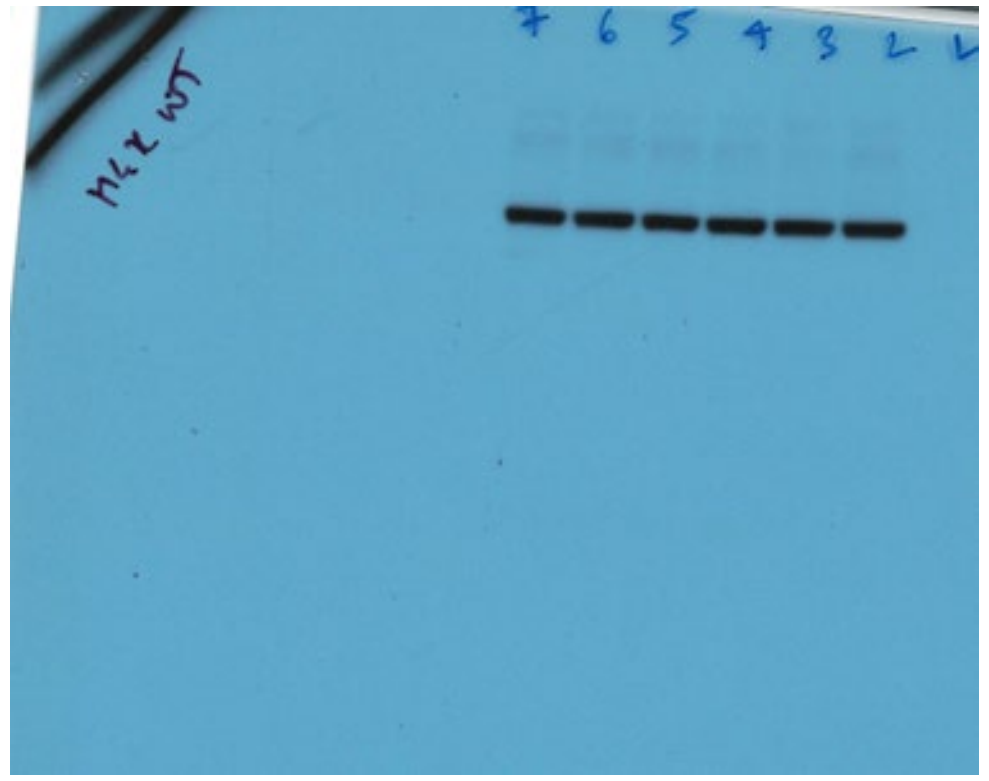

Blot # 4 : HSP 90

Figure 9A Cropped, Flipped, Rotated and Aligned

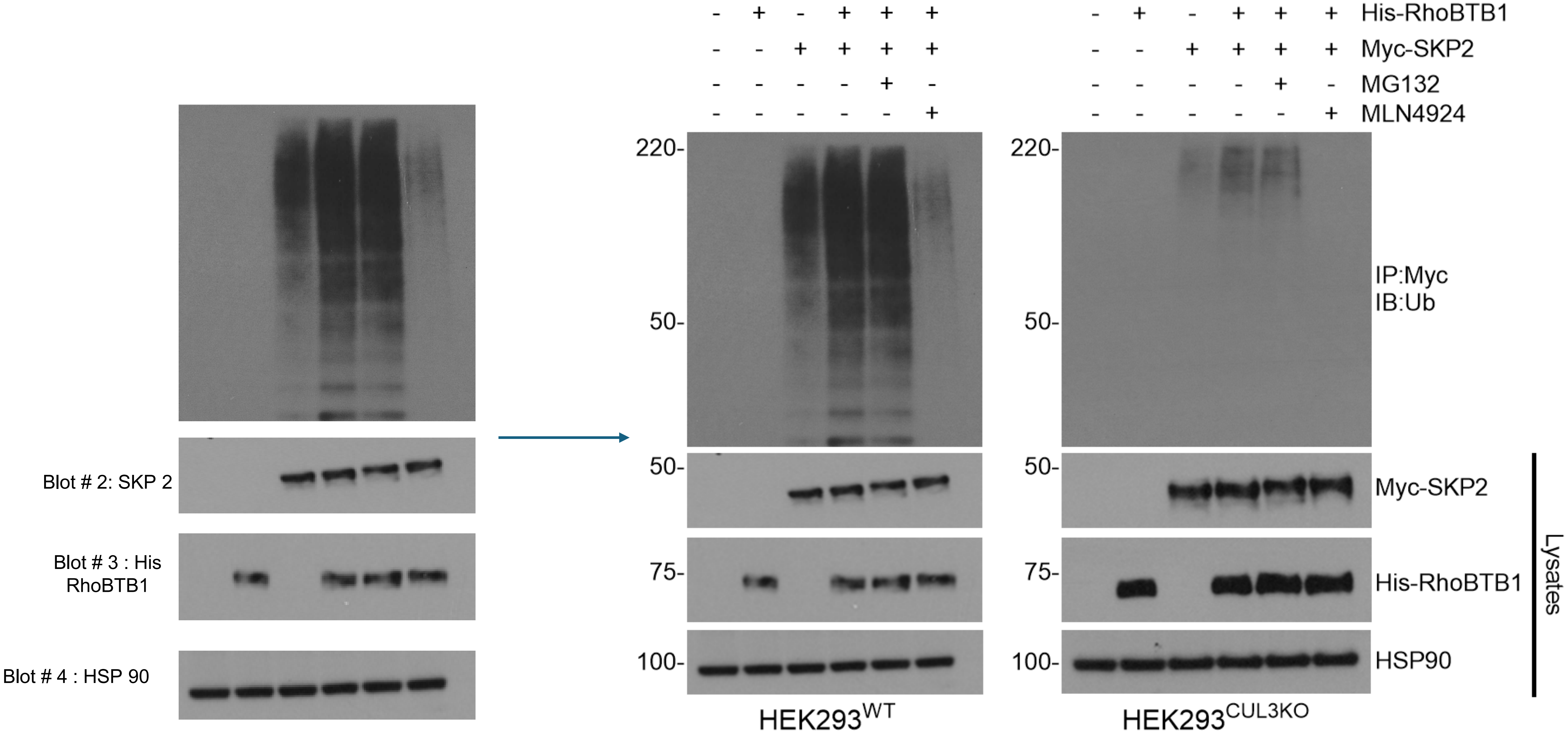

Figure 9B Raw

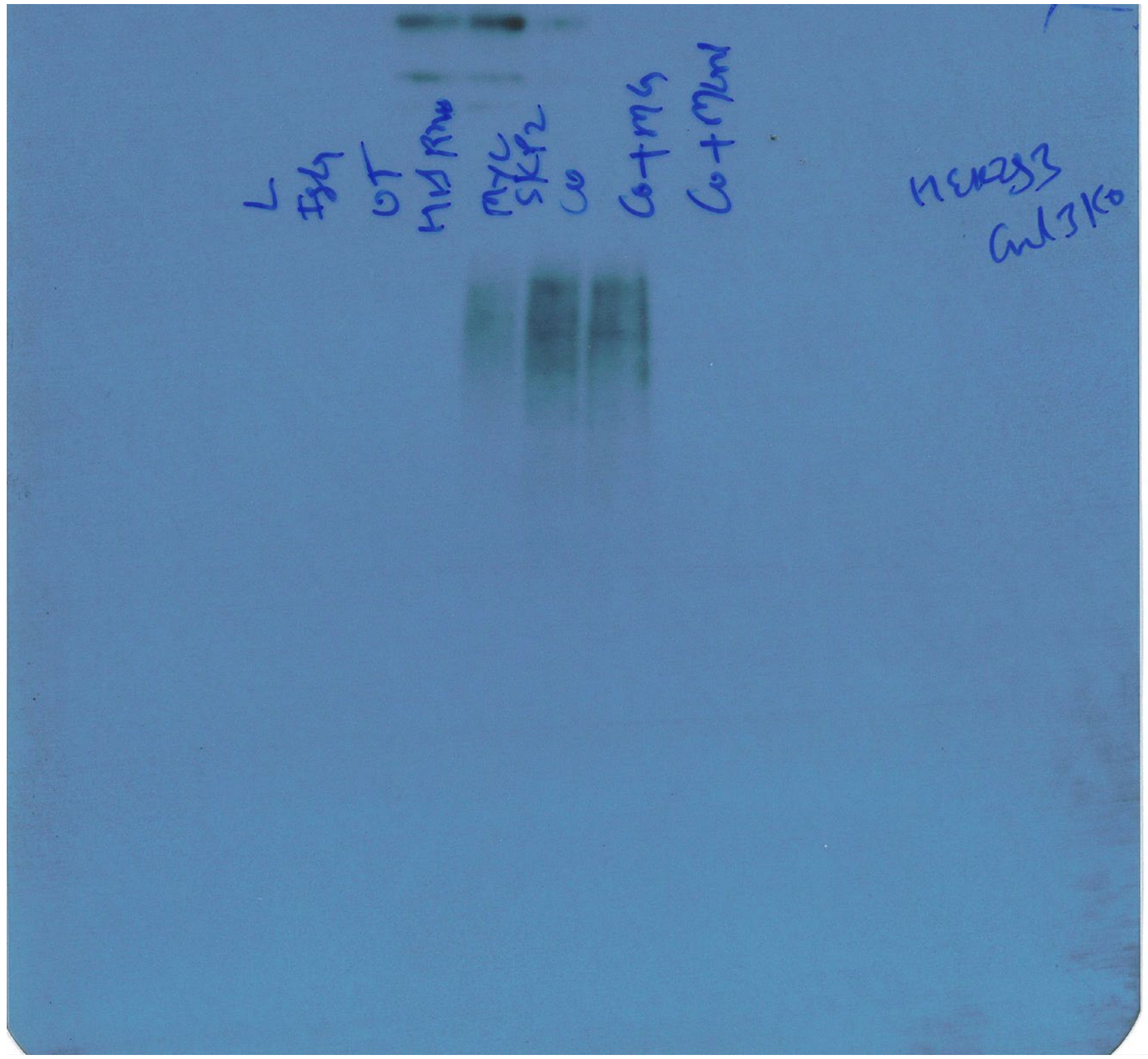

Blot # 3 : His RhoBTB1

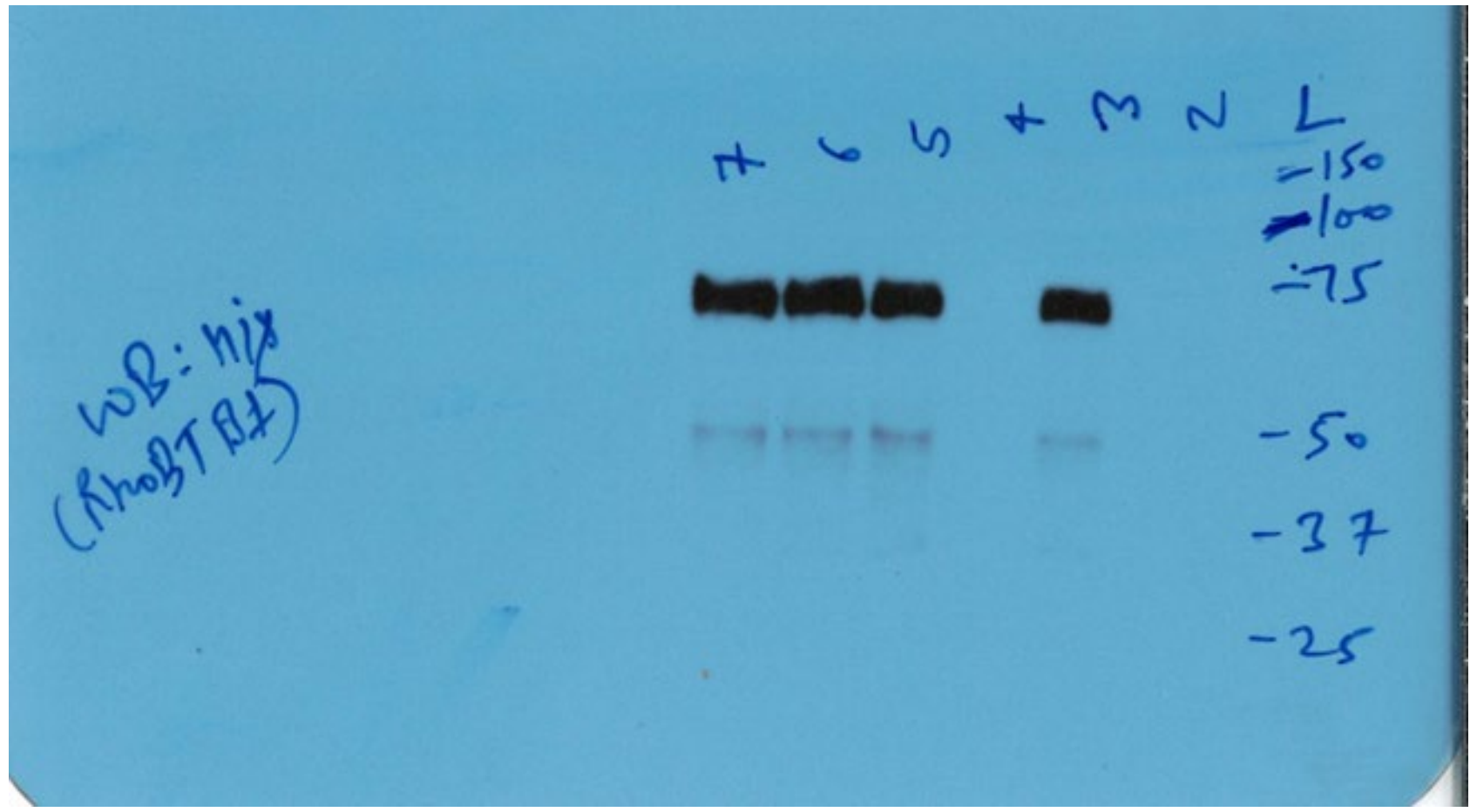

Blot # 2: SKP 2

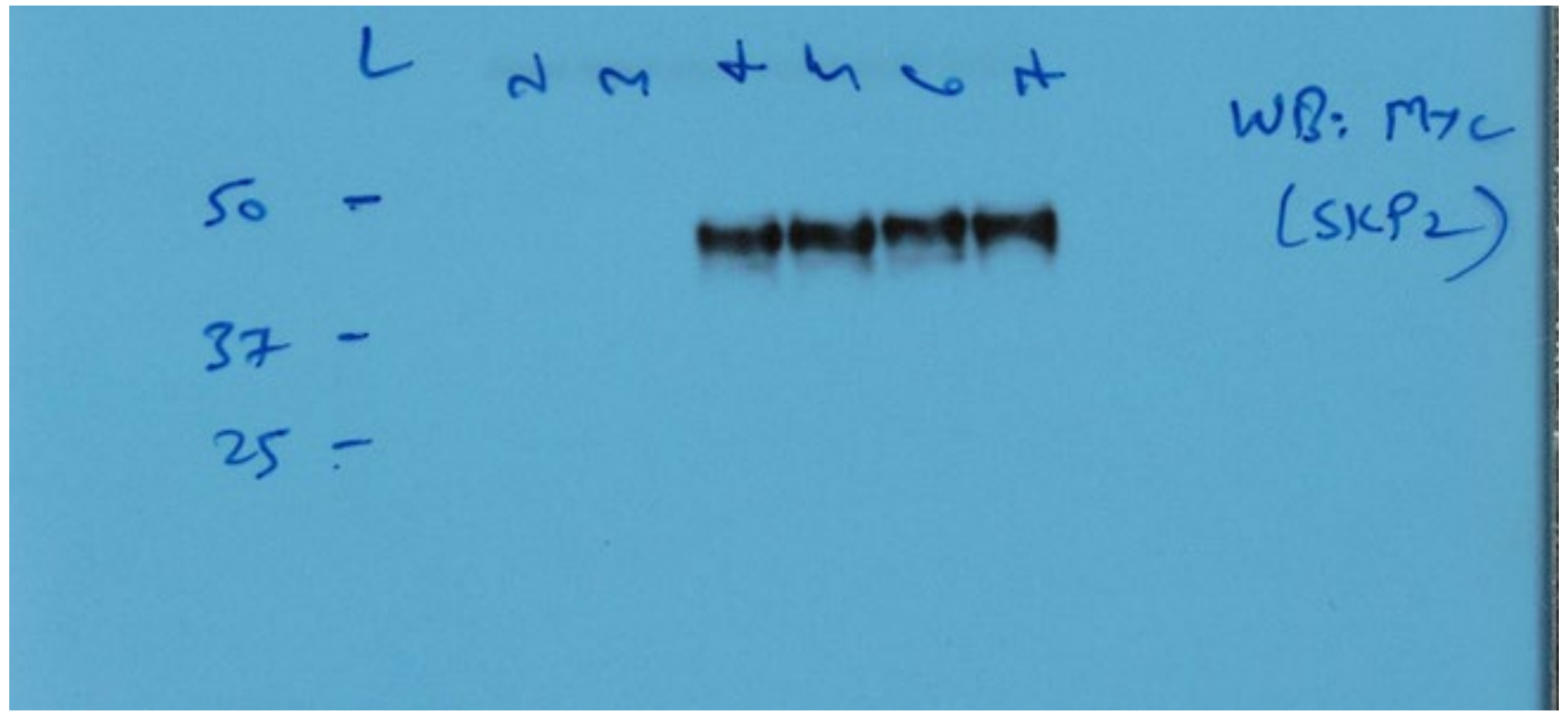

Blot # 4 : HSP 90

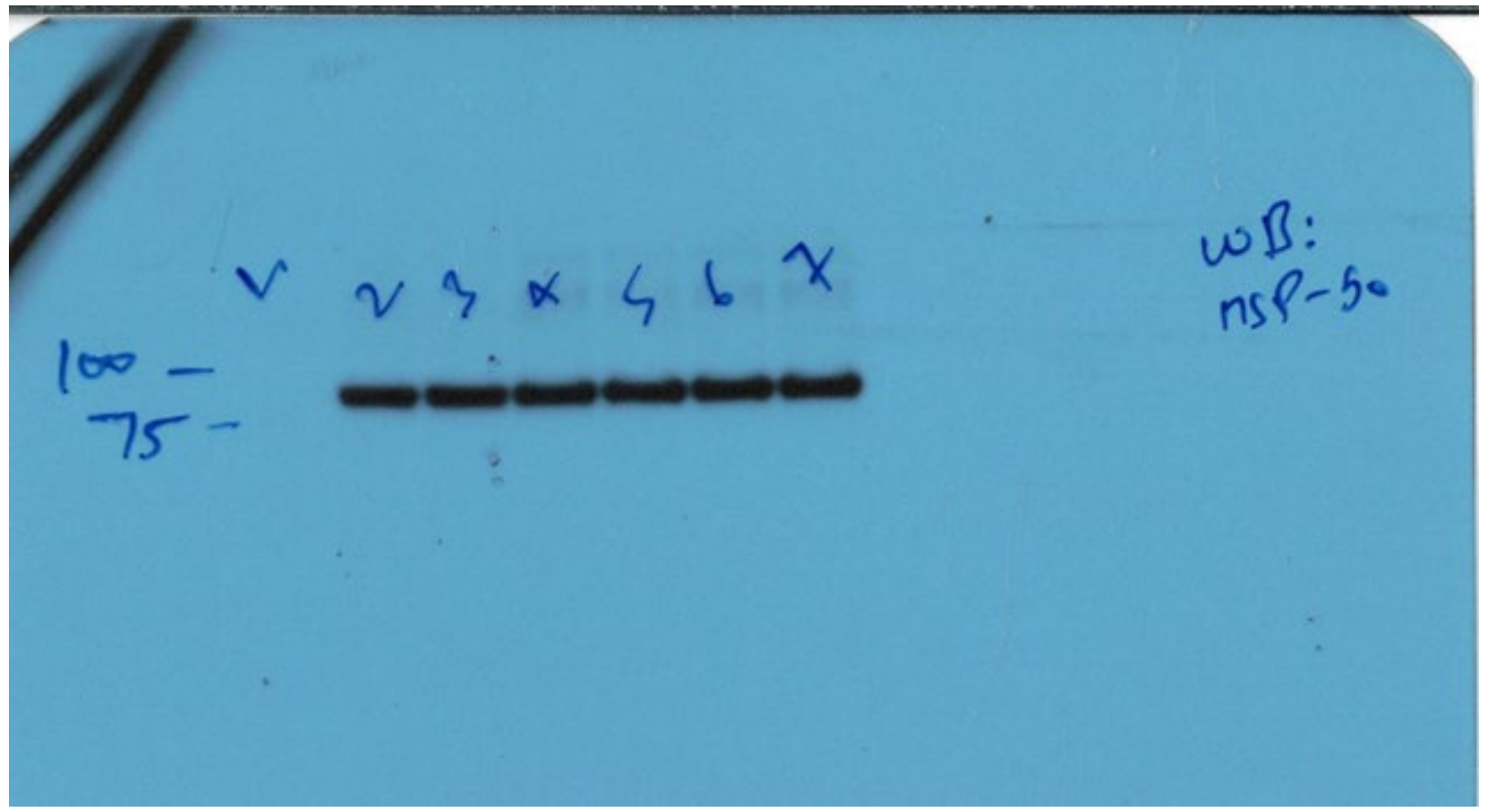

Figure 9B Cropped, Flipped, Rotated and Aligned

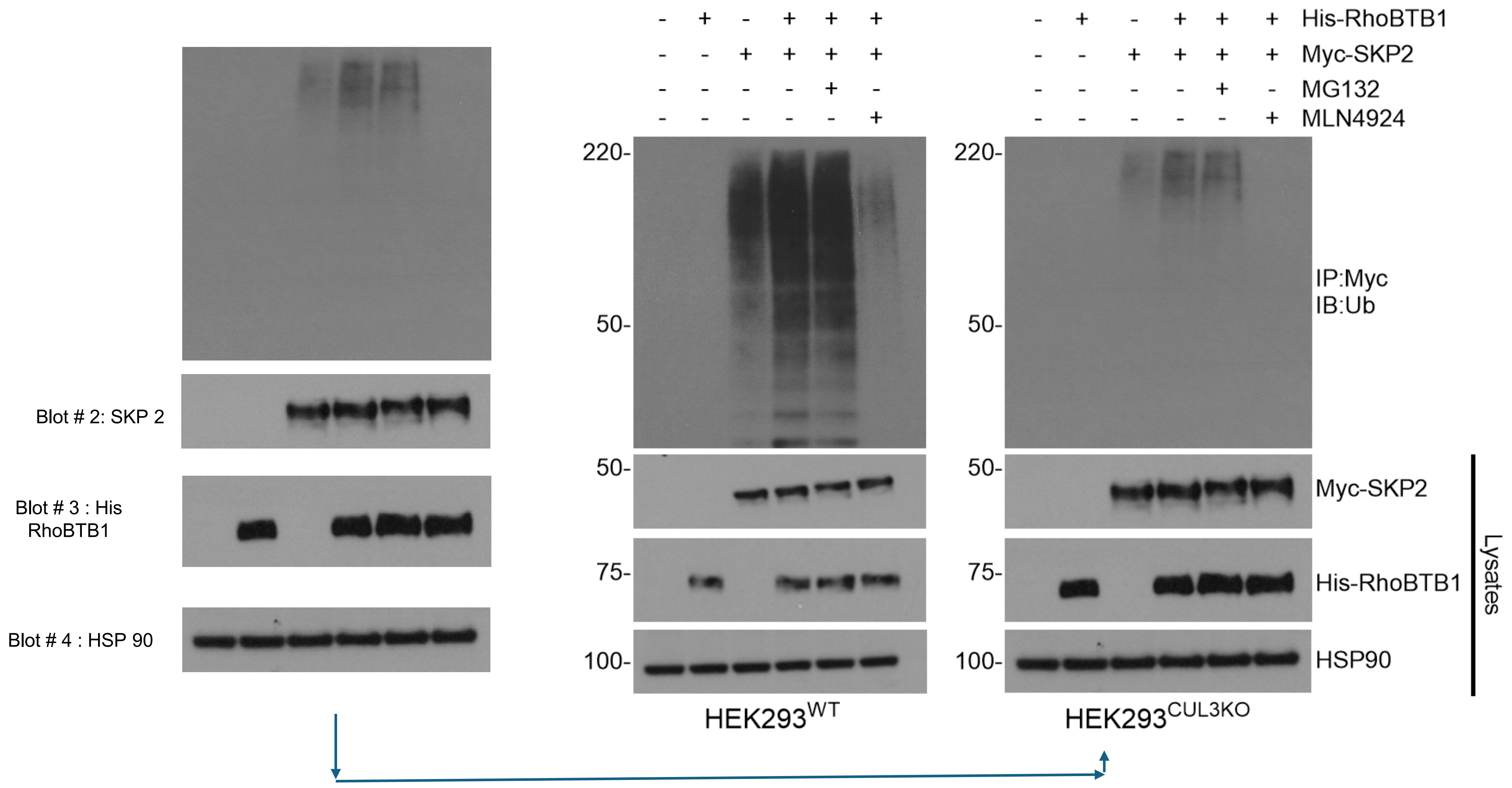

Supplement: Supplementary file 2 [file PHY2-14-e71039-s002.pdf]
